# Participant and trial characteristics reported in predictive analyses of trial attrition: an umbrella review of systematic reviews of randomised controlled trials across multiple conditions

**DOI:** 10.1186/s13063-025-08794-x

**Published:** 2025-03-12

**Authors:** Ryan McChrystal, Jennifer Lees, Katie Gillies, David McAllister, Peter Hanlon

**Affiliations:** 1 School of Health and Wellbeing, University of Glasgow, Clarice Pears Building, 90 Byres Road, Glasgow, G12 8TB UK; 2 School of Cardiovascular and Metabolic Health, University of Glasgow, BHF Glasgow Cardiovascular Research Centre, 126 University Avenue, Glasgow, G12 8TA UK; 3 Health Services Research Unit, University of Aberdeen, Health Sciences Building (3rd floor), Foresterhill, Aberdeen, AB25 2ZD UK

**Keywords:** Attrition, Dropout, Retention, Randomised, RCTs, Factors, Participant, Trial, Characteristics

## Abstract

**Background:**

Trial attrition poses several risks for the validity of randomised controlled trials (RCTs). To better understand attrition, studies have explored and identified predictors among participant and trial characteristics. Reviews of these have so far been limited to single conditions. We performed an umbrella review to explore which participant and trial characteristics are reported in predictive analyses of trial attrition in systematic reviews of RCTs across multiple conditions.

**Methods:**

We searched MEDLINE, Embase, Web of Science and the Online Resource for Research in Clinical TriAls for systematic reviews of RCTs that evaluated associations between participant/trial characteristics and attrition. We included quantitative systematic reviews of adult populations that evaluated any participant/trial characteristic and any attrition outcome. Review quality was appraised using R-AMSTAR. A review-level narrative synthesis was conducted.

**Results:**

We identified 88 reviews of RCTs evaluating characteristics associated with attrition. Included reviews encompassed 33 different conditions. Over half (50/88, 56.8%) were of RCTs for psychological conditions. All but one examined trial characteristics (87/88, 98.9%) and fewer than half (42/88, 47.7%) evaluated participant characteristics. Reviews typically reported on participant age (33/42, 78.6%), sex (29/42, 69.1%) and the type (13/42, 31%) or severity (10/42, 23.8%) of an index condition. Trial characteristics typically reported on were intervention type (56/87, 64.4%), intervention frequency/intensity (29/87, 33.3%), intervention delivery/format (26/87, 29.9%), trial duration (16/87, 18.4%), publication/reporting year (15/87, 17.2%) and sample size (15/87, 31.9%). Retention strategies were rarely reported (2/87, 2.3%). No characteristic was examined for every condition. Some reviews of certain conditions found that age (12/33, 36.4%), intervention type (29/56, 51.8%) and trial duration (9/16, 56.3%) were associated with attrition, but no characteristic was reportedly associated across multiple conditions.

**Conclusions:**

Across conditions, reviews conducting predictive analyses of attrition in RCTs typically report on several characteristics. These are participant age, sex and the type or severity of index condition, as well as the type, frequency or intensity and delivery or format of a trial intervention, trial duration, publication/reporting year and sample size. Future studies should consider exploring these characteristics as a core set when evaluating predictive factors of attrition in RCTs across multiple conditions.

**Registration:**

PROSPERO CRD42023398276.

**Supplementary Information:**

The online version contains supplementary material available at 10.1186/s13063-025-08794-x.

## Background

Trial attrition, or the non-completion of a trial for any reason, presents several risks for the validity of randomised controlled trials (RCTs). Attrition to varying degrees is an inevitable occurrence in RCTs. Publicly funded RCTs typically lose up to 12% of participants to attrition [1, 2], but rates as high as 70% have been reported [2].Considerably higher rates have been reported among RCTs for cancer [3–5], obesity [6] and psychological conditions [7–9]. The impact of attrition on trial inferences is complex and depends on the type of outcome (e.g. events versus measures), effect measures and analysis strategy (e.g. intention-to-treat (ITT) versus per protocol) [10]. Nonetheless, attrition can introduce bias if the characteristics of retained participants differ from those lost to attrition [11]. Moreover, if related to an intervention, attrition can undermine randomisation and the exchangeability of treatment groups, threatening the core strength of RCT designs [12]. Furthermore, it can impact statistical power [13], treatment effect estimates [14] and the generalisability of findings [15]. Given its commonness and potential impacts on RCTs, addressing attrition is a top priority in the trial methodology research agenda [16].

To better understand why attrition occurs in RCTs, previous studies have explored influential factors. Quantitative studies have identified participant and trial characteristics associated with attrition, including participant sex [6, 17–19] and comorbidities [17, 20], as well as trial duration [4, 5] and recruitment strategies [19]. Qualitative studies have identified participant and trialist-perceived barriers to retention in RCTs. From the participants’ perspective, these relate to personal beliefs, capabilities and life circumstances [21], while trialists perceive the presence of severe comorbidities, adverse events and study procedures to facilitate attrition [22, 23]. Evidence for influential factors has rarely been synthesised across RCTs for multiple conditions, particularly for participant and trial characteristics. Given heterogeneous trial designs, interventions and populations, the types of characteristics associated with attrition might differ depending on the condition studied. Conversely, there could be frequently associated characteristics across multiple conditions. To explore this, it would be useful to know which characteristics are reported in analyses of attrition among trials of individual conditions as well as across multiple conditions. This would inform future studies intending to evaluate characteristics that are predictive of trial attrition and, in turn, trialist decision making around retention strategies. To address this, we performed an umbrella review to identify participant and trial characteristics that are typically reported in predictive analyses of attrition among systematic reviews of RCTs across multiple conditions.

## Methods

### Protocol and registration

This systematic review was conducted according to a prespecified protocol and is reported according to the Preferred Reporting Items for Systematic Reviews and Meta-analyses (PRISMA). The PRISMA checklist is outlined in Supplementary Table 1. The protocol was registered with the International Prospective Register of Systematic Reviews (PROSPERO) prior to conducting the review (CRD42023398276). Amendments to the protocol were declared on PROSPERO and supported with explanatory statements.

### Eligibility criteria

We included any systematic review of RCTs that examined associations between participant or trial characteristics and attrition among adult populations. There were no restrictions by index condition, the types of characteristics examined, nor attrition outcomes involved. We excluded meta-analyses not embedded in or derived from systematic reviews, qualitative literature or analyses, non-English publications and reviews exclusively of phase I/II RCTs or child/adolescent populations. The PECOS framework for the eligibility criteria is outlined in Supplementary Table 2.

### Outcomes

The outcome of interest was attrition, defined as the non-completion of a trial for any reason. There is currently no standardised definition for attrition, and a variety of definitions and terms are commonly used, including dropout, premature discontinuation, premature termination and withdrawal. Additionally, there are several ways in which attrition can be summarised, including overall rate (total number of participants lost to attrition/total number of randomised participants × 100) and differential rate (total number of participants lost to attrition in the active treatment arm/total number of participants lost to attrition in the control or active comparator arm). The definitions of attrition and methods of measurement used by included reviews were captured during data extraction.

### Information sources

The following databases were searched for relevant studies: Ovid MEDLINE All (1946 to March 7th 2023), Ovid Embase (1947 to March 7th 2023), Web of Science Core Collection (1992 to March 7th 2023), The Online Resource for Research in Clinical TriAls (ORCCA) project (1986 to March 7th 2023).

### Search strategy

The search strategy was developed using an adaptation of a validated search filter developed by the Canadian Agency for Drugs and Technologies in Health (CADTH) for systematic reviews and meta-analyses [24] along with keywords describing attrition terms and quantitative data analyses. Development was supported by a university librarian. The full search strategy is outlined in Supplementary Table 3.

### Study records

Search results were exported to DistillerSR (version 2.35) for screening. We developed screening forms based on the eligibility criteria that were informally tested on 10 reviews at each screening stage.

### Selection process

Two reviewers (RM, PH) independently screened titles and abstracts in all search results. Full texts potentially meeting the eligibility criteria were retrieved and independently assessed by the two reviewers. Disagreements on the eligibility of reviews were resolved through discussion until a consensus was reached. Reasons for review exclusion were recorded.

### Data extraction

Data extraction was performed in Microsoft Excel (version 2403) using a piloted data extraction template. One reviewer (RM) independently extracted data in full. Extracted data described conditions studied, attrition outcomes, attrition metrics, review methodology, characteristics assessed for association with attrition and the magnitude and statistical significance of association.

### Quality appraisal

Review quality was appraised using the revised assessment of multiple systematic reviews (R-AMSTAR) checklist [25]. The checklist is provided in Supplementary Table 4. R-AMSTAR consists of 11 domains evaluating methodological quality, with a maximum of 4 points scored per domain and a maximum total score of 44. One reviewer (RM) independently appraised the quality of all included reviews.

### Data synthesis

We performed a descriptive data synthesis at the review level and presented a narrative synthesis to identify characteristics associated with attrition, which was supported with tables and figures. The Synthesis without Meta-analysis (SWiM) guidelines informed the synthesis [26]. Data preparation and analysis were conducted using R (version 4.3.2). Extracted data and scripts prepared for data synthesis are available at the project GitHub repository (https://github.com/RMcCPhD/umbrella_review).

Conditions and characteristics were first categorised collaboratively by two reviewers (RM, PH). Summary statistics were tabulated to describe the included reviews, including conditions studied, participant and trial characteristics reported and the outcomes of evaluating characteristics associated with attrition. Heatmaps were generated to compare the reporting of characteristics between conditions studied and the outcomes of evaluating characteristics associated with attrition. Findings were illustrated as colour-coded strata (“Associated” (green), “Not associated” (red), “Not evaluated by the review” (white)). Quality appraisal was tabulated and the distribution of R-AMSTAR scores were illustrated.

## Results

### Study selection

**Fig. 1:**
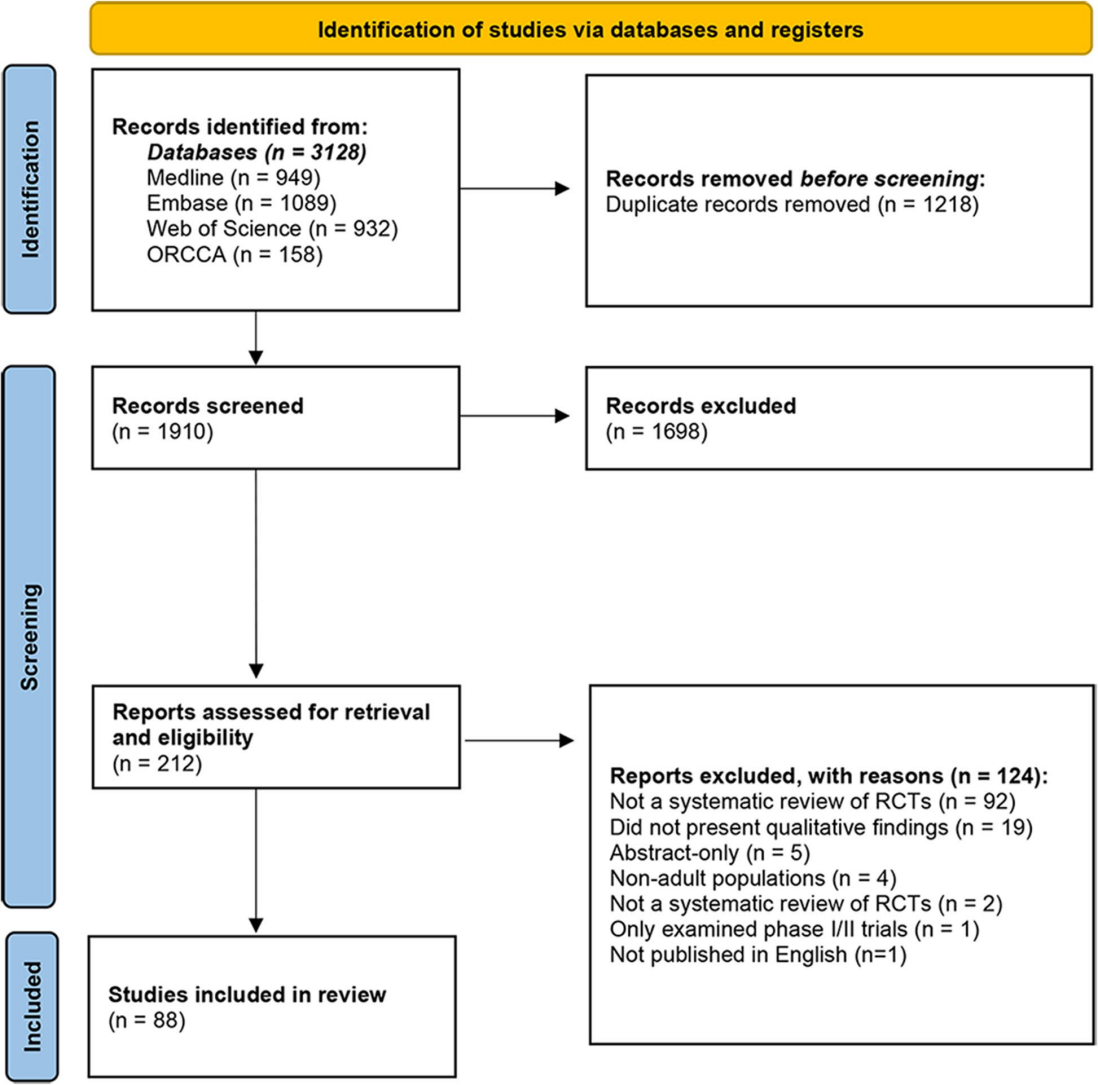
PRISMA diagram illustrating the number of reviews identified at screening stages

The PRISMA flow diagram illustrating study selection is presented in Fig. 1. Database searches returned a total of 3128 articles, of which 1218 were duplicates. Title and abstract screening excluded 1698 articles. The full texts of 212 reviews were retrieved. A total of 88 studies met the eligibility criteria and were included [27–109].

### Description of included studies

The characteristics of the included studies are presented in Table 1 and Supplementary Tables 5–8. Of the 88 reviews included, 42 (47.7%) evaluated participant characteristics and 87 (98.9%) examined trial characteristics associated with attrition. “Dropout rate” (15/88 reviews; 17.1%) and “Dropout” (11/88 reviews; 12.5%) were the most frequently used attrition terms. The majority of studies (63/88 reviews; 71.6%) did not provide a definition for the attrition outcome used. The number of RCTs included in reviews ranged from 1 to 559 (Median: 36), and the total number of participants ranged from 36 to 131,836 (Median: 4374). Over half of the included reviews were of interventions for psychological conditions (50/88 reviews; 56.8%), most frequently depression (15/50 reviews; 30%), schizophrenia (9/50 reviews; 18%) and mixed psychological disorders (8/50 reviews; 16%). The remaining reviews were mostly of cardiometabolic conditions (7/88 reviews; 8%), neurological conditions (6/88 reviews; 6.8%), mixed conditions (5/88 reviews; 5.7%) and musculoskeletal conditions (5/88 reviews; 5.7%).

**Table 1:** Included review characteristics

Review	Condition(s) studied	Number of RCTs	Participant metrics	Attrition outcome(s)	Attrition measure(s)	Attrition metrics
Albano et al. (2019) [27]	Eating disorders	22	Not reported	End of treatment dropout	Not reported	Not reported
Aparicio et al. (2016) [28]	Mixed conditions	64	N—2262	Acceptability	Total dropouts	%—6.40
Arafah et al. (2017) [29]	Multiple sclerosis	32	Not reported	Attrition or dropout rate	Attrition rate	Mean (SD)—16.5 (15)
Bacaltchuk et al. (2001)	Eating disorders	19	N—1436	Acceptability (dropout due to any cause), Tolerability (dropout due to adverse events)	Not reported	Not reported
Benbow et al. (2019) [31]	Anxiety	46	N—1057	Attrition	Not reported	Not reported
Bevens et al. (2022) [32]	Multiple sclerosis	29	Mean (SD)—97 (121.8)	Attrition	Not reported	Not reported
Bighelli et al. (2018) [33]	Schizophrenia	62	N—4068	All-cause discontinuation	Not reported	Not reported
Bricca et al. (2022) [19]	Smoking cessation	172	N—89,639	Retention rate, Differential retention rate	Not reported	Not reported
Chen et al. (2021) [34]	Tinnitus	36	N—2761	Dropout rate	Not reported	Not reported
Cooper et al. (2015) [35]	Depression	54	N—3394	Dropout rate	Weighted treatment dropout rate; Weighted study dropout rate	% [95% CI]—17.5 [16.2, 18.8]; 19.9 [18.9, 20.9]
Cramer et al. [36]	Mixed conditions	168	Median (N)—30	Dropout rate	Overall dropout rate	% [95% CI]—11.42 [10.11, 12.73]
Crutzen et al. (2015) [37]	Mixed conditions	53	Not reported	Relative attrition	Intervention attrition rate; Control attrition rate	% (SD)—18 (0.15); 17 (0.13)
Cuijpers et al. (2009) [38]	Depression	19	Not reported	Dropout	Not reported	Not reported
Cunill et al. (2013) [39]	ADHD	12	N—3375	All-cause treatment discontinuation, Proportion of patients who discontinued because of lack of efficacy, Proportion of patients who discontinued due to adverse events	Not reported	Not reported
De Campos Moreira et al. (2017) [40]	Vocal rehabilitation	51	Range (N)—9, 200	Acceptability (dropout due to any cause)	Attrition rate	Range – 10–80
DeCrescenzo et al. (2018) [41]	Addiction	50	N—6942	Dropout, Treatment dropout, Study dropout	Not reported	Not reported
Dixon et al. (2020) [42]	Mixed psychological disorders	40	Not reported	Acceptability	Dropout rate	% [95% CI]—28 [23.6, 32.9]
Doyle et al. (2021) [43]	Coronary artery disease	33	N—7240	Tolerability (withdrawal from trial)	Not reported	Not reported
Dudas et al. [44]	Dementia	9	Not reported	Primary and secondary treatment dropout, Waitlist dropout	Not reported	Not reported
Edwards-Stewart et al. (2021) [45]	PTSD	20	N—2984	Dropouts	TFT dropout rate; Non-TFT dropout rate; Waitlist dropout rate	Proportion [95% CI]—0.27 [0.21, 0.34]; 0.16 [0.12, 0.21]; 0.07 [0.02, 0.14];
Elsner et al. (2020)	Stroke	22	Not reported	Total attrition from all causes, AE-related attrition, Non-AE-related attrition	Not reported	Not reported
Fabricatore et al. (2009) [6]	Obesity	24	N—18,918	Safety (incidence of adverse events leading to withdrawal), Acceptability (withdrawal from trials)	Total attrition rate; AE-related attrition rate; Non-AE-related attrition rate	Mean (SD)—32.8 (1.6); 7.8 (0.6); 26.7 (1.5);
Frampton et al. [47]	Dementia	9	Range (N)—30,550	Dropout due to AE, Dropout for any reason	Not reported	Not reported
Furukawa et al. (2020) [48]	Depression	123	N—29,420	Dropouts	Not reported	Not reported
Gagliardi et al. [49]	Herpes zoster	24	N—88,531	Dropout due to lack of effect, Dropout due to adverse events, Total dropout	Not reported	Not reported
Gehling et al. (2011) [50]	Osteoarthritis	19	N—5861	Differential attrition	Placebo dropout rate	−38%
Goldberg et al. (2021) [51]	Mixed psychological disorders	36	Mean (SD)—143.53 (118.66)	Retention rate, Differential retention rate	Active treatment attrition; Passive treatment attrition	Mean % (SD)—23.32 (19.88); 15.36 (15.51)
Harris et al. (2021) [52]	Multimorbidity	23	N—3363	Differential attrition	Not reported	Not reported
Heneghan et al. (2007) [53]	Type 2 diabetes	27	N—5477	Attrition size	Attrition for self-monitoring blood glucose intervention; Attrition for self-monitoring blood glucose control; Attrition for self-monitoring oral anticoagulation intervention; Attrition for self-monitoring oral anticoagulation control	Range (%)—2.3–50.0; 0–40.4; 0–43.2; 0–21.4
Heo et al. (2009) [54]	Depression	68	N—8385	Dropout rate	Attrition rate	− 27.30%
Hernandez-Rodriguez et al. (2022) [55]	Cancer	17	N—6593	Safety (dropouts due to adverse events)	Overall dropout rate	% [95% CI]—9.5 [5.0, 17.5]
Hornyak et al. (2014) [56]	Restless leg syndrome	62	N—9596	Number of dropouts	Not reported	Not reported
Hrobjartsson et al. (2014) [57]	Mixed conditions	12	N—3869	Acceptability	Risk of attrition in nonblind control groups	% (Range)—7 (4–11)
Huang et al. (2014) [58]	Depression	6	N—1871	Dropout rate	Dropout rate	%— < 20
Ibrahim et al. (2016) [59]	Rheumatoid arthritis	51	Not reported	Dropout rate	Not reported	Not reported
Iliakis et al. (2021) [60]	Personality disorders	32	Not reported	Dropout	Dropout rate	% [95% CI]—27.7 [24.1, 31.7]
Imel et al. [61]	PTSD	42	N—1850	Dropout odds ratio	Active treatment dropout	% [95% CI]—18.29 [14.84, 21.75]
Jabardo-Camprubi et al. (2020) [62]	Type 2 diabetes	23	N—1684	Treatment dropout	Not reported	Not reported
Karyotaki et al. (2015) [17]	Depression	10	N—2705	Acceptability, Tolerability	Treatment dropout	− 70%
Kato et al. (2021) [63]	Depression	40	N—8890	Dropout rate	Dropout odds	OR [95% CI]—0.47 [0.40, 0.55]; 0.15 [0.79, 1.67]
Kline et al. (2021) [64]	PTSD	44	N—4866	Attrition reasons, Discontinuation from trial	Dropout rate	Mean (SD)—0.21 (0.11)
Koog et al. (2013) [65]	Knee osteoarthritis	266	Not reported	Attrition	Total dropout	N—13,593
Kredo et al. [66]	HIV	2	Not reported	Attrition rate	Not reported	Not reported
Lam et al. (2022) [67]	Mixed psychological disorders	114	N—11,288	Dropout rate	Differential attrition	OR [95% CI]—1.05 [0.92, 1.19]
Leucht et al. (2017) [68]	Schizophrenia	167	N—28,102	Dropout rate	Dropout rate	% (SD)—37.2 (20.5)
Levinson et al. (2022) [69]	PTSD	35	N—1508	Dropout	Overall dropout rate	% [95% CI]—31.3 [26.9, 36.1]
Lewis et al. (2020) [7]	PTSD	115	N—7724	Placebo dropout rate	Dropout rate	% [95% CI]—16 (14, 18)
Li et al. (2020) [70]	Depression	148	Mean (SD)—120.7 (71.5)	Dropout rate	Placebo dropout rate	Rate [95% CI]—0.25 (0.27, 0.27]
Linardon et al. (2018) [73]	Eating disorders	99	Not reported	Dropout	Dropout rate	% [95% CI]—24 [22, 27]
Linardon et al. (2019) [71]	Mixed psychological disorders	72	Not reported	Study attrition	Dropout rate	(%) [95% CI]—20.6 [17.4, 24.2]
Linardon et al. (2020) [72]	Mixed psychological disorders	70	Not reported	Dropout	Short-term follow-up attrition; Long-term follow-up attrition	% [95% CI]—24.1 [19.3, 29.6]; 35.5 [26.7, 45.3]
Makatsori et al. (2014) [74]	Allergy	81	N—9998	All-cause discontinuation of treatment, Specific discontinuation due to adverse events	Dropout rate	% [95% CI]—14 [11.9, 16]
Martin et al. (2006) [75]	Schizophrenia	28	N—7754	Dropout owing to lack of efficacy, Dropout owing to adverse events	Not reported	Not reported
Matsusaki et al. (2019) [76]	Schizophrenia	47	N—20,481	Trial retention	Not reported	Not reported
McVay et al. (2023)	Obesity	80	Median (N)—240	Dropout from treatment	Not reported	Not reported
Minozzi et al. [78]	Addiction	3	N—223	Dropout due to adverse event, Nocebo dropout	Not reported	Not reported
Mitsikostas et al. [79]	Fibromylagia	16	N—5959	Number of participants dropping out due to side effects, Total number of dropouts	Nocebo dropout	% [95% CI]—9.5 [8.3, 10.9]
Miyasaka et al. [80]	Anxiety	1	N—36	Dropout	Overall dropout rate	%—14
Ong et al. (2016) [81]	OCD	21	N—1400	Dropout	Dropout rate	% [95% CI]—17.4 [11.4, 18.4]
Ong et al. (2018) [82]	Mixed psychological disorders	68	N—4729	Participant retention, Attrition due to adverse events, Attrition due to lack of efficacy	Dropout rate	% [95% CI]—15.8 [11.9, 20.1]
Palmowski et al. (2020) [83]	Rheumatoid arthritis	243	N— > 48,000	Dropout for any reason	Not reported	Not reported
Pampallona et al. (2004) [84]	Depression	16	N—1842	Dropout due to drug-related adverse reactions (DO), Withdrawal from trial	Dropout for combined pharmacotherapy compared to psychotherapy	% [95% CI]—6.5% [− 12.1, − 1]
Papadopoulos et al. (2010) [85]	Multiple sclerosis	100	N—7355	Dropout rate	Pooled dropout rate	% [95% CI]—2.16 [1.73, 2.63]
Pozza et al. (2017) [87]	OCD	6	N—307	Total treatment arm dropout	Not reported	Not reported
Rabinowitz et al. (2009) [87]	Schizophrenia	94	N—2686	Attrition	Not reported	Not reported
Reas et al. (2008) [88]	Eating disorders	13	N—1254	All cause dropout, Dropout due to AE	Placebo dropout; Active dropout	%—31.5; 30.4
Rehman et al. (2021) [89]	Chronic pain	11	N—1015	Dropout	All cause dropout	%—28.28
Rutherford et al. (2013) [90]	Depression	111	N—29,160	Total dropout, Dropout ascribed to lack of efficacy, Dropout ascribed to adverse events, Dropout ascribed to miscellaneous factors	Dropout rate in placebo-controlled studies; Dropout rate in comparator studies	Mean (SD)—31.8 (14.1); 24.0 (10.2)
Schalkwijk et al. (2014) [91]	Depression	56	N—17,189	Dropout attrition	Not reported	Not reported
Shah et al. (2020) [92]	Smoking cessation	11	Not reported	Loss to follow-up	Not reported	Not reported
Somerson et al. (2016) [93]	Orthopaedic surgery	559	N—131,836	Retention rate	Loss to follow-up	% (Range)—10.4 (0–75)
Song et al. [94]	Cancer	55	N—12,388	Overall dropout, Dropouts due to adverse events, Dropouts due to perceived lack of drug effect	Not reported	Not reported
Stahl et al. (1993) [95]	Obesity	43	Range (N)—21, 292	Dropout rate	Not reported	Not reported
Stubbs et al. (2016) [96]	Depression	40	N—1720	Premature termination	Exercise arm dropout	% [95% CI]—15.4 [12.7, 18.5]
Swift et al. (2017) [97]	Mixed psychological disorders	182	N—17,891	Dropout from experimental intervention, Dropout from study	Premature termination rate	% [95% CI]—21.9 [20.6, 23.3]
Szymczynska et al. (2017) [98]	Schizophrenia	43	N—8640	Discontinuation rate	Intervention dropout; Study dropout	% [95% CI]—14 [13, 15]; 20 [4, 71]
Tedeschini et al. (2010) [99]	Depression	142	N—36,603	Dropout rate	Antidepressant discontinuation rate; Placebo discontinuation rate	%—31.4; 33.2
Torous et al. (2020) [100]	Depression	18	N—3336	Treatment dropout rate	App-based intervention dropout; Placebo app intervention dropout rate; Waitlist intervention dropout rate	% [95% CI]—26.2 [18.12, 36.34]; 25.1 [11.34, 46.75]; 20.45 [5.14, 54.9]
Vancampfort et al. (2016) [8]	Schizophrenia	19	N—1177	Treatment dropout rate	Physical activity dropout; Inactive intervention dropout	% [95% CI]—22.1 [16.4, 28.9]; 12.7 [7.7, 20.2]
Vancampfort et al. (2017) [101]	HIV	36	N—1128	Treatment dropout rate	Physical activity dropout	% [95% CI]—22.6 [18.9, 26.9]
Vancampfort et al. (2021) [102]	Anxiety	14	Not reported	Dropout	Dropout rate	% [95% CI]—15.7 [9.9, 23.9]
Villeneuve et al. (2010) [103]	Schizophrenia	74	N—4374	Number of dropouts	Dropout rate	% [95% CI]—13 [0.106, 0.156]
Wahlbeck et al. (2001) [104]	Schizophrenia	163	N—18,585	Loss to follow-up	Not reported	Not reported
Wasmann et al. (2019) [105]	Mixed conditions	20	Not reported	Overall dropout rate, dropouts due to adverse events), Dropouts due to adverse events	Not reported	Not reported
Weinmann et al. (2008) [106]	Depression	17	N—3485	Dropout rate	Venlafaxine dropout; SSRI dropout	N/Total—413/1748; 392/1791
Windle et al. (2020) [107]	Mixed psychological disorders	16	N—1857	Rate of premature discontinuation	Preferred accommodation dropout; Unpreferred accommodation dropout	%—18.8; 33.5; 18.8; 33.5
Zhang et al. (2021) [108]	Acute mania	3	N—174	Dropout	Not reported	Not reported
Zhou et al. (2020) [109]	PTSD	18	N—1422	Dropout rate	Not reported	Not reported

### Quality appraisal

Quality appraisal and distribution of scores for the included reviews can be found in Supplementary Table 9 and Supplementary Fig. 1. Total scores were normally distributed, with a mean (SD) of 28 [5]. Reviews scored best in domain 3 (“Was a comprehensive literature search performed?”, four points—50/88 reviews; 56.81%), domain 2 (“Was there duplicate study selection and data extraction?”, four points—41/88 reviews; 46.59%), domain 7 (“Was the scientific quality of the included studies assessed and documented”, four points—41/88 reviews; 46.59%), domain 6 (“Were the characteristics of the included studies provided?”, four points—39/88 reviews; 44.32%) and domain 1 (“Was an ‘a priori’ design provided?”, three points—60/88 reviews; 68.18%). Reviews scored poorest in domain 8 (“Was the scientific quality of the included studies used appropriately in formulating conclusions?”, 1 point—63/88 reviews; 71.6%), domain 10 (“Was the likelihood of publication bias assessed?”, 1 point—32/88 reviews; 36.36%), domain 5 (“Was a list of studies (included and excluded) provided?”, 2 points—44/88 reviews; 50%), domain 4 (“Was the status of publication (i.e. grey literature) used as an inclusion criterion?”, 2 points—42/88 reviews; 47.72%) and domain 11 (“Was the conflict of interest included?”, 2 points—42/88 reviews; 47.72%).

### Characteristics associated with attrition

#### Participant characteristics

**Fig. 2:**
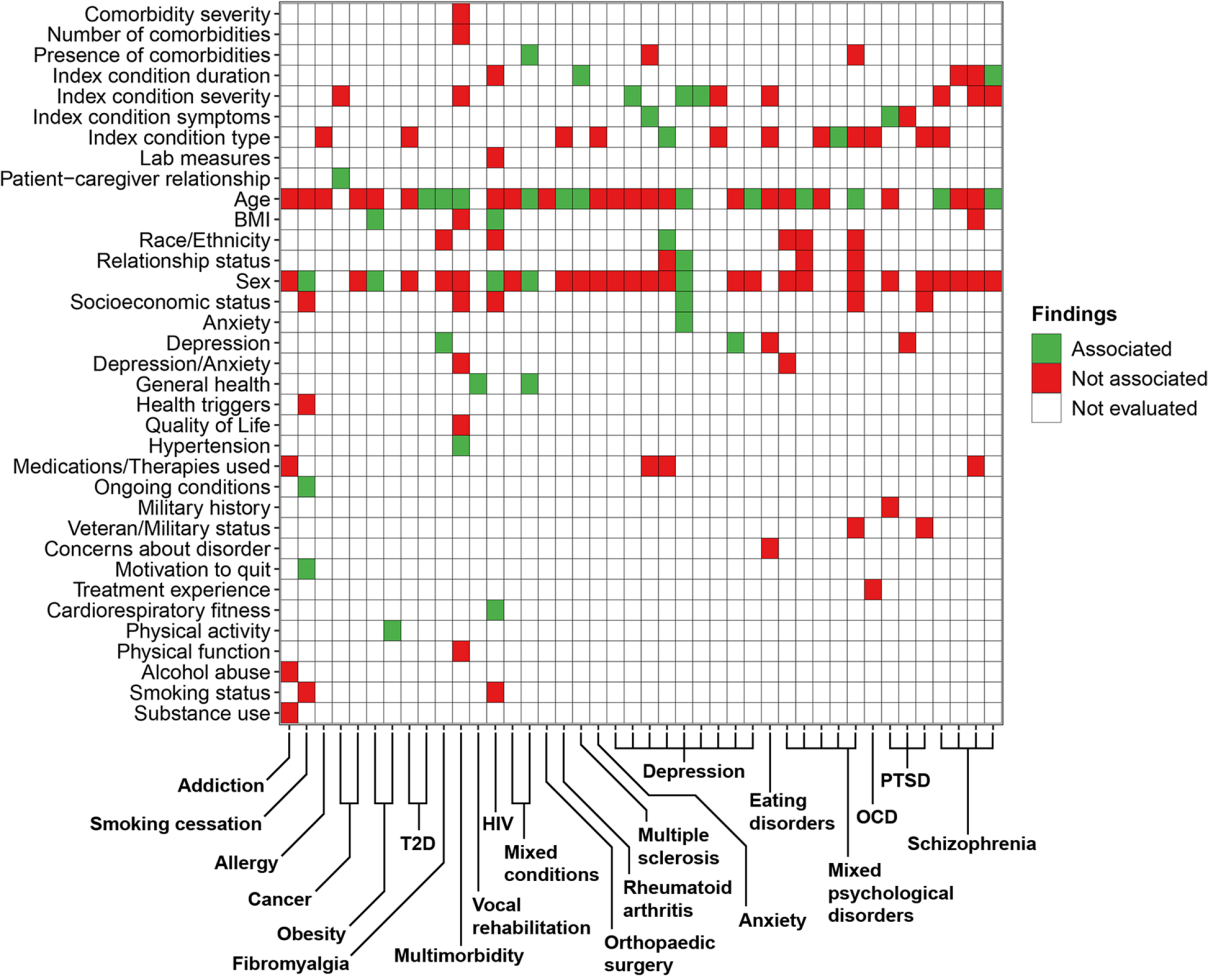
Heatmap of reported participant characteristics and outcome of evaluating associations. Individual reviews are indicated by *x*-axis ticks and are grouped by condition using single or bracketed labels

Findings from the 42 reviews that evaluated participant characteristics are summarised in Fig. 2 and Supplementary Tables 10 and 11. Demographics (34/42 reviews; 81%) and condition-related (26/42 studies; 61.9%) characteristics were most frequently reported in evaluations. Across all conditions, age (33/42 reviews; 78.6%, 19/21 conditions; 90.5%), sex (29/42 reviews; 69.1%, 16/21 conditions; 76.2%) and index condition type (13/42 reviews; 31%, 10/21 conditions; 47.6%) and index condition severity (10/42 reviews; 23.8%, 5/21 conditions; 23.8%) were reportedly the most frequently evaluated. None of these characteristics were reportedly associated with attrition across multiple conditions. Associations between attrition and age (12/33 reviews; 36.4%) and index condition severity (3/10 reviews; 30%) were reported among some conditions. Age was reportedly associated with attrition among reviews of fibromyalgia, multimorbidity, mixed conditions, arthritic conditions, mixed psychological disorders and schizophrenia. Associations reportedly between attrition and index condition severity were limited to reviews of RCTs in depression (3/4 reviews; 75%).

Beyond age, sex and the type or severity of index conditions, the characteristics examined among reviews for different conditions were heterogeneous. Reviews of multimorbidity (11 characteristics describing demographics, medical history, index condition, physical function, health/wellbeing and depression/anxiety), HIV (9 characteristics describing demographics, physical function, index condition and substance use) and smoking cessation (7 characteristics describing demographics, health/wellbeing, medical history, perceptions/motivations and substance use) evaluated a greater variety of characteristics compared to other conditions, but few variables were reportedly associated with attrition. Reviews of depression, mixed psychological disorders and schizophrenia evaluated a greater variety of index condition-related characteristics. Additionally, race/ethnicity (findings from race or ethnicity combined across included reviews) was frequently evaluated among reviews of mixed psychological disorders, but associations were not reported in any instance. Few associated characteristics were reported among reviews of mixed psychological disorders and schizophrenia.

#### Trial characteristics

**Fig. 3:**
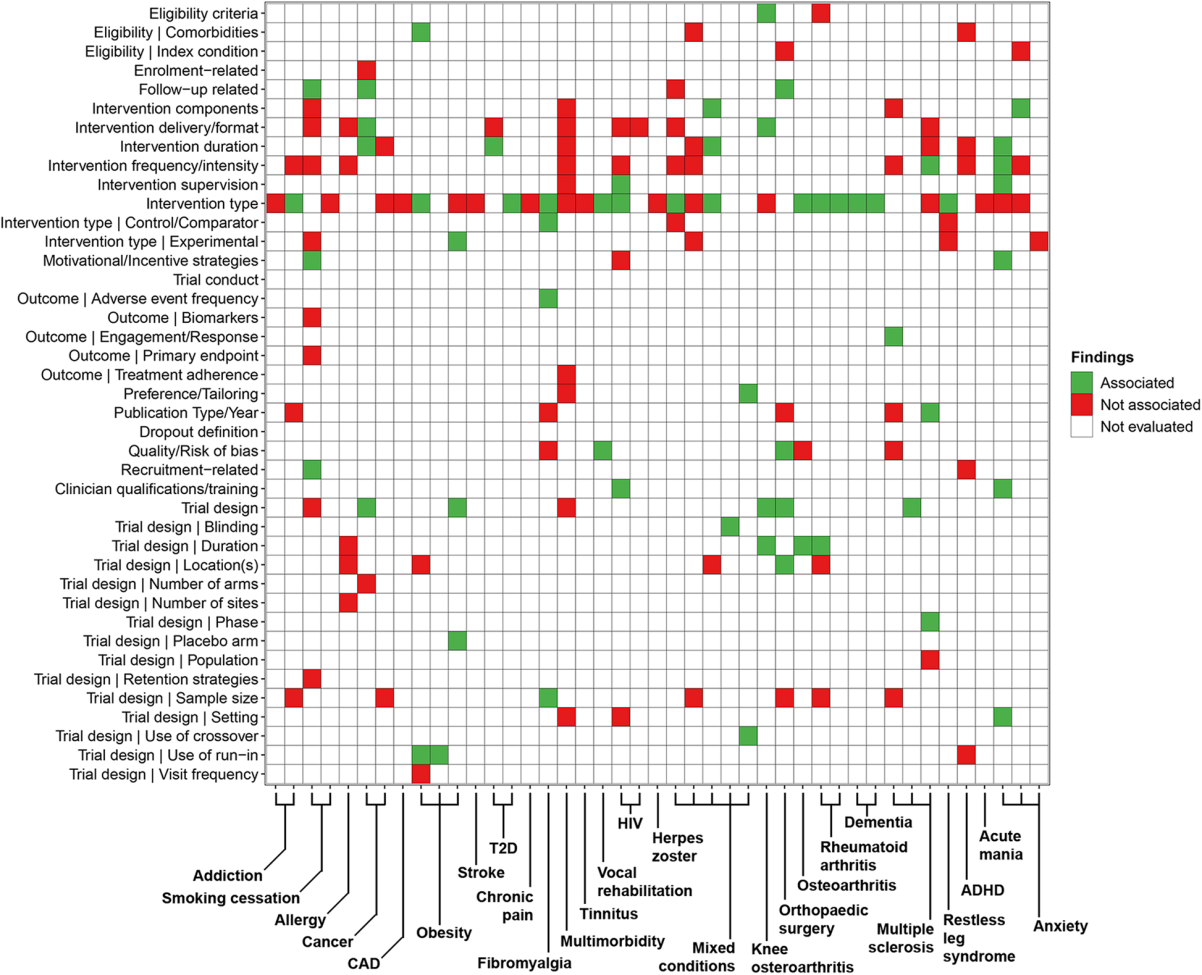
Heatmap of reported trial characteristics and outcome of evaluating associations. Individual reviews for 26 of 33 conditions are indicated by *x*-axis ticks and are grouped by condition using single or bracketed labels

**Fig. 4:**
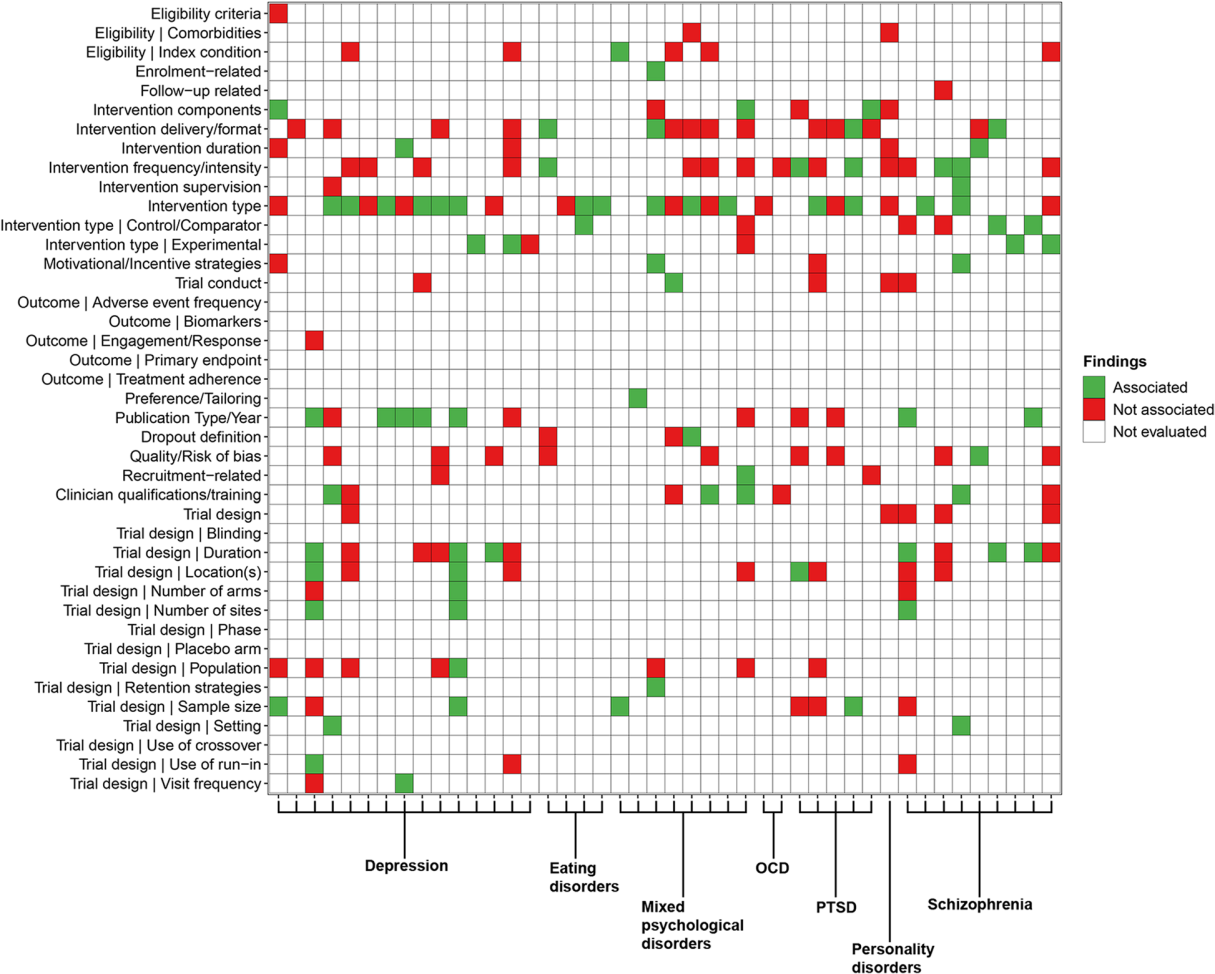
Additional heatmap of reported trial characteristics and outcome of evaluating associations. Individual reviews for the remaining 6 conditions are indicated by *x*-axis ticks and are grouped by condition using single or bracketed labels

Findings from 87 reviews that evaluated trial characteristics can be found in Figs. 3 and 4 and Supplementary Tables 12 and 13. Intervention-related (79/87 reviews; 90.8%) and trial design-related (47/87 reviews; 54%) characteristics were most frequently reported in evaluations. Retention strategies were seldom evaluated (2/87 reviews; 2.3%, 2/33 conditions; 6.1%). Across all conditions, intervention type (56/87 reviews; 64.4%, 30/33 conditions; 90.9%), intervention frequency/intensity (29/87 reviews; 33.3%, 16/33 conditions; 48.5%), intervention delivery/format (26/87 reviews; 29.9%, 14/33 conditions; 42.4%), trial duration (16/87 reviews; 18.4%, 6/33 conditions; 18.2%), publication/reporting year (15/87 reviews; 17.2%, 7/33 conditions; 21.2%) and sample size (15/87 reviews; 31.9%, 11/33 conditions; 33.3%) were reportedly the most frequently evaluated trial characteristics. None of these characteristics were reportedly associated with attrition across multiple conditions. Associations between attrition and intervention type (29/56 reviews; 51.8%), trial duration (9/16 reviews; 56.3%) and publication/reporting year (8/15 reviews; 53.3%) were reported among some conditions. Intervention type was reportedly associated with attrition among reviews of arthritic conditions, depression, eating disorders and mixed psychological disorders. Trial duration was reportedly associated among reviews of arthritic conditions, depression and schizophrenia. Associations for publication/reporting year were mostly reported among reviews of depression (5/6 reviews; 83.3%).

Groups of evaluated characteristics were identified among reviews of different conditions. Attrition outcomes were evaluated as a trial characteristic in three reviews, with one review of RCTs of mixed psychological disorders reporting an association with attrition. Reviews of obesity frequently evaluated the use of a run-in period and associations with attrition were reported. Outcome measures were evaluated among reviews of smoking cessation and fibromyalgia, but in most instances were not reportedly associated with attrition. The frequency of adverse events as an outcome was reportedly associated with attrition in one fibromyalgia review. Reviews of multiple conditions and anxiety, depression, mixed psychological disorders, PTSD and schizophrenia evaluated a greater variety of intervention-related characteristics than reviews of other conditions, but reports of association were mixed. Additionally, depression reviews frequently evaluated trial populations, but few reported associations with attrition. Schizophrenia reviews reported a greater number of associations with attrition among intervention-related characteristics.

## Discussion

### Principal findings

In a review of 88 systematic reviews of RCTs for 33 different conditions, we found that predictive analyses of trial attrition were most frequently conducted for RCTs of psychological conditions. Trial characteristics were most commonly evaluated as predictive factors among all reviews, and less than half evaluated participant characteristics. Reviews evaluating participant characteristics tended to evaluate age, sex and the type and severity of an index condition. Beyond these, the types of participant characteristics evaluated varied between conditions. Reviews evaluating trial characteristics tended to evaluate the type of intervention received, its frequency or intensity and its delivery or format, as well as trial duration, publication/reporting year and sample size. Retention strategies were rarely evaluated. Clusters of frequently evaluated trial characteristics were identified among reviews of several conditions. No participant or trial characteristic was consistently reported to be associated with attrition across all conditions. Associations between attrition and participant age, the type of intervention received and trial duration were reported among several conditions, but only among reviews of arthritic conditions were all three reportedly associated with attrition.

### Strengths and limitations

To our knowledge, this is the first study to explore the types of characteristics evaluated in predictive analyses of attrition in RCTs for multiple conditions, and the first to do so by synthesising evidence from systematic reviews. This was supported by a thorough review that used a validated search strategy built on extensively tested and routinely used search filters [24], duplicate and independent study screening and rigorous descriptive analysis and synthesis.

However, there were several limitations. First, our broad approach to the review eligibility criteria resulted in substantial heterogeneity among the methodologies and findings of included reviews. Consequently, we were unable to meta-analyse associations between characteristics and trial attrition. Secondly, by including only systematic reviews and meta-analyses derived from systematic reviews, we have omitted RCTs where characteristics that predict trial attrition might have been explored but are not yet synthesised in an existing systematic review. Additionally, our restriction to published reviews omitted reviews in grey literature. Thirdly, the majority of included studies were of psychological conditions, the results of which are not true of RCTs more generally. Lastly, quality appraisal indicated that most included reviews did not consider the quality of included RCTs when formulating conclusions, and less than half conducted a thorough quality assessment.

### Strengths and limitations in relation to other studies

Minimising attrition is a top priority in the trial methodology research agenda [16], and a number of studies have explored influential factors of attrition. Evidence for influential factors has rarely been synthesised across RCTs for multiple conditions, and it is possible that different factors are associated with attrition across conditions. Our review adds to the literature by identifying several characteristics that are typically evaluated in predictive analyses of attrition, and that no characteristic has been reportedly associated with attrition across multiple conditions.

Included reviews that evaluated participant characteristics used aggregate-level data, and findings were similar to studies that previously analysed individual participant data (IPD). For example, our finding that age was not reportedly associated with attrition across conditions aligns with the findings of Lees et al. [20], who in a meta-analysis using IPD from 92 RCTs across 20 conditions found age not to be associated with attrition. Some conditions were present in both studies, such as type 2 diabetes and rheumatoid arthritis, but our review included a greater variety of conditions excluded by Lees et al., such as cancer and psychological conditions. Furthermore, our eligibility criteria allowed for reviews of RCTs regardless of funding sources, while Lees et al. focussed solely on industry-funded trials. However, we did not distinguish included reviews by funding source, nor did we conduct a meta-analysis and could not make a definitive conclusion on reported associations between attrition and age.

### Meaning of the study

Our findings demonstrate that there is little information available to trialists on what predicts attrition in trials across multiple conditions. Consequently, it is uncertain whether targeting aspects of trials and populations would be worthwhile when seeking retention strategies that are broadly applicable to trials. Using meta-analysis, further exploration of associations between participant and trial characteristics and attrition that are observed in trials for the likes of depression, multimorbidity and arthritic conditions could provide future targets for retention strategies. Our review is the first to explore characteristics involved in predictive analyses of trial attrition across multiple conditions, and we hope our findings prompt future studies to conduct further investigation.

### Unanswered questions and future research

From our findings, it is apparent that trial characteristics are much more frequently evaluated as predictors of attrition than participant characteristics. Where participant characteristics were evaluated, none of the included reviews used IPD in their analyses. One possible explanation for this is that trial-level data are more readily available and easier to collect, extract and analyse. Conversely, the steps to acquiring IPD and producing research outputs are time-consuming [110]. Despite this, a number of studies have conducted IPD analyses and identified characteristics that predict attrition [4, 111, 112]. Given the granular detail provided by IPD, future studies intending to evaluate participant characteristics that predict attrition should plan for the use of IPD.

Our review indicates that the information available to trials on what predicts attrition is limited, but that there are several characteristics that are typically evaluated when conducting predictive analyses. For consistency, future studies evaluating predictive factors of attrition should consider evaluating and reporting on these typical characteristics. This would allow for a meta-analysis to identify characteristics that are predictive of attrition among RCTs of different conditions. Following on from our review, we are conducting a network meta-analysis using IPD from RCTs for a heterogeneous set of conditions to develop a risk prediction model for trial attrition. In this, we will explore characteristics that we found to be typically reported among included reviews.

## Conclusion

In this umbrella review of studies conducting predictive analyses of attrition in RCTs of different conditions, we identified several characteristics that were typically evaluated. These are participant age, sex and the type or severity of an index condition, as well as the type, frequency or intensity and delivery or format of a trial intervention, trial duration, publication/reporting year and sample size. Future studies investigating predictive factors of attrition in trials across multiple conditions should consider exploring these characteristics. We found no participant or trial characteristic to be reportedly associated with attrition across conditions. Despite the importance of mitigating attrition, it is apparent that the types of characteristics that predict attrition are largely unknown.

## Supplementary Information

Additional file 1. Tables and figures providing additional information for the methods and results of this umbrella review. Supplementary Table 1. PRISMA checklist. Supplementary Table 2. Eligibility criteria. Supplementary Table 3. Search strategy used for each database. Supplementary Table 4. R-AMSTAR checklist. Supplementary Table 5. Additional review characteristics describing attrition outcomes and definitions. Supplementary Table 6. Additional review characteristics describing the evaluation of participant characteristics. Supplementary Table 7. Additional review characteristics describing the evaluation of trial characteristics. Supplementary Table 8. Summary of conditions studied by included reviews. Supplementary Table 9. Summary of quality appraisal using R-AMSTAR. Supplementary Fig. 1. Frequency of R-AMSTAR total and domain scores. Supplementary Table 10. Summary of participant characteristics evaluated by included reviews. Supplementary Table 11. Summary of participant characteristics evaluated among conditions studied. Supplementary Table 12. Summary of trial characteristics evaluated by included reviews. Supplementary Table 13. Summary of trial characteristics evaluated among conditions studied

## Data Availability

Extracted data and R scripts created for the analysis are available at the project GitHub repository (https://github.com/RMcCPhD/umbrella_review).

## Abbreviations

CADTH: Canadian Agency for Drugs and Technologies in Health
HIV: Human immunodeficiency virus
IPD: Individual participant data
ITT: Intention-to-treat
ORCCA: Online Resource for Research in Clinical TriAls
PECOS: Population, Exposure, Comparator, Outcome, Study
PRISMA: Preferred reporting items for systematic reviews and meta-analyses
PROSPERO: Prospective register of systematic reviews
PTSD: Posttraumatic stress disorder
R-AMSTAR: Revised assessment of multiple systematic reviews
RCT: Randomised controlled trial
SWiM: Synthesis without Meta-analysis
