# Supplementary material for: Participant and trial characteristics reported in predictive analyses of trial attrition: an umbrella review of systematic reviews of randomised controlled trials across multiple conditions
Source: Trials. 2025 Mar 12;26:84. doi: 10.1186/s13063-025-08794-x (PMC11900635; doi:10.1186/s13063-025-08794-x)
Supplement: Supplementary file 1 — Additional file 1. Tables and figures providing additional information for the methods and results of this umbrella review. Supplementary Table 1. PRISMA checklist. Supplementary Table 2. Eligibility criteria. Supplementary Table 3. Search strategy used for each database. Supplementary Table 4. R-AMSTAR checklist. Supplementary Table 5. Additional review characteristics describing attrition outcomes and definitions. Supplementary Table 6. Additional review characteristics describing the evaluation of participant characteristics. Supplementary Table 7. Additional review characteristics describing the evaluation of trial characteristics. Supplementary Table 8. Summary of conditions studied by included reviews. Supplementary Table 9. Summary of quality appraisal using R-AMSTAR. Supplementary Fig. 1. Frequency of R-AMSTAR total and domain scores. Supplementary Table 10. Summary of participant characteristics evaluated by included reviews. Supplementary Table 11. Summary of participant characteristics evaluated among conditions studied. Supplementary Table 12. Summary of trial characteristics evaluated by included reviews. Supplementary Table 13. Summary of trial characteristics evaluated among conditions studied [file 13063_2025_8794_MOESM1_ESM.docx]

## Supplementary Table 1. PRISMA checklist

| **Section and Topic** | **Item #** | **Checklist item** | **Location where item is reported** |
| --- | --- | --- | --- |
| **TITLE** | | |  |
| Title | 1 | Identify the report as a systematic review. | Page 1 |
| **ABSTRACT** | | |  |
| Abstract | 2 | See the PRISMA 2020 for Abstracts checklist. | Page 1-3 |
| **INTRODUCTION** | | |  |
| Rationale | 3 | Describe the rationale for the review in the context of existing knowledge. | Page 3-5 |
| Objectives | 4 | Provide an explicit statement of the objective(s) or question(s) the review addresses. | Page 4-5 |
| **METHODS** | | |  |
| Eligibility criteria | 5 | Specify the inclusion and exclusion criteria for the review and how studies were grouped for the syntheses. | Page 5, Supplementary Table 1 |
| Information sources | 6 | Specify all databases, registers, websites, organisations, reference lists and other sources searched or consulted to identify studies. Specify the date when each source was last searched or consulted. | Page 6 |
| Search strategy | 7 | Present the full search strategies for all databases, registers and websites, including any filters and limits used. | Page 6, Supplementary Table 2 |
| Selection process | 8 | Specify the methods used to decide whether a study met the inclusion criteria of the review, including how many reviewers screened each record and each report retrieved, whether they worked independently, and if applicable, details of automation tools used in the process. | Page 6 |
| Data collection process | 9 | Specify the methods used to collect data from reports, including how many reviewers collected data from each report, whether they worked independently, any processes for obtaining or confirming data from study investigators, and if applicable, details of automation tools used in the process. | Page 6 |
| Data items | 10a | List and define all outcomes for which data were sought. Specify whether all results that were compatible with each outcome domain in each study were sought (e.g. for all measures, time points, analyses), and if not, the methods used to decide which results to collect. | Page 6-7, Table 1, Supplementary Tables 7-8 |
|  | 10b | List and define all other variables for which data were sought (e.g. participant and intervention characteristics, funding sources). Describe any assumptions made about any missing or unclear information. | Page 6-7, Table 1, Supplementary Tables 7-8 |
| Study risk of bias assessment | 11 | Specify the methods used to assess risk of bias in the included studies, including details of the tool(s) used, how many reviewers assessed each study and whether they worked independently, and if applicable, details of automation tools used in the process. | Page 7, Supplementary Table 3 |
| Effect measures | 12 | Specify for each outcome the effect measure(s) (e.g. risk ratio, mean difference) used in the synthesis or presentation of results. | Page 7 |
| Synthesis methods | 13a | Describe the processes used to decide which studies were eligible for each synthesis (e.g. tabulating the study intervention characteristics and comparing against the planned groups for each synthesis (item #5)). | Page 7-8 |
|  | 13b | Describe any methods required to prepare the data for presentation or synthesis, such as handling of missing summary statistics, or data conversions. | Page 7-8 |
|  | 13c | Describe any methods used to tabulate or visually display results of individual studies and syntheses. | Page 7-8 |
|  | 13d | Describe any methods used to synthesize results and provide a rationale for the choice(s). If meta-analysis was performed, describe the model(s), method(s) to identify the presence and extent of statistical heterogeneity, and software package(s) used. | Page 7-8 |
|  | 13e | Describe any methods used to explore possible causes of heterogeneity among study results (e.g. subgroup analysis, meta-regression). | Not reported |
|  | 13f | Describe any sensitivity analyses conducted to assess robustness of the synthesized results. | Not reported |
| Reporting bias assessment | 14 | Describe any methods used to assess risk of bias due to missing results in a synthesis (arising from reporting biases). | Not reported |
| Certainty assessment | 15 | Describe any methods used to assess certainty (or confidence) in the body of evidence for an outcome. | Not reported |
| **RESULTS** | | |  |
| Study selection | 16a | Describe the results of the search and selection process, from the number of records identified in the search to the number of studies included in the review, ideally using a flow diagram. | Page 8 |
|  | 16b | Cite studies that might appear to meet the inclusion criteria, but which were excluded, and explain why they were excluded. | Not reported |
| Study characteristics | 17 | Cite each included study and present its characteristics. | Page 8-9, Table 1, Supplementary Tables 7-8 |
| Risk of bias in studies | 18 | Present assessments of risk of bias for each included study. | Page 9, Supplementary Table 5, Supplementary Figure 1 |
| Results of individual studies | 19 | For all outcomes, present, for each study: (a) summary statistics for each group (where appropriate) and (b) an effect estimate and its precision (e.g. confidence/credible interval), ideally using structured tables or plots. | Page 10-11, Figures 1-2.2, Supplementary Table 6-12 |
| Results of syntheses | 20a | For each synthesis, briefly summarise the characteristics and risk of bias among contributing studies. | Page 8-9 |
|  | 20b | Present results of all statistical syntheses conducted. If meta-analysis was done, present for each the summary estimate and its precision (e.g. confidence/credible interval) and measures of statistical heterogeneity. If comparing groups, describe the direction of the effect. | Not reported |
|  | 20c | Present results of all investigations of possible causes of heterogeneity among study results. | Not reported |
|  | 20d | Present results of all sensitivity analyses conducted to assess the robustness of the synthesized results. | Not reported |
| Reporting biases | 21 | Present assessments of risk of bias due to missing results (arising from reporting biases) for each synthesis assessed. | Not reported |
| Certainty of evidence | 22 | Present assessments of certainty (or confidence) in the body of evidence for each outcome assessed. | Not reported |
| **DISCUSSION** | | |  |
| Discussion | 23a | Provide a general interpretation of the results in the context of other evidence. | Page 12 |
|  | 23b | Discuss any limitations of the evidence included in the review. | Page 13 |
|  | 23c | Discuss any limitations of the review processes used. | Page 13 |
|  | 23d | Discuss implications of the results for practice, policy, and future research. | Page 14-16 |
| **OTHER INFORMATION** | | |  |
| Registration and protocol | 24a | Provide registration information for the review, including register name and registration number, or state that the review was not registered. | Page 3 |
|  | 24b | Indicate where the review protocol can be accessed, or state that a protocol was not prepared. | Page 3 |
|  | 24c | Describe and explain any amendments to information provided at registration or in the protocol. | Page 5 |
| Support | 25 | Describe sources of financial or non-financial support for the review, and the role of the funders or sponsors in the review. | Page 17 |
| Competing interests | 26 | Declare any competing interests of review authors. | Page 17 |
| Availability of data, code and other materials | 27 | Report which of the following are publicly available and where they can be found: template data collection forms; data extracted from included studies; data used for all analyses; analytic code; any other materials used in the review. | Page 17 |

## Supplementary Table 2. Eligibility criteria.

| **PECOS** | **Inclusion criteria** | **Exclusion criteria** |
| --- | --- | --- |
| Population | Adults | Adolescents or children |
| Exposure | Any exposure | No restrictions |
| Characteristics | Any characteristic | No restrictions |
| Outcome | Any outcome describing trial attrition (e.g. Dropout, attrition, trial withdrawal) | Outcomes not describing trial attrition (e.g. treatment dropout but continued participation). |
| Setting | Systematic reviews or meta-analyses derived from systematic reviews of phase III/IV randomised controlled trials or of an unspecified phase | Meta-analysis not embedded within a systematic review, systematic reviews of phase I/II randomised controlled trials, systematic reviews of non-randomised trials |
| Miscellaneous | Published in English, Quantitative literature, Performed quantitative analysis | Published in languages other than English, Qualitative literature, Lacking quantitative data analysis |

## Supplementary Table 3. Search strategy used for each database.

| **Database** | **Search strategy** |
| --- | --- |
| Ovid Medline All (1946 - Present)  N=949 | 1 ((predict* or associ* or factor* or effect* or correlat* or influen*) adj3 (attrition or retention or drop*-out* or dropout* or drop out or ((lost or loss) adj (follow-up or followup)))).ab,ti. 18933  2 exp Patient Dropouts/ 8394  3 1 or 2 26207  4 meta-analysis/ or systematic review/ or systematic reviews as topic/ or meta-analysis as topic/ or "meta analysis (topic)"/ or "systematic review (topic)"/ or exp network meta-analysis/ 329654  5 ((systematic* adj3 (review* or overview*)) or (methodologic* adj3 (review* or overview*))).ti,ab,kf. 306273  6 ((quantitative adj3 (review* or overview* or synthes*)) or (research adj3 (integrati* or overview*))).ti,ab,kf. 15093  7 ((integrative adj3 (review* or overview*)) or (collaborative adj3 (review* or overview*)) or (pool* adj3 analy*)).ti,ab,kf. 37673  8 (data synthes* or data extraction* or data abstraction*).ti,ab,kf. 38908  9 (handsearch* or hand search*).ti,ab,kf. 10953  10 (mantel haenszel or peto or der simonian or dersimonian or fixed effect* or latin square*).ti,ab,kf. 34663  11 (met analy* or metanaly*).ti,ab,kf. 835  12 (meta regression* or metaregression*).ti,ab,kf. 14000  13 (meta-analy* or metaanaly* or systematic review*).mp,hw. 450551  14 (multi* adj2 paramet* adj2 evidence adj2 synthesis).ti,ab,kf. 14  15 (multiparamet* adj2 evidence adj2 synthesis).ti,ab,kf. 18  16 (multi-paramet* adj2 evidence adj2 synthesis).ti,ab,kf. 12  17 or/4-16 525178  18 3 and 17 1138  19 remove duplicates from 18 1115  20 limit 19 to english language 1106  21 exp humans/ not animals.mp. [mp=title, book title, abstract, original title, name of substance word, subject heading word, floating sub-heading word, keyword heading word, organism supplementary concept word, protocol supplementary concept word, rare disease supplementary concept word, unique identifier, synonyms, population supplementary concept word, anatomy supplementary concept word] 18900737  22 20 and 21 949 |
| Ovid Embase (1947 - Present)  N=1089 | 1 ((predict* or associ* or factor* or effect* or correlat* or influen*) adj3 (attrition or retention or drop*-out* or dropout* or drop out or ((lost or loss) adj (follow-up or followup)))).ab,ti. 25213  2 exp patient dropout/ 2012  3 1 or 2 26837  4 meta-analysis/ or systematic review/ or systematic reviews as topic/ or meta-analysis as topic/ or "meta analysis (topic)"/ or "systematic review (topic)"/ or exp network meta-analysis/ 606902  5 ((systematic* adj3 (review* or overview*)) or (methodologic* adj3 (review* or overview*))).ti,ab,kf. 390825  6 ((quantitative adj3 (review* or overview* or synthes*)) or (research adj3 (integrati* or overview*))).ti,ab,kf. 18104  7 ((integrative adj3 (review* or overview*)) or (collaborative adj3 (review* or overview*)) or (pool* adj3 analy*)).ti,ab,kf. 54940  8 (data synthes* or data extraction* or data abstraction*).ti,ab,kf. 49425 9 (handsearch* or hand search*).ti,ab,kf. 13581  10 (mantel haenszel or peto or der simonian or dersimonian or fixed effect* or latin square*).ti,ab,kf. 46983  11 (met analy* or metanaly*).ti,ab,kf. 1812  12 (meta regression* or metaregression*).ti,ab,kf. 18015  13 (meta-analy* or metaanaly* or systematic review*).mp,hw. 719406 14 (multi* adj2 paramet* adj2 evidence adj2 synthesis).ti,ab,kf. 28  15 (multiparamet* adj2 evidence adj2 synthesis).ti,ab,kf. 21  16 (multi-paramet* adj2 evidence adj2 synthesis).ti,ab,kf. 23  17 or/4-16 815092  18 3 and 17 1222  19 remove duplicates from 18 1163  20 limit 19 to english language 1151  21 exp humans/ not animals.mp. [mp=title, abstract, heading word, drug trade name, original title, device manufacturer, drug manufacturer, device trade name, keyword heading word, floating subheading word, candidate term word] 26327449  22 20 and 21 1089 |
| Web of Science Core Collection (1992 - Present)  N=932 | 1 TI=((predict* or associ* or factor* or effect* or correlat* or influen*) NEAR/3 (attrition or drop*out* or dropout* or ((loss or lost) NEAR (follow-up or followup)))) or AB=((predict* or associ* or factor* or effect* or correlat* or influen*) NEAR/3 (attrition or drop*-out* or dropout* or ((loss or lost) NEAR (follow-up or followup)))) 10163  2 TI=(((systematic* NEAR/3 (review* or overview*)) or (methodologic* NEAR/3 (review* or overview*)))) or AB=(((systematic* NEAR/3 (review* or overview*)) or (methodologic* NEAR/3 (review* or overview*)))) 364340  3 TI=((quantitative NEAR/3 (review* or overview* or synthes*)) or (research NEAR/3 (integrati* or overview*))) or AB=((quantitative NEAR/3 (review* or overview* or synthes*)) or (research NEAR/3 (integrati* or overview*))) 43057  4 TI=(((integrative NEAR/3 (review* or overview*)) or (collaborative NEAR/3 (review* or overview*)) or (pool* NEAR/3 analy*))) or AB=(((integrative NEAR/3 (review* or overview*)) or (collaborative NEAR/3 (review* or overview*)) or (pool* NEAR/3 analy*))) 51685  5 TI=(("data synthes*" or "data extraction*" or "data abstraction*" or handsearch or "hand search*")) or AB=(("data synthes*" or "data extraction*" or "data abstraction*" or handsearch or "hand search*")) 46358  6 TI=(("met analy*" or metanaly* or "meta regression*" or metaregression*)) or AB=(("met analy*" or metanaly* or "meta regression*" or metaregression*)) 15677  7 TS=("meta-analy*" or metaanaly* or "systematic review*") 671232  8 TI=(multi* NEAR/2 paramet* NEAR/2 evidence NEAR/2 synthesis) or AB=(multi* NEAR/2 paramet* NEAR/2 evidence NEAR/2 synthesis) 15 9 TI=(multiparamet* NEAR/2 evidence NEAR/2 synthesis) or AB=(multiparamet* NEAR/2 evidence NEAR/2 synthesis) 15  10 #2 OR #3 OR #4 OR #5 OR #6 OR #7 OR #8 OR #9 798049  11 #1 AND #10 932 |
| ORCCA (1986 - 2019)  N=158 | 1 Retention Research Methods = Systematic review and reviews 158 |

## Supplementary Table 4. R-AMSTAR checklist. Total score out of 44, with a maximum of 4 points allocated to each domain based on the criteria satisfied.

| **Domain** | **Criteria** | **Scoring criteria** |
| --- | --- | --- |
| 1: Was an ‘‘a priori’’ design provided? | A: A clearly focused (PICO-based) question  B: Description of inclusion criteria  C: Study protocol is published and/or registered in advance | 4: Three criteria  3: Two criteria  2: One criterion  1: No criteria satisfied |
| 2: Was there duplicate study selection and data extraction? | A: At least two persons independently extracted the data, explicitly stated  B: Statement of consensus procedure for disagreements  C: Disagreements among extractors resolved properly as stated or implied | 4: Three criteria  3: Two criteria  2: One criterion  1: No criteria satisfied |
| 3: Was a comprehensive literature search performed? | A: At least two electronic sources are searched  B: Years and databases used are mentioned  C: Keywords and/or MESH terms are stated and where feasible the search strategy outline is provided  D: Searches should are supplemented by consulting current contents, reviews, textbooks, registers and by reviewing the references in the studies found  E: Journals are hand-searched or manual searched | 4: Five/Four criteria  3: Three criteria  2: Two criteria  1: One/No criteria satisfied |
| 4: Was the status of publication (i.e., grey literature) used as an inclusion criterion? | A: The authors state that they searched for reports regardless of their publication type  B: The authors state whether or not they excluded any reports based on their publication status, language etc  C: “non-English papers were translated” or readers sufficiently trained in foreign language  D: No language restriction or recognition of non-English articles | 4: Four/Three criteria  3: Two criteria  2: One criterion  1: No criteria satisfied |
| 5: Was a list of studies (included and excluded) provided? | A: Table/list/figure of included studies, a reference list does not suffice  B: Table/list/figure of excluded studies either in the article or in a supplemental source  C: Satisfactory/sufficient statement of the reason for exclusion of the seriously considered studies  D: Reader is able to retrace the included and the excluded studies anywhere in the article bibliography, reference or supplemental source | 4: Four criteria  3: Three criteria  2: Two criteria  1: One criterion |
| 6: Were the characteristics of the included studies provided? | A: In an aggregated form such as a table, data from the original studies are provided on the participants, interventions/exposure and outcomes  B: Ranges are provided of the relevant characteristics in the studies analysed  C: The information provided appears to be complete and accurate | 4: Three criteria  3: Two criteria  2: One criterion  1: No criteria satisfied |
| 7: Was the scientific quality of the included studies assessed and documented? | A: ‘A priori’ methods are provided  B: The scientific quality of the included studies appears to be meaningful  C: Discussion/recognition/awareness of level of evidence is present  D: Quality of evidence is rated/ranked base on characterised instruments | 4: Four criteria  3: Three criteria  2: Two criteria  1: One/No criteria satisfied |
| 8: Was the scientific quality of the included studies used appropriately in formulating conclusions? | A: The scientific quality is considered in the analysis and the conclusions of the review  B: The scientific quality is explicitly stated in formulating recommendations  C: Conclusions integrated/drives towards practice guidelines  D: Clinical consensus statement drives toward revision or consensus | 4: Four criteria  3: Three criteria  2: Two criteria  1: One/No criteria satisfied |
| 9: Were the methods used to combine the findings of studies appropriate? | A: Statement of criteria that were used to decide that the studies analysed were similar enough to be pooled  B: For the pooled results, a test is done to ensure the studies were combinable, to assess their homogeneity  C: Recognition of heterogeneity or lack  thereof is present  D: If heterogeneity exists a ‘random effects model’ is used and/or the rationale of combining is taken into consideration  E: If homogeneity exists, author state a rationale or a statistical test | 4: Five/Four criteria  3: Three criteria  2: Two criteria  1: One/No criteria satisfied |
| 10: Was the likelihood of publication bias assessed? | A: Recognition of publication bias or file drawer effect  B: Graphical aids (e.g. funnel plot)  C: Statistical tests (e.g. Egger regression test) | 4: Three criteria  3: Two criteria  2: One criterion  1: No criteria satisfied |
| 11: Was the conflict of interest included? | A: Statement of sources of support  B: No conflict of interest. This is subjective and may require some deduction or searching  C: An awareness/statement of support or conflict of interest in the primary inclusion studies | 4: Three criteria  3: Two criteria  2: One criterion  1: No criteria satisfied |

## Supplementary Table 5. Additional review characteristics describing attrition outcomes and definitions

| **Review** | **Attrition outcome** | **Definition** |
| --- | --- | --- |
| Albano et al. (2019) | End of treatment dropout | Not defined |
| Aparicio et al. (2016) | Acceptability | Rates of treatment discontinuation for all causes |
| Arafah et al. (2017) | Attrition or dropout rate | Proportion of participants who dropped out from the study (at the first evaluation point post-intervention) out of the number of participants who consented to participate in the study |
| Bacaltchuk et al. (2001) | Acceptability (dropout due to any cause) | Not defined |
|  | Tolerability (dropout due to adverse events) | Not defined |
| Benbow et al. (2019) | Attrition | Number of participants randomised to a virtual reality exposure therapy (VRET) or in vivo exposure treatment condition who did not complete the number of treatment sessions specified by the trial |
| Bevens et al. (2022) | Attrition | Not defined |
| Bighelli et al. (2018) | All-cause discontinuation | Study dropout for any reason |
| Bricca et al. (2022) | Retention rate | Number providing biochemically verified outcomes at any or follow-up assessment/number randomised |
|  | Differential retention rate | Difference in number providing biochemically verified outcomes in the intervention group and number providing biochemically verified outcomes in the comparator group/number randomised |
| Chen et al. (2021) | Dropout rate | The percentage of patients dropping out for any reason before study completion |
| Cooper et al. (2015) | Dropout rate | Unexpected participant attrition among individuals who were randomised to a treatment but failed to complete it |
| Cramer et al. (2016) | Dropout rate | Not defined |
| Crutzen et al. (2015) | Relative attrition | Not defined |
| Cuijpers et al. (2009) | Dropout | Not defined |
| Cunill et al. (2013) | All-cause treatment discontinuation | The proportion of patients randomised who did not complete the study for any reason |
|  | Proportion of patients who discontinued because of lack of efficacy | Not defined |
|  | Proportion of patients who discontinued due to adverse events | Not defined |
| De Campos Moreira et al. (2017) | Dropout rate | Not defined |
| DeCrescenzo et al. (2018) | Acceptability (dropout due to any cause) | Proportion of participants who dropped out from the study due to any cause |
| Dixon et al. (2020) | Dropout | Number of participants who dropped out of DBT defined by either |
|  | Treatment dropout | When participants dropped out anytime during the course of DBT regardless of what session they were up to |
|  | Study dropout | When participants failed to complete post-test/follow-up assessments, or when unspecified that participants dropped out during treatment |
| Doyle et al. (2021) | Acceptability | The proportion of patients who discontinue treatment for any reason |
| Dudas et al. (2018) | Tolerability (withdrawal from trial) | Not defined |
| Edwards-Stewart et al. (2021) | Primary and secondary treatment dropout | Total number of participants who stopped attending treatment sessions |
|  | Waitlist dropout | Participants who withdrew from the study during the treatment phase |
| Elsner et al. (2020) | Dropouts | Not defined |
| Fabricatore et al. (2009) | Total attrition from all causes | The number of dropouts at 1 year after randomisation divided by the number of randomised participants |
|  | AE-related attrition | Not defined |
|  | Non-AE-related attrition | Not defined |
| Frampton et al. (2003) | Safety (incidence of adverse events leading to withdrawal) | Not defined |
|  | Acceptability (withdrawal from trials) | Not defined |
| Furukawa et al. (2020) | Dropout due to AE | Not defined |
|  | Dropout for any reason | Not defined |
| Gagliardi et al. (2019) | Dropouts | Not defined |
| Gehling et al. (2011) | Dropout due to lack of effect | Not defined |
|  | Dropout due to adverse events | Not defined |
|  | Total dropout | Not defined |
| Goldberg et al. (2021) | Differential attrition | Not defined |
| Harris et al. (2021) | Retention rate | Proportion of randomised people (proportion of people providing the outcomes of interest/proportion randomised) providing physical (i.e. physical function) and/or psychosocial outcomes (i.e. HRQoL, depression symptoms and anxiety symptoms) at the end of the intervention and the follow-up closest to 12 months |
|  | Differential retention rate | Difference in proportion of people providing physical (i.e. physical function) and/or psychosocial outcomes (i.e. HRQoL, depression symptoms and anxiety symptoms) in the intervention and comparator group, at the end of the intervention and the follow-up closest to 12 months |
| Heneghan et al. (2007) | Differential attrition | Not defined |
| Heo et al. (2009) | Attrition size | Number of dropouts during the study and number of subjects who were randomised but excluded from study-specific efficacy analysis |
|  |  |  |
| Hernandez-Rodriguez et al. (2022) | Dropout rate | When a participant did not complete the intervention or follow-up period after the randomisation process |
| Hornyak et al. (2014) | Safety (dropouts due to adverse events) | Not defined |
| Hrobjartsson et al. (2014) | Number of dropouts | Number of patients unavailable for assessment |
| Huang et al. (2014) | Acceptability | Total dropout, dropout due to inefficacy, dropout due to side effects |
| Ibrahim et al. (2016) | Dropout rate | Not defined |
| Iliakis et al. (2021) | Dropout rate | Proportion of those starting treatment that did not complete treatment |
| Imel et al. (2013) | Dropout | Number of individuals randomised to a treatment condition that did not complete the full course, regardless of reason |
| Jabardo-Camprubi et al. (2020) | Dropout odds ratio | Not defined |
| Karyotaki et al. (2015) | Treatment dropout | Not defined |
| Kato et al. (2021) | Acceptability | All-cause dropout |
|  | Tolerability | Dropout due to adverse events |
| Kline et al. (2021) | Dropout rate | Patients who were randomised but failed to complete treatment as prescribed, except those who were removed administratively |
| Koog et al. (2013) | Attrition reasons | Not defined |
|  | Discontinuation from trial | Not defined |
| Kredo et al. (2013) | Attrition | Composite of loss to follow-up or death |
| Lam et al. (2022) | Attrition rate | Ratio between dropout and intention to treat sample sizes |
| Leucht et al. (2017) | Dropout rate | Discontinuation related to any cause and inefficacy |
| Levinson et al. (2022) | Dropout rate | Number of participants who discontinued prolonged exposure (PE) without meeting the study's a priori conditions for PE completion |
| Lewis et al. (2020) | Dropout | The number of participants that left the study at the point of post-treatment assessment |
| Li et al. (2020) | Placebo dropout rate | Not defined |
| Linardon et al. (2018) | Dropout rate | The number of participants who started CBT (or comparison condition) and who were then identified as dropouts according to the author's definition of dropout. Includes any individual who would be included in the ITT population (e.g. those who never attended a session). |
| Linardon et al. (2019) | Dropout | Number of patients who started interpersonal therapy (IPT) and who were then identified as a dropout according to study author definitions, thus included in the intention-to-treat analysis (e.g. those who stopped attending sessions or failed to provide post-treatment data) |
| Linardon et al. (2020) | Study attrition | The number of participants randomised and who then did not complete the research protocol, which included failure to complete the follow-up assessments |
| Makatsori et al. (2014) | Dropout | Any situation causing premature withdrawal from the trial after randomisation and before the stated completion of the trial |
| Martin et al. (2006) | All-cause discontinuation of treatment | Not defined |
|  | Specific discontinuation due to adverse events | Not defined |
| Matsusaki et al. (2019) | Dropout owing to lack of efficacy | Not defined |
|  | Dropout owing to adverse events | Not defined |
| McVay et al. (2023) | Trial retention | Not defined |
| Minozzi et al. (2020) | Dropout from treatment | Number of women who had dropped out at the end of the intervention |
| Mitsikostas et al. (2012) | Dropout due to adverse event | Not defined |
|  | Nocebo dropout | Pooled percentage of placebo-treated patients who discontinued because of intolerance |
| Miyasaka et al. (2006) | Number of participants dropping out due to side effects | Not defined |
|  | Total number of dropouts | Not defined |
| Ong et al. (2016) | Dropout | Attrition following the start of treatment |
| Ong et al. (2018) | Dropout | Attrition following the start of therapy |
| Palmowski et al. (2020) | Participant retention | Not defined |
|  | Attrition due to adverse events | Not defined |
|  | Attrition due to lack of efficacy | Not defined |
| Pampallona et al. (2004) | Dropout for any reason | Not defined |
| Papadopoulos et al. (2010) | Dropout due to drug-related adverse reactions (DO) | Not defined |
|  | Withdrawal from trial | Not defined |
| Pozza et al. (2017) | Dropout rate | Number of participants who did not complete all the scheduled treatment sessions |
| Rabinowitz et al. (2009) | Total treatment arm dropout | Not defined |
| Reas et al. (2008) | Attrition | Withdrawal or noncompletion for any reason |
| Rehman et al. (2021) | All cause dropout | Not defined |
|  | Dropout due to AE | Not defined |
| Rutherford et al. (2013) | Dropout | Not defined |
| Schalkwijk et al. (2014) | Total dropout | Not defined |
|  | Dropout ascribed to lack of efficacy | Not defined |
|  | Dropout ascribed to adverse events | Not defined |
|  | Dropout ascribed to miscellaneous factors | Not defined |
| Shah et al. (2020) | Dropout attrition | Not defined |
| Somerson et al. (2016) | Loss to follow-up | Number of subjects failing to be evaluated for the primary study endpoint at the predetermined minimum follow-up period |
| Song et al. (2021) | Retention rate | Not defined |
| Stahl et al. (1993) | Overall dropout | Not defined |
|  | Dropouts due to adverse events | Not defined |
|  | Dropouts due to perceived lack of drug effect | Not defined |
| Stubbs et al. (2016) | Dropout rate | Unexpected participant attrition among individuals who were randomised to a treatment but failed to complete it |
| Swift et al. (2017) | Premature termination | Attending at least one session but failing to complete the treatment as defined by study authors |
| Szymczynska et al. (2017) | Dropout from experimental intervention | Proportion of participants reported as not completing the intervention according to author's definition |
|  | Dropout from study | Proportion of participants who did not complete the last follow-up assessment in all study arms |
| Tedeschini et al. (2010) | Discontinuation rate | Per each protocol |
| Torous et al. (2020) | Dropout rate | Number of participants in each group who completed end-of-intervention assessments divided by total number randomised to that condition for each arm of study |
| Vancampfort et al. (2016) | Treatment dropout rate | Unexpected patient attrition among individuals who were randomised to a treatment but failed to complete it |
| Vancampfort et al. (2017) | Treatment dropout rate | Unexpected patient attrition among individuals who were randomised to a treatment but failed to complete it until the end of the study |
| Vancampfort et al. (2021) | Treatment dropout rate | Unexpected patient attrition among individuals who were randomised to a treatment but failed to complete it |
| Villeneuve et al. (2010) | Dropout | Not defined |
| Wahlbeck et al. (2001) | Number of dropouts | The number of patients leaving the study preterm due to any reason |
| Wasmann et al. (2019) | Loss to follow-up | Not defined |
| Weinmann et al. (2008) | Overall dropout rate, dropouts due to adverse events) | Not defined |
|  | Dropouts due to adverse events | Not defined |
| Windle et al. (2020) | Dropout rate | Not defined |
| Zhang et al. (2021) | Rate of premature discontinuation | Not defined |
| Zhou et al. (2020) | Dropout | Those who did not complete intervention (used if author did not state meaning) |

## Supplementary Table 6. Additional review characteristics describing the evaluation of participant characteristics.

| **Review** | **Methods** | **Associated participant characteristic** | **Descriptive findings** | **Measures of effect/significance** | **Unassociated participant characteristics** |
| --- | --- | --- | --- | --- | --- |
| Albano et al. (2019) | Random effects model | Not analysed | Not analysed | Not analysed | Not analysed |
| Aparicio et al. (2016) | Random effects model, Mantel-Haenszel method, Meta regression, ANOVA, Univariate linear regressions | None | None | None | Age, sex |
| Arafah et al. (2017) | Random effects model, Univariate and multivariate meta regression | Not analysed | Not analysed | Not analysed | Not analysed |
| Bacaltchuk et al. (2001) | Random effects model, Sensitivity analyses | Not analysed | Not analysed | Not analysed | Not analysed |
| Benbow et al. (2019) | Random effects model, Mantel-Haenszel method, Meta regression, Subgroup analyses | Not analysed | Not analysed | Not analysed | Not analysed |
| Bevens et al. (2022) | Restricted maximum likelihood method, Univariate and multivariate regression | Not analysed | Not analysed | Not analysed | Not analysed |
| Bighelli et al. (2018) | Random effects model, Frequentist network meta-analysis | Not analysed | Not analysed | Not analysed | Not analysed |
| Bricca et al. (2022) | Mixed effects model, Meta regression | Ongoing physical condition | Having an ongoing physical condition was associated with higher retention (lower attrition) | OR [95% CI]; p-value - 1.66 [1.04, 2.63]; p=0.03 | Age, socioeconomic status, nicotine dependency, ongoing mental health condition, health triggers |
|  |  | Sex | Having a greater % of female participants was associated with lower retention (higher attrition) | OR [95% CI]; p-value - 0.86 [0.98, 0.71]; p=0.03 |  |
|  |  | Motivation to quit | Participants being motivated to quit smoking was associated with lower retention (higher attrition | OR [95% CI]; p-value - 0.74 [0.99, 0.54]; p=0.04 |  |
| Chen et al. (2021) | Frequentist network meta-analysis, Mixed effects model, Generalised linear regression, Subgroup analyses | Not analysed | Not analysed | Not analysed | Not analysed |
| Cooper et al. (2015) | Random effects model, Mixed effects model, Meta regression, ANOVA-like tests | Race | Studies with a greater % of racially diverse samples had significantly higher dropout than studies | Slope [95% CI]; Intercept [95% CI]; p-value - 0.432 [0.043, 0.822]; 0.7843 [0.668, 0.900]; p=0.03 | Sex, marital status, age, taking antidepressants |
|  |  | Personality disorders | Participants diagnosed with personality disorders were significantly more likely to dropout | Slope [95% CI]; Intercept [95% CI]; p-value - 0.976 [0.563, 1.388]; 0.533 [0.351, 0.716]; p=0.00 |  |
| Cramer et al. (2016) | Random effects model, Subgroup analyses, Chi-square | Health status | Healthy participants had significantly lower dropout than participants with a medical condition | Dropout rate [95% CI]; Chi-sq; p-value; N - 9.34 [7.16, 11.51]; 5.09; p=0.02 | None |
|  |  | Medical conditions | Dropout differed significantly between conditions, and was notably highest in HIV participants | Dropout rate [95% CI]; Chi-sq; p-value; N - 22.20 [4.30, 40.09]; 47.67; p<0.00001 |  |
|  |  | Sex | Female participants were significantly more likely to dropout than male participants | Dropout rate [95% CI]; Chi-sq; p-value; N - 14.19 [11.36, 17.02]; 25.44; p<0.00001 |  |
|  |  | Age | Studies with mixed aged participants had significantly higher dropout | Dropout rate [95% CI]; Chi-sq; p-value; N - 26.61 [11.37, 41.85]; 17.74; p=0.001 |  |
| Crutzen et al. (2015) | Random effects model, Mixed effects model, Univariate and multivariate meta regression | Not analysed | Not analysed | Not analysed | Not analysed |
| Cuijpers et al. (2009) | Random effects model, Mixed effects model, Meta regression, Subgroup analyses | None | None | None | Diagnosis, depression severity |
| Cunill et al. (2013) | Random effects model, Meta regression, Fixed effects model, Sensitivity analysis | Not analysed | Not analysed | Not analysed | Not analysed |
| De Campos Moreira et al. (2017) | Random effects model, Meta regression | Health status | Healthy participants were more likely to dropout than unhealthy participants | Propotion [95% CI]; p-value - 0.45 [0.27, 0.78]; p=0.03 | None |
| DeCrescenzo et al. (2018) | Random effects model, Network meta-analysis, Subgroup network meta-analysis | None | None | None | Sex, age, use of opioid therapy, substance use, comorbid alcohol abuse |
| Dixon et al. (2020) | Random effects model, Borenstein et al. method, Mixed effects model, Subgroup analyses | Not analysed | Not analysed | Not analysed | Not analysed |
| Doyle et al. (2021) | Random effects model, Frequentist network meta-analysis, Subgroup analyses | Not analysed | Not analysed | Not analysed | Not analysed |
| Dudas et al. (2018) | Fixed effects, Mantel-Haenszel method | Not analysed | Not analysed | Not analysed | Not analysed |
| Edwards-Stewart et al. (2021) | Random effects model, Unstructured correlation matrix, Subset analysis | Not analysed | Not analysed | Not analysed | Not analysed |
| Elsner et al. (2020) | Random effects model, Mantel-Haenszel method | Not analysed | Not analysed | Not analysed | Not analysed |
| Fabricatore et al. (2009) | Mixed effects model | Sex | Studies with a greater % of women had significantly higher total attrition | Coefficient (SD); p-value - 0.33 (13); p=0.02 | Age |
|  |  |  |  | F-statistic; p-value - 9.92; p<0.01 |  |
|  |  |  | Studies with a greater % of women had significantly higher non-AE-related attrition | Coefficient (SD); p-value - 0.37 (0.13); p=0.01 |  |
|  |  |  |  | F-statistic; p-value - 9.49; p<0.01 |  |
|  |  | BMI | Participants having a high BMI was significantly associated with lower AE-related attrition | Coefficient (SD); p-value - -0.86 (0.40); p=0.04 |  |
|  |  |  | Participants having a high BMI was significantly associated with higher non-AE-related attrition | Coefficient (SD); p-value - 2.51 (1.19); p=0.05 |  |
| Frampton et al. (2003) | Peto odds ratio | Not analysed | Not analysed | Not analysed | Not analysed |
| Furukawa et al. (2020) | Random effects model | Not analysed | Not analysed | Not analysed | Not analysed |
| Gagliardi et al. (2019) | Random effects model, Subgroup analyses, Sensitivity analyses | Not analysed | Not analysed | Not analysed | Not analysed |
| Gehling et al. (2011) | Random effects model, Fixed effects model | Not analysed | Not analysed | Not analysed | Not analysed |
| Goldberg et al. (2021) | Random effects model, Meta regression | Not analysed | Not analysed | Not analysed | Not analysed |
| Harris et al. (2021) | Random effects model, Meta regression, Subgroup analyses | Age, Hypertension | Having a higher proportion of participants with hypertension and older age was associated with lower retention | Coefficient [95% CI]; Tau-sq; N - -0.01 [-0.01, -0.01]; 0.06; 10 | Sex, BMI, socioeconomic status, baseline condition severity, number of comorbidities, severity of comorbidities, physical function, HRQoL, depression and anxiety |
| Heneghan et al. (2007) | DerSimonian-Laird random effects model, Meta regression | Age | Older aged participants were significantly more likely to dropout from trials involving self-monitoring of oral anticoagulation drugs | p-value; N - p=0.012; 1 | None |
| Heo et al. (2009) | Mantel-Haenszel method, Univariate and multivariate logistic regression, Forward stepwise correlation analysis | Depression indication | Participants with a greater indication of depression had a significantly greater odds of dropping out | Univariate OR [95% CI]; p-value - 1.04 [1.02, 1.05]; p<0.0001 | Sex, age |
|  |  |  | Participants with a greater indication of depression had a significantly greater odds of dropping out | Multivariate OR [95% CI]; p-value - 1.05 [1.03, 1.07]; p<0.0001 |  |
| Hernandez-Rodriguez et al. (2022) | Random effects model, Meta regression, Sensitivity analyses, Subgroup analyses | None | None | None | Age, sex |
| Hornyak et al. (2014) | Random effects model, Mantel-Haenszel | Not analysed | Not analysed | Not analysed | Not analysed |
| Hrobjartsson et al. (2014) | Random effects model | Not analysed | Not analysed | Not analysed | Not analysed |
| Huang et al. (2014) | Random effects model, Fixed effects model, Sensitivity analyses | Not analysed | Not analysed | Not analysed | Not analysed |
| Ibrahim et al. (2016) | Direct comparison, Chi-square, Fisher's exact test | Not analysed | Not analysed | Not analysed | Not analysed |
| Iliakis et al. (2021) | Random effects model, Meta regression | Not analysed | Not analysed | Not analysed | Not analysed |
| Imel et al. (2013) | Random effects model, Meta regression, Sensitivity analyses | Not analysed | Not analysed | Not analysed | Not analysed |
| Jabardo-Camprubi et al. (2020) | Random effects model, Subgroup analyses | None | None | None | Pathology, age, sex |
| Karyotaki et al. (2015) | Bivariate and multivariate Poisson regression, Sensitivity analyses | Sex | Male participants had a significantly higher risk of dropout | Risk ratio [95% CI]; p-value - 1.08 [1.03, 1.13]; p=0.002 | Employment status |
|  |  | Age | Older aged participants had a significantly lower risk of dropout | Risk ratio [95% CI]; p-value - 0.98 [0.97, 0.99]; p=0.004 |  |
|  |  | Education | Less educated participants had a significantly higher risk of dropout | Risk ratio [95% CI]; p-value - 1.26 [1.14, 1.39]; p=0.000 |  |
|  |  | Relationship status | Participants in a relationship had a significantly higher risk of dropout | Risk ratio [95% CI]; p-value - 1.09 [0.95, 1.25]; p=0.05 |  |
|  |  | Comorbid anxiety | Participants with comorbid anxiety had a significantly lower risk of dropout | Risk ratio [95% CI]; p-value - 1.18 [1.01, 1.38]; p=0.03 |  |
|  |  | Baseline depression severity | Participants with a greater severity of baseline depression had a significantly higher risk of dropout | Risk ratio [95% CI]; p-value - 1.004 [1.003, 1.005]; p<0.001 |  |
| Kato et al. (2021) | Random effects model, Subgroup analyses, Meta regression | Age | Older participants of placebo groups were significantly more likely to dropout than active group participants | OR [95% CI]; p-value; N - 0.32 [0.15, 0.71]; p=0.05; 539 | Sex |
| Kline et al. (2021) | Random effects model | None | None | None | PTSD symptoms, condition-specific baseline depression symptoms |
| Koog et al. (2013) | Random effects model, Meta regression | Not analysed | Not analysed | Not analysed | Not analysed |
| Kredo et al. (2013) | Random effects model, Subgroup analyses | Not analysed | Not analysed | Not analysed | Not analysed |
| Lam et al. (2022) | Random effects model, Peto method, Mantel-Haenszel method, Sensitivity analyses, Subgroup analyses, Meta regression | Age | Older age was associated with a greater likelihood of overall attrition in control arms | Coefficient; p-value - -0.002; p=0.005 | Race, sex, marital status, education, medical condition, psychiatric disorder, veteran status |
| Leucht et al. (2017) | Random effects model, Bayesian hierarchical framework | Not analysed | Not analysed | Not analysed | Not analysed |
| Levinson et al. (2022) | Random effects model, Univariate and multivariate regression, Sensitivity analysis | Baseline PTSD symptoms | Greater PTSD symptoms associated with higher dropout | OR [95% CI]; p-value - 1.04 [1.01, 1.07]; p<0.05 | Age, sex, military history |
| Lewis et al. (2020) | Random effects model, Meta regression | None | None | None | Veteran or military status, sexual trauma, sex, education |
| Li et al. (2020) | Random effects model, Univariate and multivariate regression, Subgroup analyses | Baseline illness severity | Participants having less severe baseline illness was associated with higher dropout | Coefficient [95% CI]; p-value - -0.07 [-0.13, -0.02]; p<0.05 | Age, sex |
| Linardon et al. (2018) | Mixed effects model, Meta regression, Subgroup analyses | None | None | None | Diagnosis, age, eating disorder status, self-concerns about disorder, dietary constraint, binge eating frequency, comorbid depression |
| Linardon et al. (2019) | Random effects model, Borenstein et al. method, Mixed effects model, Meta regression, Subgroup analyses | Age | Younger age was associated with lower dropout than older adults who were not elderly | p-value - p=0.006 | Sex, race, relationship status |
| Linardon et al. (2020) | Random effects model, Mixed effects model, Subgroup analyses | Not analysed | Not analysed | Not analysed | Not analysed |
| Makatsori et al. (2014) | Random effects model, Subgroup analyses | None | None | None | Allergic disease, allergens, age |
| Martin et al. (2006) | Random effects model, Fixed effects model, Sensitivity analyses | Not analysed | Not analysed | Not analysed | Not analysed |
| Matsusaki et al. (2019) | Random effects model, Univariate and multivariate meta regression | Age | Older age was associated with higher dropout | Coefficient (SE); p-value - 0.016 (0.008); p=0.044 | Diagnosis, disease severity, sex |
| McVay et al. (2023) | Random effects model, meta regression | Number of pre-enrolment steps | Participants having a greater number of pre-enrolment steps was significantly associated with greater retention (lower attrition) | Multivariate estimate [95% CI]; p-value - 2 [0.2, 3.8]; p=0.028 | None |
|  |  |  |  | Univariate estimate [95% CI]; p-value - 3.1 [0.6, 5.8]; p=0.018 |  |
| Minozzi et al. (2020) | Random effects model | Not analysed | Not analysed | Not analysed | Not analysed |
| Mitsikostas et al. (2012) | Random effects model, Mixed effects model, Meta regression | Comorbidity with depression | Participants having a comorbidity with depression were significantly less likely to dropout from placebo treatment | p-value - p=0.0270 | Sex, race |
|  |  | Age | Older participants were significantly less likely to dropout in placebo treatment | p-value - p=0.0219 |  |
| Miyasaka et al. (2006) | Fixed effects model | Not analysed | Not analysed | Not analysed | Not analysed |
| Ong et al. (2016) | Random effects model, Meta regression, ANOVA | None | None | None | Condition, treatment experience |
| Ong et al. (2018) | Random effects model, Meta regression | None | None | None | Diagnosis, age |
| Palmowski et al. (2020) | Random effects model, Mixed effects model, Meta regression, Sensitivity analyses | Life stage | Studies having a greater % of elderly participants was associated with greater retention (lower attrition) in pharmacological trials | Univariate coefficient [95% CI]; p-value - 0.01 [0.00, 0.02]; p=0.05 | Condition, sex |
|  |  |  |  | Multivariate coefficient [95% CI]; p-value - -0.01 [-0.02, -0.00]; p=0.05 |  |
| Pampallona et al. (2004) | Random effects model, Sensitivity analysis, Meta regression | Not analysed | Not analysed | Not analysed | Not analysed |
| Papadopoulos et al. (2010) | Random effects model, Meta regression | Duration of illness | Participants with a longer disease duration were significantly more likely to dropout due to drug-related adverse reactions | Coefficient; p-value - 0.0057; p=0.525 | Sex |
|  |  | Age | Older participants were significantly more likely to dropout due to drug-related adverse reactions | Coefficient; p-value - 0.0372; 0.0372 |  |
| Pozza et al. (2017) | Random effects model | Not analysed | Not analysed | Not analysed | Not analysed |
| Rabinowitz et al. (2009) | Mixed effects model, Meta regression, Subgroup analyses | Not analysed | Not analysed | Not analysed | Not analysed |
| Reas et al. (2008) | Fixed effects model, Random effects model | Not analysed | Not analysed | Not analysed | Not analysed |
| Rehman et al. (2021) | Random effects model | Not analysed | Not analysed | Not analysed | Not analysed |
| Rutherford et al. (2013) | Mixed effects model, Logistic regression | Not analysed | Not analysed | Not analysed | Not analysed |
| Schalkwijk et al. (2014) | Random effects model, Linear regression, Meta regression | Not analysed | Not analysed | Not analysed | Not analysed |
| Shah et al. (2020) | Random effects model, Mantel-Haenszel method, Two-tailed test, Sensitivity analysis | Not analysed | Not analysed | Not analysed | Not analysed |
| Somerson et al. (2016) | Univariate comparisons, Independence testing, Correlation testing, Linear regression | None | None | None | Age |
| Song et al. (2021) | Quasi-poisson regression | Patient-caregiver relationship | Studies targeting different types of caregivers had significantly lower retention (higher attrition) compared to targeting patient-spousal caregivers | Coefficient (SE); p-value - -0.37 (0.07); p=<0.001 | Cancer stage |
| Stahl et al. (1993) | Independence testing | Not analysed | Not analysed | Not analysed | Not analysed |
| Stubbs et al. (2016) | Random effects model, Meta regression, Subgroup analyses | Depressive symptoms | Participants with more pronounced depressive symptoms were significantly more likely to dropout among those with major depressive disorder | Coefficient [95% CI]; p-value; N - 0.041 [0.081, 0.001]; p=0.04; 29 | Comorbidities, age, sex, taking antidepressants |
| Swift et al. (2017) | Random effects model | Condition treated | Participants with anorexia or bulimia were more likely to premature terminate treatment if assigned to pharmacotherapy compared to psychotherapy | OR [95% CI]; p-value; N - 2.46 [1.00, 6.05]; p<0.05; 2 | None |
|  |  |  | Participants with depression were more likely to premature terminate treatment if assigned to pharmacotherapy compared to psychotherapy | OR [95% CI]; p-value; N - 1.26 [1.00, 1.58]; p<0.05; 44 |  |
|  |  |  | Participants with OCD were less likely to premature terminate treatment if assigned to pharmacotherapy compared to combined therapy | OR [95% CI]; p-value; N - 0.47 [0.24, 0.94]; p<0.05; 3 |  |
|  |  |  | Participants with PTSD were more likely to premature terminate treatment if assigned to pharmacotherapy compared to psychotherapy with a placebo pill | OR [95% CI]; p-value; N - 10.8 [1.26, 92.67]; p<0.05; 1 |  |
| Szymczynska et al. (2017) | Random effects model, Meta regression | None | None | None | Age, sex, duration of illness |
| Tedeschini et al. (2010) | Random effects model, Meta regression | Depression severity | Participants with greater severity of depression had a significantly higher risk of discontinuing antidepressants compared to placebo treatment | p-value - p=0.03 | NA |
| Torous et al. (2020) | Random effects model, Meta regression, Subgroup analyses | None | None | None | Sex, age |
| Vancampfort et al. (2016) | Random effects model, Meta regression, Subgroup analyses | None | None | None | Age, sex, duration of illness, BMI, medication dosage, severity of schizophrenia |
| Vancampfort et al. (2017) | Random effects model, Meta regression | Sex | Studies with a higher % of males had significantly higher dropout rates | Coefficient [95% CI]; p-value - 1.15 [0.06, 1.68]; p=0.048 | Age, duration of illness, employment status, race, smoking status, baseline CD4 count, baseline HIV viral load |
|  |  | BMI | Studies with participants who have higher BMI had significantly higher dropout rates | Coefficient [95% CI]; p-value - 0.15 [0.01, 0.26]; p=0.03 |  |
|  |  | Cardiovascular fitness | Studies with less cardiovascularly fit participants had significantly higher dropout rates | Coefficient [95% CI]; p-value - 0.1 [0.03, 0.16]; p=0.006 |  |
| Vancampfort et al. (2021) | Random effects model, Meta regression, Subgroup analyses | None | None | None | Age, sex, diagnosis |
| Villeneuve et al. (2010) | Random effects model, Meta regression, Subgroup analyses | Age | Older participants were significantly more likely to dropout | Coefficient [95% CI]; p-value - 0.019 [0.001, 0.036]; p=0.032 | Sex, illness severity |
|  |  | Duration of illness | Participants with a longer illness duration were significantly more likely to dropout | Coefficient [95% CI]; p-value - 0.039 [0.020, 0.057]; p=0.00004 |  |
| Wahlbeck et al. (2001) | Two-tailed significance testing, linear ANOVA regression | Not analysed | Not analysed | Not analysed | Not analysed |
| Wasmann et al. (2019) | Random effects model, independence testing, Meta regression | Not analysed | Not analysed | Not analysed | Not analysed |
| Weinmann et al. (2008) | Fixed effects model, Mantel-Haenszel method | Not analysed | Not analysed | Not analysed | Not analysed |
| Windle et al. (2020) | Random effects model, Meta regression, Sensitivity analyses | None | None | None | Age, sex, race, depression or anxiety |
| Zhang et al. (2021) | Random effects model, Meta regression, Sensitivity analyses | Not analysed | Not analysed | Not analysed | Not analysed |
| Zhou et al. (2020) | Random effects model, Meta regression | Not analysed | Not analysed | Not analysed | Not analysed |

## Supplementary Table 7. Additional review characteristics describing the evaluation of trial characteristics.

| **Review** | **Methods** | **Associated trial characteristic** | **Descriptive findings** | **Measures of effect/significance** | **Unassociated trial characteristics** |
| --- | --- | --- | --- | --- | --- |
| Albano et al. (2019) | Random effects model | Treatment arm | Guided self-help and self-help treatment groups had significantly lower end of treatment dropout than comparison treatments | OR [95% CI]; p-value - 0.63 [0.41, 0.95]; p=0.03 | None |
| Aparicio et al. (2016) | Random effects model, Mantel-Haenszel method, Meta regression, ANOVA, Univariate linear regressions | None | None | None | Treatment arm, clinical diagnosis, sample size, treatment duration, current density, number of sessions |
| Arafah et al. (2017) | Random effects model, Univariate and multivariate meta regression | Trial design | Studies using RCT design had significantly lower attrition than non-RCT studies | Coefficient [95% CI]; p-value - -5.6 [-6.7, -4.5]; p<0.0001 | None |
| Bacaltchuk et al. (2001) | Random effects model, Sensitivity analyses | Treatment arm | Active groups had significantly higher dropout due to adverse events than placebo groups | Relative risk [95% CI] - 1.65 [1.05, 2.57] | None |
|  |  | Active drug class | TCA treatment had significantly higher dropout due to any cause than SSRI treatment | Relative risk [95% CI] - 1.93 [1.15, 3.25] |  |
| Benbow et al. (2019) | Random effects model, Mantel-Haenszel method, Meta regression, Subgroup analyses | Between-session intervention | Studies using between-session interventions had significantly lower dropout than those discouraging its use | p-value - 0.03 | Treatment arm, targeted disorder, cognitive-behavioural interventions used in addition to primary treatment, number of sessions |
| Bevens et al. (2022) | Restricted maximum likelihood method, Univariate and multivariate regression | Multimedia elements score | Studies with a greater number of multimedia elements to interventions had significantly lower dropout | Differential attrition [95% CI]; p-value - 1.02 [1.00-1.04); p=0.03 | Publication year, number of sessions, sample size, intervention with placebo pill, deviation bias |
|  |  | Multimedia elements score | Studies with a greater number of multimedia elements to digital interventions had significantly lower dropout | Relative risk [95% CI]; p-value - 1.04 [1.00-1.08]; p=0.03 |  |
|  |  | Overall engagement score | Studies with a higher-scored app intervention for engagement had significantly lower attrition | Relative risk [95% CI]; p-value - 1.01 [1.00-1.03]; p=0.01 |  |
| Albano et al. (2019) | Random effects model | Treatment arm | Guided self-help and self-help treatment groups had significantly lower end of treatment dropout than comparison treatments | OR [95% CI]; p-value - 0.63 [0.41, 0.95]; p=0.03 | None |
| Bighelli et al. (2018) | Random effects model, Frequentist network meta-analysis | Active treatment type | CBT and inactive control had less dropout for any reason than treatment as usual | RR [95% CI]; p-value - RR <1 | Number of sessions, study duration, setting, therapist qualifications, baseline condition severity, open-label, completers-only reporting, risk of bias, risk of allegiance, treatment-resistant focus, treatment arm |
| Bricca et al. (2022) | Mixed effects model, Meta regression | Recruitment method | Studies using indirect recruitment methods had significantly lower retention (higher attrition) | OR [95% CI]; p-value; N - 0.6 [0.97, 0.38]; p=0.04 | Retention strategies, non-smoking related financial incentives, biomarker assessments, primary endpoint, intervention format, treatment frequency, pharmacological support, setting, adjuvant interventions, number of behaviour change techniques (BCTs) used |
|  |  | Follow-up duration | Studies with longer follow-up durations had significantly lower retention (higher attrition) | OR [95% CI]; p-value; N - 0.83 [0.87, 0.79]; p<0.01 |  |
|  |  | Financial incentives for smoking cessation | Studies using financial incentives for smoking cessation had significantly higher retention (lower attrition) in intervention arms compared to comparator arms | OR [95% CI]; p-value; N - 1.35 [1.77, 1.02]; p=0.04 |  |
| Chen et al. (2021) | Frequentist network meta-analysis, Mixed effects model, Generalised linear regression, Subgroup analyses | None | None | None | Treatment arm |
| Cooper et al. (2015) | Random effects model, Mixed effects model, Meta regression, ANOVA-like tests | Treatment arm | Studies using inactive control groups had significantly higher dropout than studies using only active groups | Dropout rate [95% CI]; p-value - 17.8 [14.9, 20.9]; p=0.03 | Population, region, setting, target condition, therapist qualifications, study duration, number of intended sessions, provider experience |
| Cramer et al. (2016) | Random effects model, Subgroup analyses, Chi-square | Intervention elements | Including yoga postures was significantly associated with greater dropout than not including postures | Dropout rate [95% CI]; Chi-sq; p-value - 12.00 [10.53, 13.46]; 8.32; p=0.004 | Study location |
|  |  |  | Including meditation was significantly associated with greater dropout than not including postures | Dropout rate [95% CI]; Chi-sq; p-value - 12.67 [10.75, 14.60]; 3.70; p=0.05 |  |
|  |  | Intervention duration | Longer intervention duration was significantly associated with higher dropout | Dropout rate [95% CI]; Chi-sq; p-value - 15.23 [11.79, 18.68]; 7.21; p=0.03 |  |
|  |  | Treatment arm | Yoga groups had significantly lower dropout compared to exercise control groups | OR [95% CI]; p-value - 0.82 [0.68, 0.98]; p=0.03 |  |
| Bighelli et al. (2018) | Random effects model, Frequentist network meta-analysis | Active treatment type | CBT and inactive control had less dropout for any reason than treatment as usual | RR [95% CI]; p-value - RR <1 | Number of sessions, study duration, setting, therapist qualifications, baseline condition severity, open-label, completers-only reporting, risk of bias, risk of allegiance, treatment-resistant focus, treatment arm |
| Bricca et al. (2022) | Mixed effects model, Meta regression | Recruitment method | Studies using indirect recruitment methods had significantly lower retention (higher attrition) | OR [95% CI]; p-value; N - 0.6 [0.97, 0.38]; p=0.04 | Retention strategies, non-smoking related financial inentives, biomarker assessments, primary endpoint, intervention format, treatment frequency, pharmacological support, setting, adjuvant interventions, number of behaviour change techniques (BCTs) used |
|  |  | Follow-up duration | Studies with longer follow-up durations had significantly lower retention (higher attrition) | OR [95% CI]; p-value; N - 0.83 [0.87, 0.79]; p<0.01 |  |
|  |  | Financial incentives for smoking cessation | Studies using financial incentives for smoking cessation had significantly higher retention (lower attrition) in intervention arms compared to comparator arms | OR [95% CI]; p-value; N - 1.35 [1.77, 1.02]; p=0.04 |  |
| Chen et al. (2021) | Frequentist network meta-analysis, Mixed effects model, Generalised linear regression, Subgroup analyses | None | None | None | Treatment arm |
| Cooper et al. (2015) | Random effects model, Mixed effects model, Meta regression, ANOVA-like tests | Treatment arm | Studies using inactive control groups had significantly higher dropout than studies using only active groups | Dropout rate [95% CI]; p-value - 17.8 [14.9, 20.9]; p=0.03 | Population, region, setting, target condition, therapist qualifications, study duration, number of intended sessions, provider experience |
| Cramer et al. (2016) | Random effects model, Subgroup analyses, Chi-square | Intervention elements | Including yoga postures was significantly associated with greater dropout than not including postures | Dropout rate [95% CI]; Chi-sq; p-value - 12.00 [10.53, 13.46]; 8.32; p=0.004 | Study location |
|  |  | Intervention elements | Including meditation was significantly associated with greater dropout than not including postures | Dropout rate [95% CI]; Chi-sq; p-value - 12.67 [10.75, 14.60]; 3.70; p=0.05 |  |
|  |  | Intervention duration | Longer intervention duration was significantly associated with higher dropout | Dropout rate [95% CI]; Chi-sq; p-value - 15.23 [11.79, 18.68]; 7.21; p=0.03 |  |
|  |  | Treatment arm | Yoga groups had significantly lower dropout compared to exercise control groups | OR [95% CI]; p-value - 0.82 [0.68, 0.98]; p=0.03 |  |
| Crutzen et al. (2015) | Random effects model, Mixed effects model, Univariate and multivariate meta regression | Treatment arm | Intervention groups had significantly higher dropout than control groups | Relative attrition rate [95% CI]; p-value - 1.1 [1.01, 1.2]; p<0.01 | Human contact in delivery, treatment intensity, control type, follow-up intensity, follow-up duration |
| Cuijpers et al. (2009) | Random effects model, Mixed effects model, Meta regression, Subgroup analyses | Treatment arm | Combined treatment groups had significantly lower dropout than pharmacotherapy groups | OR [95% CI]; p-value - 0.65 [0.50, 0.83]; p<0.01 | Population, recruitment method, psychological treatment type, treatment modality, methodology, study design, medication class |
| Cunill et al. (2013) | Random effects model, Meta regression, Fixed effects model, Sensitivity analysis | None | None | None | Treatment duration, dosing regimen, comorbidity as inclusion criterion, lead-in period, recruitment method |
| De Campos Moreira et al. (2017) | Random effects model, Meta regression | Study quality | Lower-quality studies had significantly higher dropout | OR [95% CI]; p-value - 3.3 [1.04, 12.9]; p=0.05 | None |
|  |  | Treatment arm | Vocal training groups had significantly lower dropout | OR [95% CI]; p-value - 0.14 [0.07, 0.27]; p=0.04 |  |
| DeCrescenzo et al. (2018) | Random effects model, Network meta-analysis, Subgroup network meta-analysis | Treatment arm | Groups receiving combined contingency management and community reinforcement, community reinforcement alone, non-contingent rewards, contingency management along and CBT had significantly less dropout than treatment as usual groups | OR range [95% CI]; p-value - 1.41 [1.10, 1.82] - 3.63 [2.01, 6.55]; p<0.001 | Treatment intensity, publication year, sample size |
|  |  |  | Combined contingency management and community reinforcement groups had significantly less dropout than CBT, contingency management alone, contingency management and CBT, community reinforcement and non-contingent rewards, meditation-based therapy, non-contingent rewards alone, supportive-expressive psychodynamic therapy, 12-step programme along and 12-step programme combined with non-contingent rewards | OR range [95% CI]; p-value - 2.06 [1.04, 4.08] - 4.61 [1.92, 11.06]; p<0.001 |  |
| Dixon et al. (2020) | Random effects model, Borenstein et al. method, Mixed effects model, Subgroup analyses | Therapist attending consultant team meetings | Trials reporting therapist attendance at consultation meetings had significantly higher dropout than those that did not report attendance | Dropout rate [95% CI]; p-value - 33.1 [27.5, 39.2]; p=0.012 | Targeted disorder, dropout definition, delivery format, therapist experience, therapist adherence, treatment arm |
|  |  | Availability of telephone crisis coaching | Trials reporting between-session telephone crisis coaching had significantly lower dropout than those that did not report this | Dropout rate [95% CI]; p-value - 35.6 [30.1, 41.6]; p<0.001 |  |
| Doyle et al. (2021) | Random effects model, Frequentist network meta-analysis, Subgroup analyses | None | None | None | Treatment arm |
| Dudas et al. (2018) | Fixed effects, Mantel-Haenszel method | Treatment arm | Antidepressant groups had significantly lower dropout than placebo groups over 6-13wks | OR [95% CI]; p-value - 1.39 [0.69, 2.80]; p=0.02 | None |
| Edwards-Stewart et al. (2021) | Random effects model, Unstructured correlation matrix, Subset analysis | Treatment type | Trauma-focused treatment had a higher risk of dropout than non-TFT treatments | RR [95% CI]; N - 1.6 [1.29, 1.99]; I-sq=0%;12 | Population, country or study, total sample size, active treatment frequency, session frequency, use of telehealth, use of incentives, originator of active treatment being part of study team |
|  |  |  | Trauma-focused treatment had a higher risk of dropout than waitlist treatments | RR [95% CI]; N - 1.68 [1.11, 3.76]; I-sq=26.6%; 8 |  |
| Elsner et al. (2020) | Random effects model, Mantel-Haenszel method | None | None | None | Treatment arm |
| Fabricatore et al. (2009) | Mixed effects model | Treatment type | Placebo groups had significantly higher total attrition compared to active groups | F-stat; p-value - 12.02; p<0.0001 | Number of visits, number of lifestyle visits, study location |
|  |  | Lead-in period | Groups with a lead-in period had significantly lower total attrition | F-stat; p-value - 7.76; p<0.01 |  |
|  |  | Comorbidity as inclusion criterion | Studies that selected participants based on the presence of a weight-based comorbidity had significantly lower non-AE-related attrition | Mean non-AE-related attrition (SD); p-value - 29.9 (3.0); p=0.05 |  |
| Frampton et al. (2003) | Peto odds ratio | Treatment arm | Active groups had significantly higher dropout than placebo groups | OR [95% CI]; p-value - 1.43 [1.04, 1.90]; p=0.03 | None |
| Furukawa et al. (2020) | Random effects model | None | None | None | Treatment delivery format |
| Gagliardi et al. (2019) | Random effects model, Subgroup analyses, Sensitivity analyses | None | None | None | Treatment arm |
| Gehling et al. (2011) | Random effects model, Fixed effects model | Treatment arm | Active groups had significantly higher total dropout than placebo groups | OR [95% CI]; p-value - 1.315 [1.197, 1.445]; p<0.0001 | Methodological quality |
|  |  | Trial duration | Shorter trials had higher dropout than longer trials | OR [95% CI] - 3.1 [2.203, 4.478] |  |
| Goldberg et al. (2021) | Random effects model, Meta regression | Condition type, Sample size | Participants with active conditions were significantly more likely to dropout than participants with passive conditions as study sample size increased | Log OR [95% CI]; p-value - 0.0022 [0.0005-0.0039]; p=0.01 | None |
| Harris et al. (2021) | Random effects model, Meta regression, Subgroup analyses | None | None | None | Intervention components, intervention type, session frequency, session intensity, session volume, delivery format, setting, intervention duration, supervision used, intervention tailoring, intervention adherence |
| Heneghan et al. (2007) | DerSimonian-Laird random effects model, Meta regression | Treatment arm | Self-monitoring oral anticoagulation groups had significantly higher attrition than control groups | Relative attrition; Chi-square; p-value - 6.05; 12091; p<0.001 | None |
| Heo et al. (2009) | Mantel-Haenszel method, Univariate and multivariate logistic regression, Forward stepwise correlation analysis | Sample size | Studies with larger sample size had a significantly lower attrition rate | OR [95% CI]; p-value - 0.96 [0.93, 0.98]; p<0.0001 | None |
|  |  | Treatment arm | Placebo groups had significantly lower attrition than active groups | OR [95% CI]; p-value - 0.70 [0.60, 0.82]; p<0.0001 |  |
|  |  | Number of arms | Studies with more arms had significantly higher attrition | OR [95% CI]; p-value - 1.21 [1.09, 1.35]; p=0.0006 |  |
|  |  | Treatment allocation | Studies with unbalanced treatment allocation had significantly higher attrition rates than studies with balanced treatment allocation | OR [95% CI]; p-value - 1.97 [1.68, 2.31]; p<0.0001 |  |
|  |  | Duration | Longer studies had a significantly higher attrition rate than shorter studies | OR [95% CI]; p-value - 1.76 [1.44, 2.14]; p<0.0001 |  |
|  |  | Cohort type | Studies of mixed settings had significantly higher attrition rates than studies of inpatients | OR [95% CI]; p-value - 1.35 [1.12, 1.64]; p=0.002 |  |
|  |  | Study type | Placebo-controlled trials had significantly lower attrition rates than active comparator trials with no placebo arm | OR [95% CI]; p-value - 0.90 [0.82, 1.00]; p=0.042 |  |
|  |  | Location | US-based studies had significantly higher attrition rates than studies conducted in other countries | OR [95% CI]; p-value - 1.23 [1.12, 1.36]; p<0.0001 |  |
|  |  | Number of centres | Single centre studies had significantly higher attrition rates than multi-centre studies | OR [95% CI]; p-value - 1.17 [1.04, 1.32]; p=0.008 |  |
| Hernandez-Rodriguez et al. (2022) | Random effects model, Meta regression, Sensitivity analyses, Subgroup analyses | None | None | None | Treatment arm, intervention duration, sample size |
| Hornyak et al. (2014) | Random effects model, Mantel-Haenszel | Treatment arm | Ropinirole groups had significantly higher dropout due to adverse events than placebo groups | OR [95% CI]; p-value - 1.76 [1.31, 2.38] | Active control type, active treatment type |
| Hrobjartsson et al. (2014) | Random effects model | Blinded groups | The risk of dropout in nonblind control groups was 79% higher than blinded control groups | Average risk ratio (range) - 1.79 (1.18 - 2.70) | None |
| Huang et al. (2014) | Random effects model, Fixed effects model, Sensitivity analyses | None | None | None | Active treatment type |
| Ibrahim et al. (2016) | Direct comparison, Chi-square, Fisher's exact test | Treatment arm | Placebo groups had significantly higher dropout than active groups | Mean relative dropout rate [95% CI] - 1.61 [1.29, 2.02] | None |
| Iliakis et al. (2021) | Random effects model, Metaregression | None | None | None | Treatment arm, treatment duration, treatment intensity, setting, treatment orientation, trial randomisation, use of phone coaching, presence of consultation team, comorbidity targeted in addition to primary condition (BPD) |
| Imel et al. (2013) | Random effects model, Meta regression, Sensitivity analyses | Treatment modality | Studies using group treatment modality had significantly higher dropout | Coefficient [95% CI]; p-value - 0.12 [0.03, 0.21]; p=0.009 | None |
|  |  | Number of sessions | Studies with a greater number of sessions had significantly higher dropout than studies with less sessions | Coefficient [95% CI]; p-value - 0.01 [0.00, 0.015] |  |
|  |  | Study size | Smaller studies had significantly higher dropout than larger studies | Rank-correlation test - 0.28; p=0.003 |  |
|  |  | Treatment arm | Trauma-focused treatment had significantly higher dropout than PCT | OR [95% CI]; p-value - 2.02; p=0.0009 |  |
| Jabardo-Camprubi et al. (2020) | Random effects model, Subgroup analyses | Treatment duration | High intensity exercise dropout ratio was significantly associated with protocol duration used | OR [95% CI]; p-value - 1.81 [1.13, 2.9]; p=0.01 | Treatment protocols |
| Karyotaki et al. (2015) | Bivariate and multivariate Poisson regression, Sensitivity analyses | None | None | None | Intervention type, number of modules |
| Kato et al. (2021) | Random effects model, Subgroup analyses, Metaregression | Active treatment type | Placebo groups had signifiantly higher dropout than active antidepressant groups | R-squared; p-value - 29.7; p=0.02 | Study duration, treatment discontinuation method, dosing schedule, run-in treatment duration, maintenance treatment duration, location, study year, targeted condition |
| Kline et al. (2021) | Random effects model | Not analysed | Not analysed | Not analysed | Not analysed |
| Koog et al. (2013) | Random effects model, Meta regression | Treatment delivery route | Trials using oral or topical delivery routes had significantly higher dropout than trials using percutaneous and surgical routes | p-value - p<0.001 | Treatment arm |
|  |  | Trial duration | Longer trials had significantly lower dropout due to ineffectiveness | Proportion [95% CI]; p-value - -0 [-0, -0]; p<0.001 |  |
|  |  | Trial design | Flare design trials had significantly higher dropout due to ineffectiveness | Proportion [95% CI]; p-value - 6 [2, 11]; p<0.001 |  |
|  |  | Medication restrictions | Prohibition of usual analgesics was associated with significantly higher dropout due to ineffectiveness | Proportion [95% CI]; p-value - 2 [0, 4]; p<0.001 |  |
|  |  | Prohibited escape medication | Allowing the use of escape medications was significantly associated with higher dropout | Proportion [95% CI]; p-value - -2 [-6, -0]; p=0.005 |  |
| Kredo et al. (2013) | Random effects model, Subgroup analyses | None | None | None | Model of care |
| Lam et al. (2022) | Random effects model, Peto method, Mantel-Haenszel method, Sensitivity analyses, Subgroup analyses, Meta regression | Having trained facilitators | Having trained facilitators for control conditions was significantly associated with lower differential attrition | Coefficient; p-value - -0.34; p=0.026 | Non-clinical population, number of sessions, active treatment type, control treatment type, treatment format, treatment adaptation, mindfulness-based relapse prevention programmes, publication year, country |
|  |  | Recruitment setting | Recruitment in a clinical setting was associated with higher overall attrition | Coefficient; p-value - 0.07; p=0.022 |  |
|  |  | Use of mindfulness-based relapse prevention programmes | Use of MBRP was associated with higher overall attrition for control participants | Coefficient; p-value - 0.13; p=0.019 |  |
| Leucht et al. (2017) | Random effects model, Bayesian hierarchical framework | Treatment arm | Placebo groups were significantly more likely to prematurely discontinue treatment for any reason than active groups | Risk ratio [95% CI]; N - 1.25 [1.20, 1.31]; 105 | None |
|  |  |  | Placebo groups were significantly more likely to prematurely discontinue treatment due to inefficacy than active groups | Risk ratio [95% CI]; N - 2.09 [1.90, 2.32]; 94 |  |
| Levinson et al. (2022) | Random effects model, Univariate and multivariate regression, Sensitivity analysis | Treatment frequency | More frequent sessions were associated with less dropout | Univariate OR [95% CI]; p-value; N - 0.52 [0.30, 0.89]; p=0.018; 35 | Publication year, number of sessions, sample size, intervention with placebo pill, deviation bias |
|  |  | Treatment frequency |  | Multivariate OR [95% CI]; p-value; N - 0.40 [0.20, 0.78]; p=0.008; 35 |  |
|  |  | Location | US-based studies had significantly higher dropout rates | OR [95% CI]; p-value; N - 2.09 [1.08, 4.04]; p<0.05; 35 |  |
| Lewis et al. (2020) | Random effects model, Meta regression | Trauma focused therapy | Therapies with a trauma focus were significantly associated with higher dropout compared to therapies without a trauma focus | Coefficient [95% CI]; p-value - 0.069 [0.011, 0.127]; p=0.021 | Delivery format, recruitment format |
| Li et al. (2020) | Random effects model, Univariate and multivariate regression, Subgroup analyses | Publication year | Older studies were significantly associated with higher dropout | Coefficient [95% CI]; p-value - -0.11 [-0.16, -0.06); p<0.001 | Population, sample size, number of active arms, response rate, number of visits |
|  |  | Placebo lead-in period | Studies using a placebo lead-in had significantly higher dropout | Coefficient [95% CI]; p-value - 0.18 [0.06, 0.29]; p<0.01 |  |
|  |  | Study location | Studies conducted in one country had significantly higher dropout | Coefficient [95% CI]; p-value - 0.19 [0.08, 0.3]; p<0.001 |  |
|  |  | Trial duration | Studies of longer durations had significantly higher dropout | Coefficient [95% CI]; p-value - 0.07 [0.01, 0.12]; p<0.05 |  |
|  |  | Number of sites | Studies with less sites had significantly higher dropout | Coefficient [95% CI]; p-value - -0.09 [-0.15, -0.04]; p<0.001 |  |
| Linardon et al. (2018) | Mixed effects model, Meta regression, Subgroup analyses | Treatment modality | Studies using e-CBT had significantly higher dropout than other CBT modalities | Proportion [95% CI]; p-value - 0.33 [0.25, 0.34]; p=0.010 | Study quality, dropout definition, quality assessment |
|  |  | Treatment protocol | Studies using transdiagnostic CBT-E had significantly lower dropout than studies using other CBT protocols | Proportion [95% CI]; p-value - 0.18 [0.13, 0.15]; p=0.042 |  |
|  |  | Number of sessions | A greater number of sessions was significantly associated with lower dropout | Coefficient (SE); p-value - -0.03 (0.01); p=0.032 |  |
| Linardon et al. (2019) | Random effects model, Borenstein et al. method, Mixed effects model, Meta regression, Subgroup analyses | Dropout definition | Studies defining dropout as failure to complete the full course of therapy had significantly higher dropout than studies defining dropout as failure to complete at least 50% of sessions or not specifying a definition | Dropout rate [95% CI]; p-value - 15.1 [10.5, 21.2]; p=0.003 | Treatment format, session duration, exclusion based on comorbidities |
|  |  | Treatment arm | IPT groups had significantly lower dropout compared to other psychotherapy treatments | Dropout rate [95% CI]; p-value - 1.56 [1.25, 1.96]; p<0.001 |  |
| Linardon et al. (2020) | Random effects model, Mixed effects model, Subgroup analyses | Treatment arm | Smartphone intervention groups had significantly higher dropouts than inactive control groups | OR [95% CI]; p-value - 1.87 [1.45, 2.41]; I-sq = 55%; p<0.001 | Population, interventions with mindfulness elements |
|  |  | Intervention format | Trials using acceptance-based interventions had significantly lower attrition than those that did not use them | Attrition rate [95% CI]; p-value - 15.6 [10.3, 22.9]; p=0.024 |  |
|  |  | Financial incentives | Trials offering compensation for post-test questionnaire completion had significantly lower attrition than those that did not offer compensation | Attrition rate [95% CI]; p-value - 17.7 [12.4, 24.7]; p=0.027 |  |
|  |  | Reminders for engagement | Trials giving reminders for app engagement had significantly lower attrition than those that did not give reminders | Attrition rate [95% CI]; p-value - 20.0 [15.8, 25.0]; p=0.015 |  |
|  |  | Enrolment method | Trials using online enrolment had significantly higher attrition than those using telephone or in-person interviews prior to enrolment | Attrition rate [95% CI]; p-value - 43.4 [35.3, 51.9]; p<0.001 |  |
| Makatsori et al. (2014) | Random effects model, Subgroup analyses | None | None | None | Study duration, treatment formulation, treatment schedule, number of centres, number of sensitisations, country of study |
| Martin et al. (2006) | Random effects model, Fixed effects model, Sensitivity analyses | Drug class | Typical antipsychotics on a flexible dose regimen were associated with a significantly lower risk of all-cause discontinuation than atypical antipsychotics on a flexible dose regimen | (Short-term) Risk ratio [95% CI]; p-value - 0.7 [0.64, 0.76]; p<0.00001 | None |
|  |  |  | Atypical antipsychotics had a significantly lower risk of all-cause discontinuation and discontinuation due to adverse events both in the short and long term than typical active comparators on flexible dosing regimens | (Long-term) Risk ratio [95% CI]; p-value - 0.72 [0.65, 0.80]; p<0.00001 |  |
|  |  |  | Atypical antipsychotics had a significantly lower risk of all-cause discontinuation in the long term than typical active comparators on flexible dosing regimens | (Short-term AE) Risk ratio [95% CI]; p-value - 0.54 [0.41â€“0.72]; p<0.0001 |  |
|  |  |  | Atypical antipsychotics had a significantly lower risk of discontinuation due to adverse events than typical active comparators on flexible dosing regimens | (Long-term AE) Risk ratio [95% CI]; p-value - 0.73 [0.56, 0.95]; p=0.02 |  |
| Matsusaki et al. (2019) | Random effects model, Univariate and multivariate meta regression | Publication year | Recent publications had significantly lower dropout than older publications | Coefficient (SE); p-value - -0.012 (0.004); p=0.002 | Placebo lead-in period, number of arms, placebo randomisation rate, treatment regimen, active comparator class, setting, number of countries, number of randomised subjects per site |
|  |  | Study duration | Longer studies had significantly higher dropout | Coefficient (SE); p-value - 0.214 (0.056); p=0.000 |  |
|  |  | Number of sites | Studies with more sites had significantly higher dropout | Coefficient (SE); p-value - 0.001 (0.000); p=0.007 |  |
| McVay et al. (2023) | Random effects model, Meta regression | Run-in period | The presence of a behavioural run-in period was associated with a trend towards higher 12-month retention (lower 12-month attrition) | Estimate [95% CI]; p-value - 4.6 [-0.1, 9.4]; p=0.057 | None |
| Minozzi et al. (2020) | Random effects model | None | None | None | Treatment arm |
| Mitsikostas et al. (2012) | Random effects model, Mixed effects model, Meta regression | Treatment arm | Placebo arm efficacy was significantly positively correlated with dropouts in placebo arms | Pearson's r; p-value - 0.0596; p<0.001 | Study year, publication year, methodological quality |
|  |  | AE frequency | Frequency of dropouts due to AE was significantly positively correlated with frequency of AEs | Pearson's r; p-value - 0.915; p<0.0001 |  |
|  |  | Use of nocebo | RCTs for fibromyalgia had significantly higher nocebo dropouts than RCTs for other conditions | Dropout rate [95% CI] - 9.5 [8.3, 10.9] |  |
|  |  | Number of participants | Studies with more participants had significantly higher dropout | p-value - p=0.0251 |  |
|  |  | Number of placebo participants | Studies with more placebo participants had significantly higher dropouts due to AE in placebo arms | p-value - p=0.0009 |  |
| Miyasaka et al. (2006) | Fixed effects model | None | None | None | Drug class |
| Ong et al. (2016) | Random effects model, Meta regression, ANOVA | None | None | None | Therapist qualifications, number of sessions |
| Ong et al. (2018) | Random effects model, Meta regression | Therapist qualifications | Study treatment being delivered by a master’s level clinician was associated with significantly higher dropout than treatment delivered by a psychologist | Weighted dropout rate [95% CI]; p-value - 29.9 [17.6, 43.8]; p=0.019 | Targeted condition, treatment arm, treatment format, number of sessions, session frequency, methodological quality |
| Palmowski et al. (2020) | Random effects model, Mixed effects model, Meta regression, Sensitivity analyses | Study duration | Studies of longer duration had significantly lower retention (higher attrition) | Coefficient [95% CI]; p-value - -0.005 [-0.005, -0.002]; p<0.001 | Recruitment age limit, region, study size |
|  |  | Intervention arm | Surgical intervention groups had significantly higher retention (lower attrition) than psychological intervention groups | p-value - p<0.001 |  |
| Pampallona et al. (2004) | Random effects model, Sensitivity analysis, Metaregression | Study duration | Longer studies were associated with significantly lower dropout than shorter studies | OR [95% CI]; p-value (metaregression) - 0.59 [0.39, 0.88]; p=0.02 | Treatment arm, study quality |
| Papadopoulos et al. (2010) | Random effects model, Meta regression | Publication year | Recent trials had significantly higher nocebo withdrawals | Coefficient; p-value - 0.0036; p=0.0348 | Treatment arm, population, administration route, treatment duration |
|  |  | Trial phase | Phase II trials had significantly lower dropout due to drug-related adverse reactions than phase III trials | Coefficient; p-value - -0.1031; p<0.0001 |  |
|  |  | Treatment frequency | Studies using greater frequencies of treatment had significantly higher dropout due to drug-related adverse reactions | Coefficient; p-value - 0.0633; p=0.0082 |  |
| Pozza et al. (2017) | Random effects model | None | None | None | Treatment arm |
| Rabinowitz et al. (2009) | Mixed effects model, Meta regression, Subgroup analyses | Active drug class | 1st gen antipsychotic groups had significantly higher dropout rate than 2nd gen antipsychotic groups | OR [95% CI]; p-value - 1.49 [1.31, 1.66]; p=0 | None |
|  |  | Trial duration | Longer studies had a significantly lower attrition rate in placebo groups than shorter studies | Estimate (SE); p-value - 0.94 (0.33); p=0.004 |  |
|  |  | Treatment modality | Fixed dosing of 2nd gen antipsychotics was significantly associated with higher dropout than flexible dosing | OR [95% CI]; p-value - 8.23 [3.39, 2.43]; p=0.01 |  |
| Reas et al. (2008) | Fixed effects model, Random effects model | None | None | None | Treatment arm |
| Rehman et al. (2021) | Random effects model | None | None | None | Treatment arm |
| Rutherford et al. (2013) | Mixed effects model, Logistic regression | Study type | Comparator studies had significantly lower dropout than placebo-controlled studies | Coefficient (SE); p-value - -0.28 (0.11); p<0.05 | Treatment arm |
|  |  | Treatment duration | Studies with longer treatment duration had significantly lower dropout than studies with shorter treatment durations | Coefficient (SE); p-value - -1.11 (0.33); p<0.05 |  |
|  |  | Visit frequency | Studies with more visits had significantly higher dropout than studies with less visits | Coefficient (SE); p-value - 2.77 [1.66, 4.63]; p<0.001 |  |
| Schalkwijk et al. (2014) | Random effects model, Linear regression, Meta regression | Reporting year | Older RCT reports for antidepressants had significantly higher overall dropout than more recent RCT reports | Coefficient [95% CI]; p-value - 1.23 [-1.56, -0.897]; p<0.0001 | None |
|  |  | Treatment arm | Antidepressant dropouts due to inefficacy were positively and significantly correlated to placebo dropouts | Coefficient [95% CI]; p-value - 1.03 [0.81, -1.26]; p<0.0001 |  |
| Shah et al. (2020) | Random effects model, Mantel-Haenszel method, Two-tailed test, Sensitivty analysis | None | None | None | Treatment arm |
| Somerson et al. (2016) | Univariate comparisons, Independence testing, Correlation testing, Linear regression | Trial type | Trials of shoulder and elbow surgery reported significantly higher loss to follow-up than all other trial types | p-value - p=0.04 | Impact factor, sample size, orthopaedic speciality |
|  |  | Follow-up strategy | Trials using remote follow-up strategies reported significantly higher reported loss to follow-up than trials using an in-office follow-up strategy | p-value - p=0.01 |  |
|  |  | Funding source | Trials with foundation or institute funding reported significantly higher loss to follow-up than trials funded by government, industry or unreported sources | p-value - p<0.01 |  |
|  |  | Location | Trials conducted in the US had significantly higher loss to follow-up than trials conducted in non-US countries | p-value - p=0.05 |  |
|  |  | Follow-up duration | Studies with a minimum follow-up length of three years had significantly higher loss to follow-up than studies with shorter minimum follow-up time | p-value - p=0.01 |  |
| Song et al. (2021) | Quasi-poisson regression | Study design | Efficacy studies had significantly higher retention (lower attrition) than non-efficacy studies | Coefficient (SE); p-value - 0.46 (0.22); p<0.05 | Number of arms, enrolment setting, enrolment method |
|  |  | Delivery format | Studies using material provisions (i.e. booklets) instead of in-person delivery had significantly higher retention (lower attrition) | Coefficient (SE); p-value - 0.58 (0.24); p<0.05 |  |
|  |  | Delivery format | Studies using more than 2 delivery formats had significantly higher retention (lower attrition) than studies using in-person delivery | Coefficient (SE); p-value - 0.74 (0.21); p<0.01 |  |
|  |  | Follow-up duration | Studies with a follow-up duration >=6m had significantly lower retention (higher attrition) than studies with short follow-up duration | Coefficient (SE); p-value - -0.52 (.12); p<0.001 |  |
|  |  | Intervention duration | Studies with intervention durations >=16wks had significantly higher retention (lower attrition) thant studies with shorter intervention durations | Coefficient (SE); p-value - 0.3 (0.1); p=<0.01 |  |
| Stahl et al. (1993) | Independence testing | Trial design | Overall dropout rates were significantly higher in parallel trials than in crossover trials | Dropout rate, p-value - 25% vs 9%; p<0.001 | Treatment arm |
|  |  | Trial design | Dropouts due to adverse events and perceived lack of effect were significantly more common in parallel trials than in crossover trials | Dropout rate, p-value - 5% vs 3%; p=0.004 |  |
|  |  | Drug-treatment group | Dropouts due to adverse events were significantly more common in drug-treatment groups compared to placebo | Dropout rate, p-value - 7% vs 2%; p<0.001 |  |
|  |  | Placebo group | Dropouts due to perceived lack of effect were significantly more common in placebo groups | Dropout rate, p-value - 6% vs 2%; p<0.001 |  |
| Stubbs et al. (2016) | Random effects model, Meta regression, Subgroup analyses | Setting | Studies of inpatient settings had significantly lower dropout than studies of outpatient settings | Coefficient [95% CI]; p-value - -1.407 [-2.472, -0.342]; p=0.009 | Study quality, publication type, group exercise, supervision |
|  |  | Intervention type | Strength training interventions had significantly lower dropout than aerobic or mixed exercise interventions | Coefficient [95% CI]; p-value - 1.78 [0.4, 3.15]; p=0.01 |  |
|  |  | Supervisor qualifications | Studies where physiotherapists delivered interventions had significantly lower dropout | Coefficient [95% CI]; p-value - -12.03 [-2.097, -0.309]; p=0.008 |  |
|  |  | Treatment arm | Exercise groups involving participants with depression had significantly less dropout than control groups | OR [95% CI]; p-value; N - 0.64 [0.43, 0.95]; p=0.02; 29 |  |
| Swift et al. (2017) | Random effects model | Treatment arm | Pharmacotherapy groups were significantly more likely to prematurely terminate than psychotherapy groups in comparator trials | OR [95% CI]; p-value; N - 1.2 [1.03, 1.41]; p<0.05; 85 | None |
| Szymczynska et al. (2017) | Random effects model, Meta regression | Number of sessions | Studies with a greater number of sessions in experimental interventions had significantly higher dropout | Coefficient [95% CI]; p-value - 0.97 [0.28, 1.67]; p=0.011 | Study location, setting, control intervention used, duration, number of evaluations, study quality, number of follow-up assessments |
| Tedeschini et al. (2010) | Random effects model, Meta regression | Treatment arm | Antidepressant groups had significantly lower dropout than placebo groups | Risk ratio [95% CI]; p-value - 0.95 [0.91, 0.99]; p=0.017 | Trial duration, dosing schedule, probability of receiving placebo |
|  |  | Publication year | Older publications had significantly higher dropout in antidepressant groups than placebo groups | p-value - p=0.04 |  |
| Torous et al. (2020) | Random effects model, Meta regression, Subgroup analyses  analyses | Intervention elements | Interventions with integrated mood monitoring on apps had significantly lower dropout than those that did not incorporate this element | Dropout rate [95% CI]; p-value - 18.42 [10.92, 29.38]; p=0.037 | Treatment arm, population, eligibility criteria, financial incentives, intervention duration |
|  |  | Intervention elements | Interventions with human feedback for users had significantly lower dropout than those that did not incorporate this element | Dropout rate [95% CI]; p-value - 11.74 [23.53, 46.22]; p=0.003 |  |
|  |  | Sample size | Studies with larger sample sizes had significantly higher dropout | Coefficient (SE); p-value - 0.00826 (0.00415); p=0.046 |  |
| Vancampfort et al. (2016) | Random effects model, Meta regression, Subgroup analyses | Treatment arm | Physical activity groups had significantly higher dropout than control groups | OR [95% CI]; p-value - 2.15 [1.29, 3.58]; p=0.003 | Type of physical activity intervention |
|  |  | Setting | Outpatient studies had significantly higher dropout than inpatient studies | Dropout rate [95% CI]; p-value - 24.6 [16.3, 25.3]; p=0.023 |  |
|  |  | Intervention intensity | Physical interventions of higher intensity had significantly lower dropout than low-moderate intensity interventions | Coefficient [95% CI]; p-value - -1.263 [-1.57, -0.95]; p<0.001 |  |
|  |  | Motivational elements of intervention | Interventions with motivational components had significantly lower dropout than interventions without them | Dropout rate [95% CI]; p-value - 13.4 [5.6, 28.7]; p=0.002 |  |
|  |  | Supervision format | Studies with supervised interventions had significantly lower dropout than studies using unsupervised interventions | Coefficient [95% CI]; p-value - -0.64 [-1.29, 0.01]; p=0.05 |  |
|  |  | Supervisor qualification | Studies with adequately qualified supervisors had significantly lower dropout than studies with lower-level qualified supervisors | Coefficient [95% CI]; p-value - -1.06 [-1.77, -0.35]; p=0.003 |  |
| Vancampfort et al. (2017) | Random effects model, Meta regression | Treatment type | Resistance training groups had significantly lower dropout than aerobic training groups | p-value - 0.003 | Setting, motivational elements, treatment modality, intervention frequency, intervention intensity |
|  |  | Supervision format | Studies using supervised interventions had significantly lower dropout than studies using unsupervised interventions | p-value - <0.001 |  |
|  |  | Clinician qualifications | Studies with adequately qualified clinicians had significantly lower dropout than studies with lower-level qualified professionals | p-value - <0.001 |  |
| Vancampfort et al. (2021) | Random effects model, Meta regression, Subgroup analyses | Setting | Studies in outpatient settings had significantly higher dropout than inpatient settings | Proportion [95% CI]; p-value - 17.9 [11.8, 26.5]; p<0.001 | Treatment arm, intervention type, intervention intensity |
|  |  | Intervention frequency | Greater frequency of exercise interventions was significantly associated with higher dropout | p-value - p<0.001 |  |
|  |  | Intervention duration | Longer duration of exercise interventions was significantly associated with higher dropout | Proportion [95% CI]; p-value - p<0.001 |  |
|  |  | Supervision format | Studies with unsupervised interventions had significantly higher dropout | Proportion [95% CI]; p-value - 18.2 [9.6, 31.9]; p<0.001 |  |
|  |  | Qualifications of supervisor | Supervision done by healthcare professionals with experience in exercise prescription was significantly associated with lower dropout | p-value - p<0.001 |  |
|  |  | Motivational strategies | Studies using controlled motivational strategies had significantly higher dropout | p-value - p<0.001 | Treatment arm, intervention type, intervention intensity |
| Villeneuve et al. (2010) | Random effects model, Meta regression, Subgroup analyses | Study quality | Studies in high-impact journals had significantly lower dropout than studies in lower-impact journals | Coefficient [95% CI]; p-value –  -0.033 [-0.060, 0.004]; p=0.024 | Treatment modality |
|  |  | Treatment duration | Studies with longer treatment duration had significantly higher dropout than studies with shorter treatment duration | Coefficient [95% CI]; p-value - 0.003 [0.0001, 0.004; p=0.035 |  |
| Wahlbeck et al. (2001) | Two-tailed significance testing, linear ANOVA regression | Publication year | Older publications had significantly lower dropout than newer publications | F-statistic; p-value - 97.2; p<0.0001 | None |
|  |  | Active drug class | Conventional antipsychotic treatment groups had significantly higher dropout than atypical antipsychotic treatment groups | F-statistic; p-value - 8.0; p=0.0004 |  |
|  |  | Trial duration | Longer studies had significantly higher dropout than shorter studies | F-statistic; p-value - 5.0; p=0.0260 |  |
| Wasmann et al. (2019) | Random effects model, independence testing, Meta regression | Randomised patient preference treatment arm | Randomised cohorts had significantly higher loss to follow-up than preference cohorts | Risk ratio [95% CI]; p-value - 1.30 [1.0, 1.6]; p=0.03 | None |
|  |  | Use of crossover | Randomised crossover cohorts had significantly higher loss to follow-up than preference crossover cohorts | Risk ratio [95% CI]; p-value - 2.60 [1.07, 3.90]; p<0.001 |  |
| Weinmann et al. (2008) | Fixed effects model, Mantel-Haenszel method | Drug class | Venlafaxine treatment was significantly associated with higher dropout due to adverse events than SSRIs | Risk ratio; p-value - 1.38 [1.08, 1.77] | None |
| Windle et al. (2020) | Random effects model, Meta regression, Sensitivity analyses | Preferred accommodation | Studies providing participants with their preferred accommodation had significantly lower dropout | Pooled risk ratio [95% CI]; p-value - 0.62 [0.48, 0.8]; p=<0.001 | None |
|  |  | Preferred treatment | Studies providing participants with their preferred treatment had significantly lower dropout | Pooled risk ratio [95% CI]; p-value - 0.48 0.15, 0.82]; p=0.01 |  |
| Zhang et al. (2021) | Random effects model, Meta regression, Sensitivity analyses | None | None | None | Treatment arm |
| Zhou et al. (2020) | Random effects model, Meta regression | None | None | None | Treatment arm, publication year, treatment dose, study quality |

## Supplementary Table 8. Summary of conditions studied by included reviews. Total number of reviews describes the number of reviews among included reviews that studied conditions described by a condition group (N/Included reviews (%)). Number of reviews describes the number of reviews that studied a condition within a condition group (N/Total reviews in a condition group (%)).

| **Condition group** | **Total number of reviews** | **Condition** | **Number of reviews** |
| --- | --- | --- | --- |
| Addiction | 4/88 (4.55) | Smoking cessation | 2/4 (50) |
|  |  | Addiction | 2/4 (50) |
| Allergy | 1/88 (1.14) | Allergy | 1/1 (100) |
| Cancer | 2/88 (2.27) | Cancer | 2/2 (100) |
| Cardiometabolic conditions | 7/88 (7.95) | Obesity | 3/7 (42.86) |
|  |  | Type 2 diabetes | 2/7 (28.57) |
|  |  | Stroke | 1/7 (14.29) |
|  |  | Coronary artery disease | 1/7 (14.29) |
| Chronic conditions | 3/88 (3.41) | Multimorbidity | 1 (33.33) |
|  |  | Chronic pain | 1 (33.33) |
|  |  | Fibromyalgia | 1 (33.33) |
| Ears, nose and throat conditions | 2/88 (2.27) | Vocal rehabilitation | 1/2 (50) |
|  |  | Tinnitus | 1/2 (50) |
| Infectious diseases | 3/88 (3.41) | HIV | 2/3 (66.67) |
|  |  | Herpes zoster | 1/3 (33.33) |
| Mixed conditions | 5/88 (5.68) | Mixed conditions | 5/5 (100) |
| Musculoskeletal conditions | 5/88 (5.68) | Rheumatoid arthritis | 2/5 (40) |
|  |  | Knee osteoarthritis | 1/5 (20) |
|  |  | Osteoarthritis | 1/5 (20) |
|  |  | Orthopaedic surgery | 1/5 (20) |
| Neurological conditions | 6/88 (6.82) | Multiple sclerosis | 3/6 (50) |
|  |  | Dementia | 2/6 (33.33) |
|  |  | Restless leg syndrome | 1/6 (16.67) |
| Psychological conditions | 50/88 (56.82) | Depression | 15/50 (30) |
|  |  | Schizophrenia | 9/50 (18) |
|  |  | Mixed psychological disorders | 8/50 (16) |
|  |  | PTSD | 6/50 (12) |
|  |  | Eating disorders | 4/50 (8) |
|  |  | Anxiety | 3/50 (6) |
|  |  | OCD | 2/50 (4) |
|  |  | Acute mania | 1/50 (2) |
|  |  | Personality disorders | 1/50 (2) |
|  |  | ADHD | 1/50 (2) |

## Supplementary Table 9. Summary of quality appraisal using R-AMSTAR. The full list of questions is provided in Supplementary Table X.

| **Study** | **Q1** | **Q2** | **Q3** | **Q4** | **Q5** | **Q6** | **Q7** | **Q8** | **Q9** | **Q10** | **Q11** | **Total** |
| --- | --- | --- | --- | --- | --- | --- | --- | --- | --- | --- | --- | --- |
| Albano et al. (2019) | 3 | 1 | 3 | 2 | 1 | 3 | 3 | 1 | 3 | 3 | 3 | 26 |
| Aparicio et al. (2016) | 3 | 4 | 2 | 1 | 1 | 3 | 4 | 4 | 2 | 4 | 2 | 30 |
| Arafah et al. (2017) | 3 | 4 | 4 | 2 | 2 | 4 | 1 | 1 | 2 | 1 | 3 | 27 |
| Bacaltchuk et al. (2001) | 3 | 4 | 4 | 1 | 4 | 3 | 4 | 2 | 3 | 3 | 3 | 34 |
| Benbow et al. (2019) | 3 | 1 | 3 | 1 | 2 | 3 | 4 | 3 | 3 | 3 | 1 | 27 |
| Bevens et al. (2022) | 3 | 2 | 4 | 2 | 2 | 4 | 3 | 1 | 3 | 3 | 2 | 29 |
| Bighelli et al. (2018) | 4 | 2 | 3 | 2 | 1 | 4 | 4 | 1 | 3 | 3 | 3 | 30 |
| Bricca et al. (2022) | 4 | 4 | 4 | 1 | 1 | 1 | 4 | 1 | 1 | 1 | 3 | 25 |
| Chen et al. (2021) | 3 | 4 | 4 | 2 | 4 | 4 | 4 | 4 | 3 | 4 | 3 | 39 |
| Cooper et al. (2015) | 3 | 1 | 1 | 1 | 1 | 1 | 1 | 1 | 4 | 1 | 3 | 18 |
| Cramer et al. (2016) | 3 | 2 | 4 | 2 | 1 | 2 | 1 | 1 | 1 | 1 | 2 | 20 |
| Crutzen et al. (2015) | 3 | 4 | 2 | 1 | 1 | 4 | 1 | 1 | 3 | 3 | 1 | 24 |
| Cuijpers et al. (2009) | 2 | 1 | 2 | 3 | 1 | 4 | 4 | 2 | 4 | 3 | 2 | 28 |
| Cunill et al. (2013) | 4 | 2 | 3 | 1 | 2 | 3 | 4 | 2 | 4 | 4 | 2 | 31 |
| de Campos Moreira et al. (2017) | 4 | 2 | 4 | 3 | 2 | 4 | 4 | 4 | 4 | 1 | 2 | 34 |
| DeCrescenzo et al. (2018) | 4 | 4 | 4 | 3 | 2 | 4 | 4 | 1 | 2 | 3 | 3 | 34 |
| Dixon et al. (2020) | 4 | 4 | 4 | 1 | 2 | 3 | 3 | 2 | 3 | 2 | 3 | 31 |
| Doyle et al. (2021) | 4 | 4 | 4 | 2 | 3 | 4 | 4 | 4 | 1 | 1 | 2 | 33 |
| Dudas et al. (2018) | 3 | 4 | 4 | 2 | 2 | 4 | 4 | 4 | 4 | 4 | 3 | 38 |
| Edwards-Stewart et al. (2021) | 4 | 4 | 3 | 2 | 2 | 3 | 4 | 1 | 2 | 1 | 3 | 29 |
| Elsner et al. (2020) | 3 | 4 | 4 | 4 | 4 | 3 | 4 | 4 | 4 | 3 | 3 | 40 |
| Fabricatore et al. (2009) | 3 | 2 | 1 | 2 | 1 | 2 | 1 | 1 | 1 | 1 | 2 | 17 |
| Frampton et al. (2003) | 3 | 4 | 4 | 1 | 4 | 4 | 4 | 3 | 2 | 2 | 2 | 33 |
| Furukawa et al. (2020) | 3 | 4 | 3 | 4 | 2 | 4 | 2 | 1 | 2 | 1 | 2 | 28 |
| Gagliardi et al. (2019) | 3 | 1 | 4 | 2 | 4 | 4 | 4 | 4 | 3 | 2 | 2 | 33 |
| Gehling et al. (2011) | 3 | 2 | 4 | 1 | 3 | 2 | 2 | 1 | 3 | 1 | 1 | 23 |
| Goldberg et al. (2021) | 2 | 3 | 4 | 2 | 2 | 3 | 4 | 3 | 3 | 3 | 3 | 32 |
| Harris et al. (2021) | 4 | 4 | 4 | 2 | 2 | 3 | 4 | 1 | 3 | 2 | 2 | 31 |
| Heneghan et al. (2007) | 2 | 1 | 3 | 2 | 1 | 4 | 1 | 1 | 4 | 1 | 2 | 22 |
| Heo et al. (2009) | 3 | 4 | 2 | 2 | 1 | 4 | 1 | 1 | 1 | 2 | 2 | 23 |
| Hernandez-Rodriguez et al. (2022) | 4 | 1 | 3 | 2 | 4 | 3 | 4 | 4 | 3 | 4 | 2 | 34 |
| Hornyak et al. (2014) | 2 | 1 | 3 | 3 | 2 | 4 | 4 | 1 | 3 | 3 | 2 | 28 |
| Hrobjartsson et al. (2014) | 3 | 1 | 4 | 1 | 2 | 4 | 3 | 4 | 3 | 1 | 2 | 28 |
| Huang et al. (2014) | 3 | 4 | 4 | 2 | 2 | 3 | 4 | 1 | 3 | 2 | 2 | 30 |
| Ibrahim et al. (2016) | 3 | 1 | 4 | 2 | 2 | 4 | 1 | 1 | 1 | 1 | 3 | 23 |
| Iliakis et al. (2021) | 2 | 3 | 3 | 3 | 2 | 3 | 3 | 1 | 2 | 2 | 1 | 25 |
| Imel et al. (2013) | 3 | 4 | 3 | 1 | 1 | 1 | 1 | 1 | 2 | 1 | 2 | 20 |
| Jabardo-Camprubi et al. (2020) | 4 | 4 | 4 | 2 | 2 | 3 | 4 | 2 | 3 | 2 | 3 | 33 |
| Karyotaki et al. (2015) | 3 | 2 | 4 | 1 | 1 | 2 | 4 | 1 | 1 | 1 | 2 | 22 |
| Kato et al. (2021) | 3 | 1 | 3 | 1 | 2 | 4 | 3 | 1 | 1 | 4 | 3 | 26 |
| Kline et al. (2021) | 3 | 4 | 4 | 2 | 2 | 3 | 2 | 1 | 2 | 2 | 2 | 27 |
| Koog et al. (2013) | 3 | 3 | 4 | 2 | 1 | 1 | 1 | 1 | 2 | 1 | 1 | 20 |
| Kredo et al. (2013) | 3 | 2 | 4 | 4 | 4 | 3 | 4 | 3 | 4 | 1 | 3 | 35 |
| Lam et al. (2022) | 3 | 2 | 4 | 3 | 1 | 2 | 1 | 4 | 3 | 3 | 3 | 29 |
| Leucht et al. (2017) | 4 | 4 | 3 | 4 | 2 | 3 | 4 | 4 | 4 | 4 | 3 | 39 |
| Levinson et al. (2022) | 3 | 4 | 4 | 4 | 2 | 3 | 4 | 1 | 3 | 1 | 2 | 31 |
| Lewis et al. (2020) | 3 | 2 | 4 | 1 | 2 | 4 | 4 | 1 | 4 | 3 | 2 | 30 |
| Li et al. (2020) | 3 | 4 | 2 | 1 | 2 | 3 | 1 | 1 | 4 | 3 | 2 | 26 |
| Linardon et al. (2018) | 3 | 1 | 3 | 2 | 2 | 4 | 4 | 4 | 3 | 1 | 1 | 28 |
| Linardon et al. (2019) | 3 | 2 | 3 | 2 | 2 | 3 | 4 | 3 | 2 | 2 | 1 | 27 |
| Linardon et al. (2020) | 3 | 4 | 3 | 2 | 2 | 3 | 3 | 3 | 3 | 3 | 1 | 30 |
| Makatsori et al. (2014) | 3 | 4 | 3 | 1 | 1 | 4 | 1 | 1 | 2 | 1 | 1 | 22 |
| Martin et al. (2006) | 3 | 2 | 4 | 3 | 2 | 3 | 2 | 1 | 3 | 2 | 2 | 27 |
| Matsusaki et al. (2019) | 2 | 4 | 3 | 2 | 2 | 3 | 4 | 3 | 1 | 1 | 2 | 27 |
| McVay et al. (2023) | 3 | 4 | 4 | 1 | 1 | 4 | 1 | 1 | 1 | 1 | 2 | 23 |
| Minozzi et al. (2020) | 3 | 4 | 4 | 4 | 4 | 3 | 4 | 4 | 2 | 2 | 3 | 37 |
| Mitsikostas et al. (2012) | 3 | 1 | 2 | 2 | 2 | 3 | 1 | 1 | 2 | 3 | 2 | 22 |
| Miyasaka et al. (2006) | 3 | 4 | 4 | 3 | 4 | 3 | 4 | 3 | 4 | 2 | 3 | 37 |
| Ong et al. (2016) | 3 | 4 | 4 | 2 | 1 | 4 | 1 | 1 | 3 | 3 | 1 | 27 |
| Ong et al. (2018) | 3 | 4 | 2 | 2 | 2 | 4 | 3 | 1 | 3 | 4 | 2 | 30 |
| Palmowski et al. (2020) | 4 | 1 | 2 | 1 | 1 | 4 | 1 | 1 | 2 | 1 | 3 | 21 |
| Pampallona et al. (2004) | 1 | 4 | 4 | 1 | 3 | 4 | 3 | 1 | 3 | 2 | 2 | 28 |
| Papadopoulos et al. (2010) | 3 | 1 | 2 | 2 | 1 | 3 | 1 | 1 | 3 | 3 | 1 | 21 |
| Pozza et al. (2017) | 3 | 2 | 4 | 2 | 2 | 4 | 4 | 4 | 4 | 4 | 2 | 35 |
| Rabinowitz et al. (2009) | 2 | 1 | 3 | 1 | 2 | 4 | 1 | 1 | 1 | 1 | 2 | 19 |
| Reas et al. (2008) | 3 | 1 | 4 | 1 | 4 | 3 | 1 | 1 | 4 | 1 | 2 | 25 |
| Rehman et al. (2021) | 4 | 4 | 4 | 1 | 2 | 3 | 4 | 2 | 3 | 1 | 2 | 30 |
| Rutherford et al. (2013) | 3 | 4 | 4 | 2 | 2 | 4 | 1 | 1 | 3 | 2 | 2 | 28 |
| Schalkwijk et al. (2014) | 3 | 1 | 4 | 1 | 1 | 4 | 1 | 1 | 2 | 2 | 2 | 22 |
| Shah et al. (2020) | 3 | 1 | 1 | 2 | 2 | 2 | 4 | 1 | 2 | 1 | 3 | 22 |
| Somerson et al. (2016) | 3 | 4 | 3 | 1 | 1 | 4 | 1 | 1 | 1 | 1 | 2 | 22 |
| Song et al. (2021) | 4 | 4 | 4 | 2 | 2 | 3 | 2 | 4 | 1 | 1 | 3 | 30 |
| Stahl et al. (1993) | 2 | 2 | 3 | 2 | 2 | 1 | 3 | 1 | 3 | 1 | 1 | 21 |
| Stubbs et al. (2016) | 3 | 4 | 4 | 2 | 1 | 2 | 4 | 1 | 3 | 4 | 3 | 31 |
| Swift et al. (2017) | 3 | 4 | 2 | 2 | 1 | 1 | 1 | 1 | 4 | 1 | 1 | 21 |
| Szymczynska et al. (2017) | 3 | 1 | 4 | 2 | 2 | 4 | 4 | 1 | 4 | 2 | 1 | 28 |
| Tedeschini et al. (2010) | 3 | 1 | 4 | 1 | 1 | 1 | 1 | 1 | 3 | 1 | 2 | 19 |
| Torous et al. (2020) | 4 | 3 | 2 | 2 | 1 | 1 | 4 | 3 | 3 | 2 | 2 | 27 |
| Vancampfort et al. (2016) | 3 | 2 | 4 | 2 | 2 | 4 | 1 | 1 | 2 | 4 | 4 | 29 |
| Vancampfort et al. (2017) | 3 | 1 | 4 | 2 | 2 | 3 | 4 | 4 | 2 | 4 | 2 | 31 |
| Vancampfort et al. (2021) | 3 | 4 | 4 | 3 | 2 | 3 | 1 | 1 | 3 | 3 | 3 | 30 |
| Villeneuve et al. (2010) | 3 | 2 | 4 | 3 | 1 | 1 | 1 | 1 | 3 | 4 | 2 | 25 |
| Wahlbeck et al. (2001) | 1 | 2 | 1 | 2 | 1 | 4 | 1 | 1 | 1 | 2 | 1 | 17 |
| Wasmann et al. (2019) | 4 | 2 | 4 | 2 | 3 | 4 | 1 | 1 | 2 | 1 | 2 | 26 |
| Weinmann et al. (2008) | 2 | 2 | 3 | 1 | 2 | 3 | 1 | 1 | 3 | 3 | 1 | 22 |
| Windle et al. (2020) | 3 | 4 | 4 | 4 | 2 | 3 | 4 | 3 | 4 | 3 | 3 | 37 |
| Zhang et al. (2021) | 3 | 4 | 4 | 3 | 2 | 3 | 4 | 3 | 4 | 3 | 2 | 35 |
| Zhou et al. (2020) | 3 | 4 | 2 | 1 | 2 | 3 | 4 | 1 | 4 | 2 | 2 | 28 |

## Supplementary Figure 1. Frequency of R-AMSTAR total and domain scores. Total score is presented as a histogram. Domain scores are presented using bar charts.


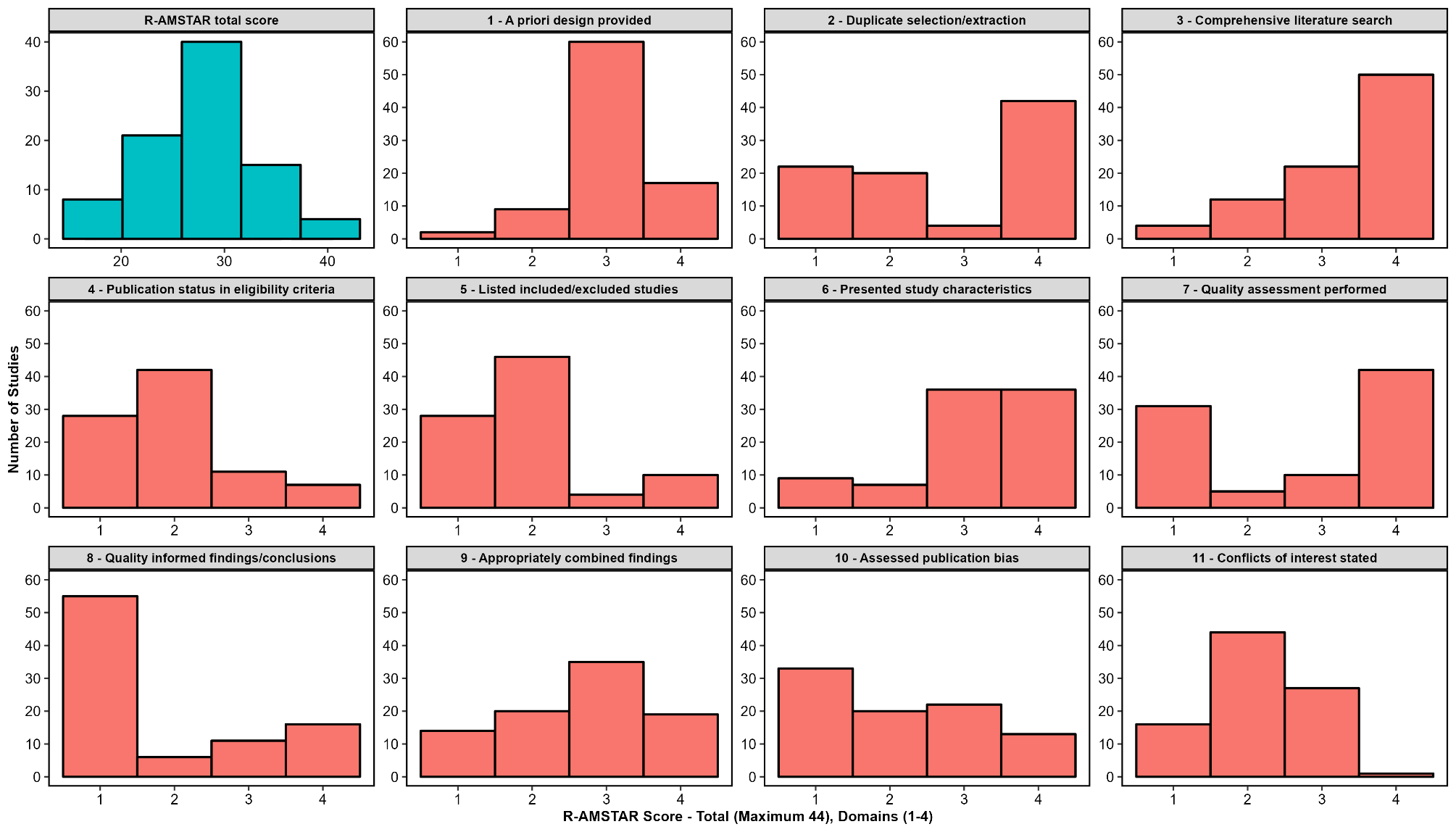


## Supplementary Table 10. Summary of participant characteristics evaluated by included reviews. Total number of reviews describes the number of reviews evaluating a participant characteristic described by a characteristic group among included reviews that evaluated participant characteristics (N/Included reviews that evaluated participant characteristics (%)). Number of reviews describes the number of reviews that studied a participant characteristic within a characteristic group (N/Total reviews in a characteristic group (%)). Number of reviews finding associations describes the number of reviews that found a participant characteristic to be associated with attrition among reviews evaluating the characteristic (N/Total reviews evaluating the participant characteristic (%)). Number of reviews not finding associations describes the number of reviews that did not find a participant characteristic to be associated with attrition among reviews evaluating the characteristic (N/Total reviews evaluating the participant characteristic (%)).

| **Participant characteristic group** | **Total number of reviews** | **Participant characteristic evaluated** | **Number of reviews** | **Number of reviews finding associations** | **Number of reviews not finding associations** |
| --- | --- | --- | --- | --- | --- |
| Comorbidity | 4/42 (9.52) | Presence of comorbidities | 3/4 (75) | 1/3 (33.33) | 2/3 (66.67) |
|  |  | Number of comorbidities | 1/4 (25) | 0/1 (0) | 1/1 (100) |
|  |  | Comorbidity severity | 1/4 (25) | 0/1 (0) | 1/1 (100) |
| Condition-related | 26/42 (61.9) | Index condition type | 13/26 (50) | 2/13 (15.38) | 11/13 (84.62) |
|  |  | Index condition severity | 10/26 (38.46) | 3/10 (30) | 7/10 (70) |
|  |  | Index condition duration | 5/26 (19.23) | 2/5 (40) | 3/5 (60) |
|  |  | Index condition symptoms | 3/26 (11.54) | 2/3 (66.67) | 1/3 (33.33) |
|  |  | Patient-caregiver relationship | 1/26 (3.85) | 1/1 (100) | 0/1 (0) |
|  |  | Lab measures | 1/26 (3.85) | 0/1 (0) | 1/1 (100) |
| Demographics | 34/42 (80.95) | Age | 33/34 (97.06) | 12/33 (36.36) | 21/33 (63.64) |
|  |  | Sex | 29/34 (85.29) | 5/29 (17.24) | 24/29 (82.76) |
|  |  | Race/Ethnicity | 6/34 (17.65) | 1/6 (16.67) | 5/6 (83.33) |
|  |  | BMI | 4/34 (11.76) | 2/4 (50) | 2/4 (50) |
|  |  | Relationship status | 4/34 (11.76) | 1/4 (25) | 3/4 (75) |
|  |  | Socioeconomic status | 6/34 (17.65) | 1/6 (16.67) | 5/6 (83.33) |
| Depression/Anxiety | 7/42 (16.67) | Depression | 4/7 (57.14) | 2/4 (50) | 2/4 (50) |
|  |  | Depression/Anxiety | 2/7 (28.57) | 0/2 (0) | 2/2 (100) |
|  |  | Anxiety | 1/7 (14.29) | 1/1 (100) | 0/1 (0) |
| Health status | 4/42 (9.52) | General health | 2/4 (50) | 2/2 (100) | 0/2 (0) |
|  |  | Health triggers | 1/4 (25) | 0/1 (0) | 1/1 (100) |
|  |  | Quality of Life | 1/4 (25) | 0/1 (0) | 1/1 (100) |
| Medical history | 6/42 (14.29) | Medications/Therapies used | 4/6 (66.67) | 0/4 (0) | 4/4 (100) |
|  |  | Ongoing conditions | 1/6 (16.67) | 1/1 (100) | 1/1 (100) |
|  |  | Hypertension | 1/6 (16.67) | 1/1 (100) | 0/1 (0) |
| Military experience | 3/42 (7.14) | Veteran/Military status | 2/3 (66.67) | 0/2 (0) | 2/2 (100) |
|  |  | Military history | 1/3 (33.33) | 0/1 (0) | 1/1 (100) |
| Perception/Motivation | 3/42 (7.14) | Motivation to quit | 1/3 (33.33) | 1/1 (100) | 0/1 (0) |
|  |  | Concerns about disorder | 1/3 (33.33) | 0/1 (0) | 1/1 (100) |
|  |  | Treatment experience | 1/3 (33.33) | 0/1 (0) | 1/1 (100) |
| Physical function | 3/42 (7.14) | Physical function | 1/3 (33.33) | 0/1 (0) | 1/1 (100) |
|  |  | Cardiorespiratory fitness | 1/3 (33.33) | 1/1 (100) | 0/1 (0) |
|  |  | Physical activity | 1/3 (33.33) | 1/1 (100) | 0/1 (0) |
| Substance use | 3/42 (7.14) | Smoking status | 2/3 (66.67) | 0/2 (0) | 2/2 (100) |
|  |  | Substance use | 1/3 (33.33) | 0/1 (0) | 1/1 (100) |
|  |  | Alcohol abuse | 1/3 (33.33) | 0/1 (0) | 1/1 (100) |

## Supplementary Table 11. Summary of participant characteristics evaluated among conditions studied. Number of reviews evaluating characteristic describes the number of reviews for a condition that evaluated a participant characteristic (N/Number of reviews for a condition (%)). Number of reviews finding associations describes the number of reviews for a condition that found a participant characteristic to be associated with attrition among reviews evaluating the characteristic (N/Total reviews for a condition evaluating the participant characteristic (%)). Number of reviews not finding associations describes the number of reviews for a condition that did not find a participant characteristic to be associated with attrition among reviews evaluating the characteristic (N/Total reviews for a condition evaluating the participant characteristic (%)).

| **Condition** | **Participant characteristic evaluated** | **Number of reviews evaluating characteristic** | **Number of reviews finding associations** | **Number of reviews not finding associations** |
| --- | --- | --- | --- | --- |
| Addiction (N=2) | Sex | 1/2 (50) | 0/1 (0) | 1/1 (100) |
|  | Age | 1/2 (50) | 0/1 (0) | 1/1 (100) |
|  | Medications/Therapies used | 1/2 (50) | 0/1 (0) | 1/1 (100) |
|  | Substance use | 1/2 (50) | 0/1 (0) | 1/1 (100) |
|  | Alcohol abuse | 1/2 (50) | 0/1 (0) | 1/1 (100) |
| Allergy (N=1) | Index condition type | 1/1 (100) | 0/1 (0) | 1/1 (100) |
|  | Age | 1/1 (100) | 0/1 (0) | 1/1 (100) |
| Anxiety (N=3) | Age | 1/3 (33.33) | 0/1 (0) | 1/1 (100) |
|  | Sex | 1/3 (33.33) | 0/1 (0) | 1/1 (100) |
|  | Index condition type | 1/3 (33.33) | 0/1 (0) | 1/1 (100) |
| Cancer (N=2) | Patient-caregiver relationship | 1/2 (50) | 1/1 (100) | 0/1 (0) |
|  | Index condition severity | 1/2 (50) | 0/1 (0) | 1/1 (100) |
|  | Age | 1/2 (50) | 0/1 (0) | 1/1 (100) |
|  | Sex | 1/2 (50) | 0/1 (0) | 1/1 (100) |
| Depression (N=15) | Sex | 7/15 (46.67) | 1/7 (14.29) | 6/7 (85.71) |
|  | Age | 7/15 (46.67) | 2/7 (28.57) | 5/7 (71.43) |
|  | Index condition severity | 4/15 (26.67) | 3/4 (75) | 1/4 (25) |
|  | Medications/Therapies used | 2/15 (13.33) | 0/2 (0) | 2/2 (100) |
|  | Index condition type | 2/15 (13.33) | 1/2 (50) | 1/2 (50) |
|  | Relationship status | 2/15 (13.33) | 1/2 (50) | 1/2 (50) |
|  | Index condition symptoms | 1/15 (6.67) | 1/1 (100) | 0/1 (0) |
|  | Presence of comorbidities | 1/15 (6.67) | 0/1 (0) | 1/1 (100) |
|  | Race/Ethnicity | 1/15 (6.67) | 1/1 (100) | 0/1 (0) |
|  | Socioeconomic status | 2/15 (13.33) | 1/2 (50) | 1/2 (50) |
|  | Anxiety | 1/15 (6.67) | 1/1 (100) | 0/1 (0) |
|  | Depression | 1/15 (6.67) | 1/1 (100) | 0/1 (0) |
| Eating disorders (N=4) | Index condition type | 1/4 (25) | 0/1 (0) | 1/1 (100) |
|  | Age | 1/4 (25) | 0/1 (0) | 1/1 (100) |
|  | Concerns about disorder | 1/4 (25) | 0/1 (0) | 1/1 (100) |
|  | Index condition severity | 1/4 (25) | 0/1 (0) | 1/1 (100) |
|  | Depression | 1/4 (25) | 0/1 (0) | 1/1 (100) |
| Fibromyalgia (N=1) | Depression | 1/1 (100) | 1/1 (100) | 0/1 (0) |
|  | Age | 1/1 (100) | 1/1 (100) | 0/1 (0) |
|  | Sex | 1/1 (100) | 0/1 (0) | 1/1 (100) |
|  | Race/Ethnicity | 1/1 (100) | 0/1 (0) | 1/1 (100) |
| HIV (N=2) | Sex | 1/2 (50) | 1/1 (100) | 0/1 (0) |
|  | BMI | 1/2 (50) | 1/1 (100) | 0/1 (0) |
|  | Cardiorespiratory fitness | 1/2 (50) | 1/1 (100) | 0/1 (0) |
|  | Age | 1/2 (50) | 0/1 (0) | 1/1 (100) |
|  | Index condition duration | 1/2 (50) | 0/1 (0) | 1/1 (100) |
|  | Socioeconomic status | 1/2 (50) | 0/1 (0) | 1/1 (100) |
|  | Race/Ethnicity | 1/2 (50) | 0/1 (0) | 1/1 (100) |
|  | Smoking status | 1/2 (50) | 0/1 (0) | 1/1 (100) |
|  | Lab measures | 1/2 (50) | 0/1 (0) | 1/1 (100) |
| Mixed conditions (N=5) | Age | 2/5 (40) | 1/2 (50) | 1/2 (50) |
|  | Sex | 2/5 (40) | 1/2 (50) | 1/2 (50) |
|  | General health | 1/5 (20) | 1/1 (100) | 0/1 (0) |
|  | Presence of comorbidities | 1/5 (20) | 1/1 (100) | 0/1 (0) |
| Mixed psychological disorders (N=8) | Age | 4/8 (50) | 2/4 (50) | 2/4 (50) |
|  | Sex | 3/8 (37.5) | 0/3 (0) | 3/3 (100) |
|  | Race/Ethnicity | 3/8 (37.5) | 0/3 (0) | 3/3 (100) |
|  | Index condition type | 3/8 (37.5) | 1/3 (33.33) | 2/3 (66.67) |
|  | Relationship status | 2/8 (25) | 0/2 (0) | 2/2 (100) |
|  | Depression/Anxiety | 1/8 (12.5) | 0/1 (0) | 1/1 (100) |
|  | Socioeconomic status | 1/8 (12.5) | 0/1 (0) | 1/1 (100) |
|  | Presence of comorbidities | 1/8 (12.5) | 0/1 (0) | 1/1 (100) |
|  | Veteran/Military status | 1/8 (12.5) | 0/1 (0) | 1/1 (100) |
| Multimorbidity (N=1) | Age | 1/1 (100) | 1/1 (100) | 0/1 (0) |
|  | Hypertension | 1/1 (100) | 1/1 (100) | 0/1 (0) |
|  | Sex | 1/1 (100) | 0/1 (0) | 1/1 (100) |
|  | BMI | 1/1 (100) | 0/1 (0) | 1/1 (100) |
|  | Socioeconomic status | 1/1 (100) | 0/1 (0) | 1/1 (100) |
|  | Index condition severity | 1/1 (100) | 0/1 (0) | 1/1 (100) |
|  | Number of comorbidities | 1/1 (100) | 0/1 (0) | 1/1 (100) |
|  | Comorbidity severity | 1/1 (100) | 0/1 (0) | 1/1 (100) |
|  | Physical function | 1/1 (100) | 0/1 (0) | 1/1 (100) |
|  | Quality of Life | 1/1 (100) | 0/1 (0) | 1/1 (100) |
|  | Depression/Anxiety | 1/1 (100) | 0/1 (0) | 1/1 (100) |
| Multiple sclerosis (N=3) | Index condition duration | 1/3 (33.33) | 1/1 (100) | 0/1 (0) |
|  | Age | 1/3 (33.33) | 1/1 (100) | 0/1 (0) |
|  | Sex | 1/3 (33.33) | 0/1 (0) | 1/1 (100) |
| OCD (N=2) | Index condition type | 1/2 (50) | 0/1 (0) | 1/1 (100) |
|  | Treatment experience | 1/2 (50) | 0/1 (0) | 1/1 (100) |
| Obesity (N=3) | Sex | 1/3 (33.33) | 1/1 (100) | 0/1 (0) |
|  | BMI | 1/3 (33.33) | 1/1 (100) | 0/1 (0) |
|  | Age | 1/3 (33.33) | 0/1 (0) | 1/1 (100) |
|  | Physical activity | 1/3 (33.33) | 1/1 (100) | 0/1 (0) |
| Orthopaedic surgery (N=1) | Age | 1/1 (100) | 0/1 (0) | 1/1 (100) |
| PTSD (N=6) | Index condition symptoms | 2/6 (33.33) | 1/2 (50) | 1/2 (50) |
|  | Sex | 2/6 (33.33) | 0/2 (0) | 2/2 (100) |
|  | Age | 1/6 (16.67) | 0/1 (0) | 1/1 (100) |
|  | Military history | 1/6 (16.67) | 0/1 (0) | 1/1 (100) |
|  | Depression | 1/6 (16.67) | 0/1 (0) | 1/1 (100) |
|  | Veteran/Military status | 1/6 (16.67) | 0/1 (0) | 1/1 (100) |
|  | Index condition type | 1/6 (16.67) | 0/1 (0) | 1/1 (100) |
|  | Socioeconomic status | 1/6 (16.67) | 0/1 (0) | 1/1 (100) |
| Rheumatoid arthritis (N=2) | Age | 1/2 (50) | 1/1 (100) | 0/1 (0) |
|  | Index condition type | 1/2 (50) | 0/1 (0) | 1/1 (100) |
|  | Sex | 1/2 (50) | 0/1 (0) | 1/1 (100) |
| Schizophrenia (N=9) | Age | 4/9 (44.44) | 2/4 (50) | 2/4 (50) |
|  | Sex | 4/9 (44.44) | 0/4 (0) | 4/4 (100) |
|  | Index condition severity | 3/9 (33.33) | 0/3 (0) | 3/3 (100) |
|  | Index condition duration | 3/9 (33.33) | 1/3 (33.33) | 2/3 (66.67) |
|  | Index condition type | 1/9 (11.11) | 0/1 (0) | 1/1 (100) |
|  | BMI | 1/9 (11.11) | 0/1 (0) | 1/1 (100) |
|  | Medications/Therapies used | 1/9 (11.11) | 0/1 (0) | 1/1 (100) |
| Smoking cessation (N=2) | Ongoing conditions | 2/2 (100) | 1/2 (50) | 1/2 (50) |
|  | Sex | 1/2 (50) | 1/1 (100) | 0/1 (0) |
|  | Motivation to quit | 1/2 (50) | 1/1 (100) | 0/1 (0) |
|  | Age | 1/2 (50) | 0/1 (0) | 1/1 (100) |
|  | Socioeconomic status | 1/2 (50) | 0/1 (0) | 1/1 (100) |
|  | Smoking | 1/2 (50) | 0/1 (0) | 1/1 (100) |
|  | Health triggers | 1/2 (50) | 0/1 (0) | 1/1 (100) |
| Type 2 diabetes (N=2) | Age | 2/2 (100) | 1/2 (50) | 1/2 (50) |
|  | Index condition type | 1/2 (50) | 0/1 (0) | 1/1 (100) |
|  | Sex | 1/2 (50) | 0/1 (0) | 1/1 (100) |
| Vocal rehabilitation (N=1) | General health | 1/1 (100) | 1/1 (100) | 0/1 (0) |

## Supplementary Table 12. Summary of trial characteristics evaluated by included reviews. Total number of reviews describes the number of reviews evaluating a trial characteristic described by a characteristic group among included reviews that evaluated trial characteristics (N/Included reviews that evaluated trial characteristics (%)). Number of reviews describes the number of reviews that studied a trial characteristic within a characteristic group (N/Total reviews in a characteristic group (%)). Number of reviews finding associations describes the number of reviews that found a trial characteristic to be associated with attrition among reviews evaluating the characteristic (N/Total reviews evaluating the trial characteristic (%)). Number of reviews not finding associations describes the number of reviews that did not find a trial characteristic to be associated with attrition among reviews evaluating the characteristic (N/Total reviews evaluating the trial characteristic (%)).

| **Trial characteristic group** | **Total number of reviews** | **Trial characteristic evaluated** | **Number of reviews** | **Number of reviews finding associations** | **Number of reviews not finding associations** |
| --- | --- | --- | --- | --- | --- |
| Eligibility criteria | 16/87 (18.39) | Index condition type | 8/16 (50) | 1/8 (12.5) | 7/8 (87.5) |
|  |  | Comorbidities | 4/16 (25) | 1/4 (25) | 3/4 (75) |
|  |  | Eligibility criteria | 1/16 (6.25) | 0/1 (0) | 1/1 (100) |
|  |  | Age limit | 1/16 (6.25) | 0/1 (0) | 1/1 (100) |
|  |  | Exclusions based on comorbidities | 1/16 (6.25) | 0/1 (0) | 1/1 (100) |
|  |  | Medication restrictions | 1/16 (6.25) | 1/1 (100) | 0/1 (0) |
|  |  | Index condition severity | 1/16 (6.25) | 0/1 (0) | 1/1 (100) |
| Enrolment-related | 2/87 (2.3) | Enrolment method | 2/2 (100) | 1/2 (50) | 1/2 (50) |
|  |  | Enrolment setting | 1/2 (50) | 0/1 (0) | 1/1 (100) |
| Follow-up related | 5/87 (5.75) | Follow-up duration | 4/5 (80) | 3/4 (75) | 1/4 (25) |
|  |  | Follow-up frequency/intensity | 2/5 (40) | 0/2 (0) | 2/2 (100) |
|  |  | Follow-up strategy | 1/5 (20) | 1/1 (100) | 0/1 (0) |
| Intervention-related | 79/87 (90.8) | Intervention type | 56/79 (70.89) | 29/56 (51.79) | 27/56 (48.21) |
|  |  | Intervention frequency/intensity | 29/79 (36.71) | 7/29 (24.14) | 22/29 (75.86) |
|  |  | Intervention delivery/format | 26/79 (32.91) | 6/26 (23.08) | 20/26 (76.92) |
|  |  | Intervention duration | 14/79 (17.72) | 6/14 (42.86) | 8/14 (57.14) |
|  |  | Experimental intervention type | 11/79 (13.92) | 5/11 (45.45) | 6/11 (54.55) |
|  |  | Intervention components | 9/79 (11.39) | 4/9 (44.44) | 5/9 (55.56) |
|  |  | Control/Comparator intervention type | 9/79 (11.39) | 4/9 (44.44) | 5/9 (55.56) |
|  |  | Intervention supervision | 5/79 (6.33) | 3/5 (60) | 2/5 (40) |
|  |  | Motivational/Incentive strategies | 2/79 (2.53) | 1/2 (50) | 1/2 (50) |
|  |  | Pharmacological support | 1/79 (1.27) | 0/1 (0) | 1/1 (100) |
|  |  | Number of components | 1/79 (1.27) | 0/1 (0) | 1/1 (100) |
|  |  | Clinician telephone crisis coaching | 1/79 (1.27) | 1/1 (100) | 0/1 (0) |
|  |  | Intervention focus | 1/79 (1.27) | 1/1 (100) | 0/1 (0) |
| Motivational/Incentive strategies | 5/87 (5.75) | Using incentives | 4/5 (80) | 2/4 (50) | 2/4 (50) |
|  |  | Use of motivational strategies | 1/5 (20) | 1/1 (100) | 0/1 (0) |
| Outcome measures | 5/87 (5.75) | Participant engagement | 1/5 (20) | 1/1 (100) | 0/1 (0) |
|  |  | Biomarker assessments | 1/5 (20) | 0/1 (0) | 1/1 (100) |
|  |  | Primary endpoint | 1/5 (20) | 0/1 (0) | 1/1 (100) |
|  |  | Treatment adherence | 1/5 (20) | 0/1 (0) | 1/1 (100) |
|  |  | Response rate | 1/5 (20) | 0/1 (0) | 1/1 (100) |
|  |  | Adverse event frequency | 1/5 (20) | 1/1 (100) | 0/1 (0) |
| Preference/Tailoring | 3/87 (3.45) | Preference/Tailoring | 3/3 (100) | 2/3 (66.67) | 1/3 (33.33) |
| Publication details | 17/87 (19.54) | Publication/Reporting year | 15/17 (88.24) | 8/15 (53.33) | 7/15 (46.67) |
|  |  | Publication type | 1/17 (5.88) | 0/1 (0) | 1/1 (100) |
|  |  | Impact factor | 1/17 (5.88) | 0/1 (0) | 1/1 (100) |
| Quality/Risk of bias | 17/87 (19.54) | Trial quality | 7/17 (41.18) | 2/7 (28.57) | 5/7 (71.43) |
|  |  | Methodological quality | 4/17 (23.53) | 0/4 (0) | 4/4 (100) |
|  |  | Risk of bias | 3/17 (17.65) | 0/3 (0) | 3/3 (100) |
|  |  | Dropout definition | 3/17 (17.65) | 1/3 (33.33) | 2/3 (66.67) |
|  |  | Reporting method | 1/17 (5.88) | 0/1 (0) | 1/1 (100) |
|  |  | Funding sources | 1/17 (5.88) | 1/1 (100) | 0/1 (0) |
| Recruitment-related | 5/87 (5.75) | Recruitment strategies | 4/5 (80) | 1/4 (25) | 3/4 (75) |
|  |  | Recruitment setting | 1/5 (20) | 1/1 (100) | 0/1 (0) |
| Trial conduct | 14/87 (16.09) | Clinician qualifications/training | 10/14 (71.43) | 6/10 (60) | 4/10 (40) |
|  |  | Presence of consultation team | 1/14 (7.14) | 0/1 (0) | 1/1 (100) |
|  |  | Involvement of treatment originator | 1/14 (7.14) | 0/1 (0) | 1/1 (100) |
|  |  | Clinician-Consultant meetings | 1/14 (7.14) | 1/1 (100) | 0/1 (0) |
|  |  | Clinician protocol adherence | 1/14 (7.14) | 0/1 (0) | 1/1 (100) |
|  |  | Randomisation rate | 1/14 (7.14) | 0/1 (0) | 1/1 (100) |
|  |  | Placebo randomisation | 1/14 (7.14) | 0/1 (0) | 1/1 (100) |
| Trial design | 47/87 (54.02) | Trial duration | 16/47 (34.04) | 9/16 (56.25) | 7/16 (43.75) |
|  |  | Sample size | 15/47 (31.91) | 5/15 (33.33) | 10/15 (66.67) |
|  |  | Trial location(s) | 14/47 (29.79) | 4/14 (28.57) | 10/14 (71.43) |
|  |  | Trial design | 12/47 (25.53) | 5/12 (41.67) | 7/12 (58.33) |
|  |  | Population studied | 9/47 (19.15) | 1/9 (11.11) | 8/9 (88.89) |
|  |  | Use of run-in period | 6/47 (12.77) | 3/6 (50) | 3/6 (50) |
|  |  | Setting | 5/47 (10.64) | 3/5 (60) | 2/5 (40) |
|  |  | Number of arms | 4/47 (8.51) | 1/4 (25) | 3/4 (75) |
|  |  | Number of sites | 4/47 (8.51) | 3/4 (75) | 1/4 (25) |
|  |  | Visit frequency | 3/47 (6.38) | 1/3 (33.33) | 2/3 (66.67) |
|  |  | Use of retention strategies | 1/47 (2.13) | 0/1 (0) | 1/1 (100) |
|  |  | Use of reminders | 1/47 (2.13) | 1/1 (100) | 0/1 (0) |
|  |  | Trial phase | 1/47 (2.13) | 1/1 (100) | 0/1 (0) |
|  |  | Open label design | 1/47 (2.13) | 0/1 (0) | 1/1 (100) |
|  |  | Placebo treatment arm | 1/47 (2.13) | 1/1 (100) | 0/1 (0) |
|  |  | Blinding | 1/47 (2.13) | 1/1 (100) | 0/1 (0) |
|  |  | Crossover design | 1/47 (2.13) | 1/1 (100) | 0/1 (0) |

## Supplementary Table 13. Summary of trial characteristics evaluated among conditions studied. Number of reviews evaluating characteristic describes the number of reviews for a condition that evaluated a trial characteristic (N/Number of reviews for a condition (%)). Number of reviews finding associations describes the number of reviews for a condition that found a trial characteristic to be associated with attrition among reviews evaluating the characteristic (N/Total reviews for a condition evaluating the trial characteristic (%)). Number of reviews not finding associations describes the number of reviews for a condition that did not find a trial characteristic to be associated with attrition among reviews evaluating the characteristic (N/Total reviews for a condition evaluating the trial characteristic (%)).

| **Condition** | **Trial characteristic name on heat map** | **Trial characteristic evaluated** | **Number of reviews evaluating characteristic** | **Number of reviews finding associations** | **Number of reviews not finding associations** |
| --- | --- | --- | --- | --- | --- |
| ADHD (N=1) | Intervention duration | Intervention duration | 1/1 (100) | 0/1 (0) | 1/1 (100) |
|  | Intervention frequency/intensity | Intervention frequency/intensity | 1/1 (100) | 0/1 (0) | 1/1 (100) |
|  | Eligibility \| Comorbidities | Comorbidities | 1/1 (100) | 0/1 (0) | 1/1 (100) |
|  | Trial design \| Use of run-in | Use of run-in period | 1/1 (100) | 0/1 (0) | 1/1 (100) |
|  | Recruitment-related | Recruitment strategies | 1/1 (100) | 0/1 (0) | 1/1 (100) |
| Acute mania (N=1) | Intervention type | Intervention type | 1/1 (100) | 0/1 (0) | 1/1 (100) |
| Addiction (N=2) | Intervention type | Intervention type | 2/2 (100) | 1/2 (50) | 1/2 (50) |
|  | Intervention frequency/intensity | Intervention frequency/intensity | 1/2 (50) | 0/1 (0) | 1/1 (100) |
|  | Publication Type/Year | Publication/Reporting year | 1/2 (50) | 0/1 (0) | 1/1 (100) |
|  | Trial design \| Sample size | Sample size | 1/2 (50) | 0/1 (0) | 1/1 (100) |
| Allergy (N=1) | Trial design \| Duration | Trial duration | 1/1 (100) | 0/1 (0) | 1/1 (100) |
|  | Intervention delivery/format | Intervention delivery/format | 1/1 (100) | 0/1 (0) | 1/1 (100) |
|  | Intervention frequency/intensity | Intervention frequency/intensity | 1/1 (100) | 0/1 (0) | 1/1 (100) |
|  | Trial design \| Number of sites | Number of sites | 1/1 (100) | 0/1 (0) | 1/1 (100) |
|  | Trial design \| Location(s) | Trial location(s) | 1/1 (100) | 0/1 (0) | 1/1 (100) |
| Anxiety (N=3) | Intervention frequency/intensity | Intervention frequency/intensity | 3/3 (100) | 1/3 (33.33) | 2/3 (66.67) |
|  | Intervention type | Intervention type | 2/3 (66.67) | 0/2 (0) | 2/2 (100) |
|  | Intervention components | Intervention components | 2/3 (66.67) | 1/2 (50) | 1/2 (50) |
|  | Trial design \| Setting | Setting | 1/3 (33.33) | 1/1 (100) | 0/1 (0) |
|  | Intervention duration | Intervention duration | 1/3 (33.33) | 1/1 (100) | 0/1 (0) |
|  | Intervention supervision | Intervention supervision | 1/3 (33.33) | 1/1 (100) | 0/1 (0) |
|  | Clinician qualifications/training | Clinician qualifications/training | 1/3 (33.33) | 1/1 (100) | 0/1 (0) |
|  | Motivational/Incentive strategies | Use of motivational strategies | 1/3 (33.33) | 1/1 (100) | 0/1 (0) |
|  | Eligibility \| Index condition | Index condition type | 1/3 (33.33) | 0/1 (0) | 1/1 (100) |
|  | Intervention type \| Experimental | Experimental intervention type | 1/3 (33.33) | 0/1 (0) | 1/1 (100) |
| Cancer (N=2) | Intervention duration | Intervention duration | 2/2 (100) | 1/2 (50) | 1/2 (50) |
|  | Trial design | Trial design | 1/2 (50) | 1/1 (100) | 0/1 (0) |
|  | Intervention delivery/format | Intervention delivery/format | 1/2 (50) | 1/1 (100) | 0/1 (0) |
|  | Follow-up related | Follow-up duration | 1/2 (50) | 1/1 (100) | 0/1 (0) |
|  | Trial design \| Number of arms | Number of arms | 1/2 (50) | 0/1 (0) | 1/1 (100) |
|  | Enrolment-related | Enrolment setting | 1/2 (50) | 0/1 (0) | 1/1 (100) |
|  | Enrolment-related | Enrolment method | 1/2 (50) | 0/1 (0) | 1/1 (100) |
|  | Intervention type | Intervention type | 1/2 (50) | 0/1 (0) | 1/1 (100) |
|  | Trial design \| Sample size | Sample size | 1/2 (50) | 0/1 (0) | 1/1 (100) |
| Chronic pain (N=1) | Intervention type | Intervention type | 1/1 (100) | 0/1 (0) | 1/1 (100) |
| Coronary artery disease (N=1) | Intervention type | Intervention type | 1/1 (100) | 0/1 (0) | 1/1 (100) |
| Dementia (N=2) | Intervention type | Intervention type | 2/2 (100) | 2/2 (100) | 0/2 (0) |
| Depression (N=15) | Intervention type | Intervention type | 11/15 (73.33) | 6/11 (54.55) | 5/11 (45.45) |
|  | Trial design \| Duration | Trial duration | 7/15 (46.67) | 3/7 (42.86) | 4/7 (57.14) |
|  | Publication Type/Year | Publication/Reporting year | 6/15 (40) | 5/6 (83.33) | 1/6 (16.67) |
|  | Trial design \| Population | Population studied | 5/15 (33.33) | 1/5 (20) | 4/5 (80) |
|  | Intervention delivery/format | Intervention delivery/format | 4/15 (26.67) | 0/4 (0) | 4/4 (100) |
|  | Trial design \| Location(s) | Trial location(s) | 4/15 (26.67) | 2/4 (50) | 2/4 (50) |
|  | Intervention frequency/intensity | Intervention frequency/intensity | 4/15 (26.67) | 0/4 (0) | 4/4 (100) |
|  | Trial design \| Sample size | Sample size | 3/15 (20) | 2/3 (66.67) | 1/3 (33.33) |
|  | Intervention duration | Intervention duration | 3/15 (20) | 1/3 (33.33) | 2/3 (66.67) |
|  | Intervention type \| Experimental | Experimental intervention type | 3/15 (20) | 2/3 (66.67) | 1/3 (33.33) |
|  | Trial design \| Use of run-in | Use of run-in period | 2/15 (13.33) | 1/2 (50) | 1/2 (50) |
|  | Trial design \| Number of sites | Number of sites | 2/15 (13.33) | 2/2 (100) | 0/2 (0) |
|  | Trial design \| Number of arms | Number of arms | 2/15 (13.33) | 1/2 (50) | 1/2 (50) |
|  | Trial design \| Visit frequency | Visit frequency | 2/15 (13.33) | 1/2 (50) | 1/2 (50) |
|  | Clinician qualifications/training | Clinician qualifications/training | 2/15 (13.33) | 1/2 (50) | 1/2 (50) |
|  | Quality/Risk of bias | Trial quality | 2/15 (13.33) | 0/2 (0) | 2/2 (100) |
|  | Eligibility \| Index condition | Index condition type | 2/15 (13.33) | 0/2 (0) | 2/2 (100) |
|  | Intervention components | Intervention components | 1/15 (6.67) | 1/1 (100) | 0/1 (0) |
|  | Eligibility criteria | Eligibility criteria | 1/15 (6.67) | 0/1 (0) | 1/1 (100) |
|  | Motivational/Incentive strategies | Using incentives | 1/15 (6.67) | 0/1 (0) | 1/1 (100) |
|  | Outcome \| Engagement/Response | Response rate | 1/15 (6.67) | 0/1 (0) | 1/1 (100) |
|  | Trial design \| Setting | Setting | 1/15 (6.67) | 1/1 (100) | 0/1 (0) |
|  | Publication Type/Year | Publication type | 1/15 (6.67) | 0/1 (0) | 1/1 (100) |
|  | Intervention supervision | Intervention supervision | 1/15 (6.67) | 0/1 (0) | 1/1 (100) |
|  | Trial design | Trial design | 1/15 (6.67) | 0/1 (0) | 1/1 (100) |
|  | Trial conduct | Placebo randomisation | 1/15 (6.67) | 0/1 (0) | 1/1 (100) |
|  | Recruitment-related | Recruitment strategies | 1/15 (6.67) | 0/1 (0) | 1/1 (100) |
|  | Quality/Risk of bias | Methodological quality | 1/15 (6.67) | 0/1 (0) | 1/1 (100) |
| Eating disorders (N=4) | Intervention type | Intervention type | 3/4 (75) | 2/3 (66.67) | 1/3 (33.33) |
|  | Intervention delivery/format | Intervention delivery/format | 1/4 (25) | 1/1 (100) | 0/1 (0) |
|  | Intervention frequency/intensity | Intervention frequency/intensity | 1/4 (25) | 1/1 (100) | 0/1 (0) |
|  | Quality/Risk of bias | Trial quality | 1/4 (25) | 0/1 (0) | 1/1 (100) |
|  | Dropout definition | Dropout definition | 1/4 (25) | 0/1 (0) | 1/1 (100) |
|  | Intervention type \| Control/Comparator | Control/Comparator intervention type | 1/4 (25) | 1/1 (100) | 0/1 (0) |
| Fibromyalgia (N=1) | Intervention type | Intervention type | 1/1 (100) | 1/1 (100) | 0/1 (0) |
|  | Outcome \| Adverse event frequency | Adverse event frequency | 1/1 (100) | 1/1 (100) | 0/1 (0) |
|  | Intervention type \| Control/Comparator | Control/Comparator intervention type | 1/1 (100) | 1/1 (100) | 0/1 (0) |
|  | Trial design \| Sample size | Sample size | 1/1 (100) | 1/1 (100) | 0/1 (0) |
|  | Publication Type/Year | Publication/Reporting year | 1/1 (100) | 0/1 (0) | 1/1 (100) |
|  | Quality/Risk of bias | Methodological quality | 1/1 (100) | 0/1 (0) | 1/1 (100) |
| HIV (N=2) | Intervention delivery/format | Intervention delivery/format | 2/2 (100) | 0/2 (0) | 2/2 (100) |
|  | Intervention type | Intervention type | 1/2 (50) | 1/1 (100) | 0/1 (0) |
|  | Intervention supervision | Intervention supervision | 1/2 (50) | 1/1 (100) | 0/1 (0) |
|  | Clinician qualifications/training | Clinician qualifications/training | 1/2 (50) | 1/1 (100) | 0/1 (0) |
|  | Trial design \| Setting | Setting | 1/2 (50) | 0/1 (0) | 1/1 (100) |
|  | Motivational/Incentive strategies | Motivational/Incentive strategies | 1/2 (50) | 0/1 (0) | 1/1 (100) |
|  | Intervention frequency/intensity | Intervention frequency/intensity | 1/2 (50) | 0/1 (0) | 1/1 (100) |
| Herpes zoster (N=1) | Intervention type | Intervention type | 1/1 (100) | 0/1 (0) | 1/1 (100) |
| Knee osteoarthritis (N=1) | Intervention delivery/format | Intervention delivery/format | 1/1 (100) | 1/1 (100) | 0/1 (0) |
|  | Trial design \| Duration | Trial duration | 1/1 (100) | 1/1 (100) | 0/1 (0) |
|  | Trial design | Trial design | 1/1 (100) | 1/1 (100) | 0/1 (0) |
|  | Eligibility criteria | Medication restrictions | 1/1 (100) | 1/1 (100) | 0/1 (0) |
|  | Intervention type | Intervention type | 1/1 (100) | 0/1 (0) | 1/1 (100) |
| Mixed conditions (N=5) | Intervention type | Intervention type | 3/5 (60) | 2/3 (66.67) | 1/3 (33.33) |
|  | Intervention frequency/intensity | Intervention frequency/intensity | 2/5 (40) | 0/2 (0) | 2/2 (100) |
|  | Intervention duration | Intervention duration | 2/5 (40) | 1/2 (50) | 1/2 (50) |
|  | Intervention delivery/format | Intervention delivery/format | 1/5 (20) | 0/1 (0) | 1/1 (100) |
|  | Intervention type \| Control/Comparator | Control/Comparator intervention type | 1/5 (20) | 0/1 (0) | 1/1 (100) |
|  | Follow-up related | Follow-up frequency/intensity | 1/5 (20) | 0/1 (0) | 1/1 (100) |
|  | Follow-up related | Follow-up duration | 1/5 (20) | 0/1 (0) | 1/1 (100) |
|  | Eligibility \| Comorbidities | Comorbidities | 1/5 (20) | 0/1 (0) | 1/1 (100) |
|  | Trial design \| Sample size | Sample size | 1/5 (20) | 0/1 (0) | 1/1 (100) |
|  | Intervention type \| Experimental | Experimental intervention type | 1/5 (20) | 0/1 (0) | 1/1 (100) |
|  | Intervention components | Intervention components | 1/5 (20) | 1/1 (100) | 0/1 (0) |
|  | Trial design \| Location(s) | Trial location(s) | 1/5 (20) | 0/1 (0) | 1/1 (100) |
|  | Trial design \| Blinding | Blinding | 1/5 (20) | 1/1 (100) | 0/1 (0) |
|  | Preference/Tailoring | Preference/Tailoring | 1/5 (20) | 1/1 (100) | 0/1 (0) |
|  | Trial design \| Use of crossover | Crossover design | 1/5 (20) | 1/1 (100) | 0/1 (0) |
| Mixed psychological disorders (N=8) | Intervention type | Intervention type | 5/8 (62.5) | 3/5 (60) | 2/5 (40) |
|  | Intervention delivery/format | Intervention delivery/format | 5/8 (62.5) | 1/5 (20) | 4/5 (80) |
|  | Eligibility \| Index condition | Index condition type | 3/8 (37.5) | 1/3 (33.33) | 2/3 (66.67) |
|  | Intervention components | Intervention components | 3/8 (37.5) | 1/3 (33.33) | 2/3 (66.67) |
|  | Clinician qualifications/training | Clinician qualifications/training | 3/8 (37.5) | 2/3 (66.67) | 1/3 (33.33) |
|  | Intervention frequency/intensity | Intervention frequency/intensity | 3/8 (37.5) | 0/3 (0) | 3/3 (100) |
|  | Trial design \| Population | Population studied | 2/8 (25) | 0/2 (0) | 2/2 (100) |
|  | Dropout definition | Dropout definition | 2/8 (25) | 1/2 (50) | 1/2 (50) |
|  | Trial design \| Sample size | Sample size | 1/8 (12.5) | 1/1 (100) | 0/1 (0) |
|  | Preference/Tailoring | Preference/Tailoring | 1/8 (12.5) | 1/1 (100) | 0/1 (0) |
|  | Motivational/Incentive strategies | Using incentives | 1/8 (12.5) | 1/1 (100) | 0/1 (0) |
|  | Trial design \| Retention strategies | Use of reminders | 1/8 (12.5) | 1/1 (100) | 0/1 (0) |
|  | Enrolment-related | Enrolment method | 1/8 (12.5) | 1/1 (100) | 0/1 (0) |
|  | Trial conduct | Clinician-Consultant meetings | 1/8 (12.5) | 1/1 (100) | 0/1 (0) |
|  | Trial conduct | Clinician telephone crisis coaching | 1/8 (12.5) | 1/1 (100) | 0/1 (0) |
|  | Trial conduct | Clinician protocol adherence | 1/8 (12.5) | 0/1 (0) | 1/1 (100) |
|  | Eligibility \| Comorbidities | Exclusions based on comorbidities | 1/8 (12.5) | 0/1 (0) | 1/1 (100) |
|  | Quality/Risk of bias | Methodological quality | 1/8 (12.5) | 0/1 (0) | 1/1 (100) |
|  | Recruitment-related | Recruitment setting | 1/8 (12.5) | 1/1 (100) | 0/1 (0) |
|  | Intervention type \| Experimental | Experimental intervention type | 1/8 (12.5) | 0/1 (0) | 1/1 (100) |
|  | Intervention type \| Control/Comparator | Control/Comparator intervention type | 1/8 (12.5) | 0/1 (0) | 1/1 (100) |
|  | Publication Type/Year | Publication/Reporting year | 1/8 (12.5) | 0/1 (0) | 1/1 (100) |
|  | Trial design \| Location(s) | Trial location(s) | 1/8 (12.5) | 0/1 (0) | 1/1 (100) |
| Multimorbidity (N=1) | Intervention components | Intervention components | 1/1 (100) | 0/1 (0) | 1/1 (100) |
|  | Intervention type | Intervention type | 1/1 (100) | 0/1 (0) | 1/1 (100) |
|  | Intervention frequency/intensity | Intervention frequency/intensity | 1/1 (100) | 0/1 (0) | 1/1 (100) |
|  | Trial design \| Setting | Setting | 1/1 (100) | 0/1 (0) | 1/1 (100) |
|  | Intervention delivery/format | Intervention delivery/format | 1/1 (100) | 0/1 (0) | 1/1 (100) |
|  | Trial design | Trial design | 1/1 (100) | 0/1 (0) | 1/1 (100) |
|  | Intervention duration | Intervention duration | 1/1 (100) | 0/1 (0) | 1/1 (100) |
|  | Intervention supervision | Intervention supervision | 1/1 (100) | 0/1 (0) | 1/1 (100) |
|  | Preference/Tailoring | Preference/Tailoring | 1/1 (100) | 0/1 (0) | 1/1 (100) |
|  | Outcome \| Treatment adherence | Treatment adherence | 1/1 (100) | 0/1 (0) | 1/1 (100) |
| Multiple sclerosis (N=3) | Publication Type/Year | Publication/Reporting year | 2/3 (66.67) | 1/2 (50) | 1/2 (50) |
|  | Intervention frequency/intensity | Intervention frequency/intensity | 2/3 (66.67) | 1/2 (50) | 1/2 (50) |
|  | Outcome \| Engagement/Response | Participant engagement | 1/3 (33.33) | 1/1 (100) | 0/1 (0) |
|  | Trial design \| Sample size | Sample size | 1/3 (33.33) | 0/1 (0) | 1/1 (100) |
|  | Intervention components | Intervention components | 1/3 (33.33) | 0/1 (0) | 1/1 (100) |
|  | Quality/Risk of bias | Risk of bias | 1/3 (33.33) | 0/1 (0) | 1/1 (100) |
|  | Trial design | Trial design | 1/3 (33.33) | 1/1 (100) | 0/1 (0) |
|  | Trial design \| Phase | Trial phase | 1/3 (33.33) | 1/1 (100) | 0/1 (0) |
|  | Intervention type | Intervention type | 1/3 (33.33) | 0/1 (0) | 1/1 (100) |
|  | Trial design \| Population | Population studied | 1/3 (33.33) | 0/1 (0) | 1/1 (100) |
|  | Intervention delivery/format | Intervention delivery/format | 1/3 (33.33) | 0/1 (0) | 1/1 (100) |
|  | Intervention duration | Intervention duration | 1/3 (33.33) | 0/1 (0) | 1/1 (100) |
| OCD (N=2) | Intervention type | Intervention type | 1/2 (50) | 0/1 (0) | 1/1 (100) |
|  | Clinician qualifications/training | Clinician qualifications/training | 1/2 (50) | 0/1 (0) | 1/1 (100) |
|  | Intervention frequency/intensity | Intervention frequency/intensity | 1/2 (50) | 0/1 (0) | 1/1 (100) |
| Obesity (N=3) | Intervention type | Intervention type | 2/3 (66.67) | 1/2 (50) | 1/2 (50) |
|  | Trial design \| Use of run-in | Use of run-in period | 2/3 (66.67) | 2/2 (100) | 0/2 (0) |
|  | Eligibility \| Comorbidities | Comorbidities | 1/3 (33.33) | 1/1 (100) | 0/1 (0) |
|  | Trial design \| Visit frequency | Visit frequency | 1/3 (33.33) | 0/1 (0) | 1/1 (100) |
|  | Trial design \| Location(s) | Trial location(s) | 1/3 (33.33) | 0/1 (0) | 1/1 (100) |
|  | Trial design | Trial design | 1/3 (33.33) | 1/1 (100) | 0/1 (0) |
|  | Intervention type \| Experimental | Experimental intervention type | 1/3 (33.33) | 1/1 (100) | 0/1 (0) |
|  | Trial design \| Placebo arm | Placebo treatment arm | 1/3 (33.33) | 1/1 (100) | 0/1 (0) |
| Orthopaedic surgery (N=1) | Trial design | Trial design | 1/1 (100) | 1/1 (100) | 0/1 (0) |
|  | Follow-up related | Follow-up strategy | 1/1 (100) | 1/1 (100) | 0/1 (0) |
|  | Quality/Risk of bias | Funding sources | 1/1 (100) | 1/1 (100) | 0/1 (0) |
|  | Trial design \| Location(s) | Trial location(s) | 1/1 (100) | 1/1 (100) | 0/1 (0) |
|  | Follow-up related | Follow-up duration | 1/1 (100) | 1/1 (100) | 0/1 (0) |
|  | Publication Type/Year | Impact factor | 1/1 (100) | 0/1 (0) | 1/1 (100) |
|  | Trial design \| Sample size | Sample size | 1/1 (100) | 0/1 (0) | 1/1 (100) |
|  | Eligibility \| Index condition | Index condition type | 1/1 (100) | 0/1 (0) | 1/1 (100) |
| Osteoarthritis (N=1) | Intervention type | Intervention type | 1/1 (100) | 1/1 (100) | 0/1 (0) |
|  | Trial design \| Duration | Trial duration | 1/1 (100) | 1/1 (100) | 0/1 (0) |
|  | Quality/Risk of bias | Methodological quality | 1/1 (100) | 0/1 (0) | 1/1 (100) |
| PTSD (N=6) | Intervention frequency/intensity | Intervention frequency/intensity | 4/6 (66.67) | 2/4 (50) | 2/4 (50) |
|  | Intervention delivery/format | Intervention delivery/format | 4/6 (66.67) | 1/4 (25) | 3/4 (75) |
|  | Trial design \| Sample size | Sample size | 3/6 (50) | 1/3 (33.33) | 2/3 (66.67) |
|  | Intervention type | Intervention type | 3/6 (50) | 2/3 (66.67) | 1/3 (33.33) |
|  | Trial design \| Location(s) | Trial location(s) | 2/6 (33.33) | 1/2 (50) | 1/2 (50) |
|  | Publication Type/Year | Publication/Reporting year | 2/6 (33.33) | 0/2 (0) | 2/2 (100) |
|  | Intervention components | Intervention components | 1/6 (16.67) | 0/1 (0) | 1/1 (100) |
|  | Quality/Risk of bias | Risk of bias | 1/6 (16.67) | 0/1 (0) | 1/1 (100) |
|  | Trial design \| Population | Population studied | 1/6 (16.67) | 0/1 (0) | 1/1 (100) |
|  | Motivational/Incentive strategies | Using incentives | 1/6 (16.67) | 0/1 (0) | 1/1 (100) |
|  | Trial conduct | Involvement of treatment originator | 1/6 (16.67) | 0/1 (0) | 1/1 (100) |
|  | Quality/Risk of bias | Trial quality | 1/6 (16.67) | 0/1 (0) | 1/1 (100) |
|  | Intervention components | Intervention focus | 1/6 (16.67) | 1/1 (100) | 0/1 (0) |
|  | Recruitment-related | Recruitment strategies | 1/6 (16.67) | 0/1 (0) | 1/1 (100) |
| Personality disorders (N=1) | Intervention type | Intervention type | 1/1 (100) | 0/1 (0) | 1/1 (100) |
|  | Intervention duration | Intervention duration | 1/1 (100) | 0/1 (0) | 1/1 (100) |
|  | Intervention frequency/intensity | Intervention frequency/intensity | 1/1 (100) | 0/1 (0) | 1/1 (100) |
|  | Trial design | Trial design | 1/1 (100) | 0/1 (0) | 1/1 (100) |
|  | Intervention components | Intervention components | 1/1 (100) | 0/1 (0) | 1/1 (100) |
|  | Trial conduct | Presence of consultation team | 1/1 (100) | 0/1 (0) | 1/1 (100) |
|  | Eligibility \| Comorbidities | Comorbidities | 1/1 (100) | 0/1 (0) | 1/1 (100) |
| Restless leg syndrome (N=1) | Intervention type | Intervention type | 1/1 (100) | 1/1 (100) | 0/1 (0) |
|  | Intervention type \| Control/Comparator | Control/Comparator intervention type | 1/1 (100) | 0/1 (0) | 1/1 (100) |
|  | Intervention type \| Experimental | Experimental intervention type | 1/1 (100) | 0/1 (0) | 1/1 (100) |
| Rheumatoid arthritis (N=2) | Intervention type | Intervention type | 2/2 (100) | 2/2 (100) | 0/2 (0) |
|  | Trial design \| Duration | Trial duration | 1/2 (50) | 1/1 (100) | 0/1 (0) |
|  | Eligibility criteria | Age limit | 1/2 (50) | 0/1 (0) | 1/1 (100) |
|  | Trial design \| Location(s) | Trial location(s) | 1/2 (50) | 0/1 (0) | 1/1 (100) |
|  | Trial design \| Sample size | Sample size | 1/2 (50) | 0/1 (0) | 1/1 (100) |
| Schizophrenia (N=9) | Trial design \| Duration | Trial duration | 5/9 (55.56) | 3/5 (60) | 2/5 (40) |
|  | Intervention frequency/intensity | Intervention frequency/intensity | 4/9 (44.44) | 2/4 (50) | 2/4 (50) |
|  | Intervention type \| Control/Comparator | Control/Comparator intervention type | 4/9 (44.44) | 2/4 (50) | 2/4 (50) |
|  | Intervention type | Intervention type | 4/9 (44.44) | 2/4 (50) | 2/4 (50) |
|  | Trial design | Trial design | 3/9 (33.33) | 0/3 (0) | 3/3 (100) |
|  | Publication Type/Year | Publication/Reporting year | 2/9 (22.22) | 2/2 (100) | 0/2 (0) |
|  | Trial design \| Location(s) | Trial location(s) | 2/9 (22.22) | 0/2 (0) | 2/2 (100) |
|  | Quality/Risk of bias | Trial quality | 2/9 (22.22) | 1/2 (50) | 1/2 (50) |
|  | Clinician qualifications/training | Clinician qualifications/training | 2/9 (22.22) | 1/2 (50) | 1/2 (50) |
|  | Intervention delivery/format | Intervention delivery/format | 2/9 (22.22) | 1/2 (50) | 1/2 (50) |
|  | Intervention type \| Experimental | Experimental intervention type | 2/9 (22.22) | 2/2 (100) | 0/2 (0) |
|  | Trial design \| Number of sites | Number of sites | 1/9 (11.11) | 1/1 (100) | 0/1 (0) |
|  | Trial design \| Use of run-in | Use of run-in period | 1/9 (11.11) | 0/1 (0) | 1/1 (100) |
|  | Trial design \| Number of arms | Number of arms | 1/9 (11.11) | 0/1 (0) | 1/1 (100) |
|  | Trial conduct | Randomisation rate | 1/9 (11.11) | 0/1 (0) | 1/1 (100) |
|  | Trial design \| Sample size | Sample size | 1/9 (11.11) | 0/1 (0) | 1/1 (100) |
|  | Follow-up related | Follow-up frequency/intensity | 1/9 (11.11) | 0/1 (0) | 1/1 (100) |
|  | Trial design \| Setting | Setting | 1/9 (11.11) | 1/1 (100) | 0/1 (0) |
|  | Motivational/Incentive strategies | Motivational/Incentive strategies | 1/9 (11.11) | 1/1 (100) | 0/1 (0) |
|  | Intervention supervision | Intervention supervision | 1/9 (11.11) | 1/1 (100) | 0/1 (0) |
|  | Intervention duration | Intervention duration | 1/9 (11.11) | 1/1 (100) | 0/1 (0) |
|  | Eligibility \| Index condition | Index condition severity | 1/9 (11.11) | 0/1 (0) | 1/1 (100) |
|  | Trial design | Open label design | 1/9 (11.11) | 0/1 (0) | 1/1 (100) |
|  | Quality/Risk of bias | Reporting method | 1/9 (11.11) | 0/1 (0) | 1/1 (100) |
|  | Quality/Risk of bias | Risk of bias | 1/9 (11.11) | 0/1 (0) | 1/1 (100) |
|  | Eligibility \| Index condition | Index condition type | 1/9 (11.11) | 0/1 (0) | 1/1 (100) |
| Smoking cessation (N=2) | Motivational/Incentive strategies | Using incentives | 2/2 (100) | 1/2 (50) | 1/2 (50) |
|  | Recruitment-related | Recruitment strategies | 1/2 (50) | 1/1 (100) | 0/1 (0) |
|  | Follow-up related | Follow-up duration | 1/2 (50) | 1/1 (100) | 0/1 (0) |
|  | Trial design \| Retention strategies | Use of retention strategies | 1/2 (50) | 0/1 (0) | 1/1 (100) |
|  | Outcome \| Biomarkers | Biomarker assessments | 1/2 (50) | 0/1 (0) | 1/1 (100) |
|  | Outcome \| Primary endpoint | Primary endpoint | 1/2 (50) | 0/1 (0) | 1/1 (100) |
|  | Intervention delivery/format | Intervention delivery/format | 1/2 (50) | 0/1 (0) | 1/1 (100) |
|  | Intervention frequency/intensity | Intervention frequency/intensity | 1/2 (50) | 0/1 (0) | 1/1 (100) |
|  | Intervention components | Pharmacological support | 1/2 (50) | 0/1 (0) | 1/1 (100) |
|  | Trial design | Trial design | 1/2 (50) | 0/1 (0) | 1/1 (100) |
|  | Intervention type \| Experimental | Experimental intervention type | 1/2 (50) | 0/1 (0) | 1/1 (100) |
|  | Intervention components | Number of components | 1/2 (50) | 0/1 (0) | 1/1 (100) |
|  | Intervention type | Intervention type | 1/2 (50) | 0/1 (0) | 1/1 (100) |
| Stroke (N=1) | Intervention type | Intervention type | 1/1 (100) | 0/1 (0) | 1/1 (100) |
| Tinnitus (N=1) | Intervention type | Intervention type | 1/1 (100) | 0/1 (0) | 1/1 (100) |
| Type 2 diabetes (N=2) | Intervention duration | Intervention duration | 1/2 (50) | 1/1 (100) | 0/1 (0) |
|  | Intervention delivery/format | Intervention delivery/format | 1/2 (50) | 0/1 (0) | 1/1 (100) |
|  | Intervention type | Intervention type | 1/2 (50) | 1/1 (100) | 0/1 (0) |
| Vocal rehabilitation (N=1) | Quality/Risk of bias | Trial quality | 1/1 (100) | 1/1 (100) | 0/1 (0) |
|  | Intervention type | Intervention type | 1/1 (100) | 1/1 (100) | 0/1 (0) |
